# Supplementary material for: Asymmetric and Reduced Xanthene Fluorophores: Synthesis, Photochemical Properties, and Application to Activatable Fluorescent Probes for Detection of Nitroreductase
Source: Molecules. 2019 Sep 3;24(17):3206. doi: 10.3390/molecules24173206 (PMC6749439; doi:10.3390/molecules24173206)
Supplement: Supplementary file 1 [file molecules-24-03206-s001.pdf]

SUPPLEMENTARY MATERIALS

Asymmetric and Reduced Xanthene Fluorophores: Synthesis, Photochemical Properties, and Application to Activatable Fluorescent Probes for Detection of Nitroreductase

Kunal N. More [1,†], Tae-Hwan Lim [1,†], Julie Kang [1], Hwayoung Yun [2], Sung-Tae Yee [1] and Dong-Jo Chang [1,*]

[1] College of Pharmacy and Research Institute of Life and Pharmaceutical Sciences, Sunchon National University, Suncheon 57922, Republic of Korea; kunalmore83@gmail.com (K.N.M.); c79852er@naver.com (T.H.L.); juliy19@naver.com (J.K.); sungtae@scnu.ac.kr (S.T.Y.)

[2] College of Pharmacy, Pusan National University, Busan 46241, Korea; hyun@pusan.ac.kr (H.Y.)

* Correspondence: djchang@scnu.ac.kr (D.J.C.); Tel.: +82-61-750-3765 (D.J.C.)

† These two [thors contributed equally to this work.

Academic Editor: David Díez

Table of Contents

A. Synthesis experimental procedures·····S2-S13

B. Spectral data of all compounds·····S14-S54

A. *Synthesis Experimental Procedures*

General information

All reagents and solvents were purchased from Sigma Aldrich Chemical Co. (St. Louis, USA), Tokyo Chemical Industries (Tokyo, Japan), Daejung Chemicals (Siheung-si, Korea), and Alfa Aesar (Ward Hill, USA) and used without any further purification. Anhydrous solvents were purchased from Aldrich Chemical Co. (St. Louis, USA), and all reactions were performed under nitrogen atmosphere. Silica gel (ZEOprep 60 40–63 μm, , Zeochem AG, Kentucky, USA) was used for flash column chromatography, and silica gel plates (Kiesegel 60F_{254}, Merck, Darmstadt, Germany) were used for thin-layer chromatography. ^{1}H and ^{13}C NMR spectra were measured on a JEOL JNM-ECZ400s/L1 (400 MHz) spectrometer (Jeol, Tokyo, Japan), with $CDCl_3$ or DMSO-d_6 as the NMR solvent (Cambridge Isotope Laboratories, Tewksbury, USA). Chemical shifts are expressed in parts per million (ppm), and the coupling constant *J* is reported in hertz (Hz). Chemical shifts (in ppm) in ^{1}H NMR are based on the chemical shift of tetramethylsilane (δ = 0 ppm) in $CDCl_3$ as an internal standard. The chemical shifts in ^{13}C NMR are reported in ppm relative to the centerline of the triplet at 77.0 ppm observed for $CDCl_3$ or 39.5 ppm for DMSO-d_6. Known compounds such as compound **1**, **2**, **3**, **4** and **24** are synthesized as per previously known methods and spectral data is in agreement with previously published data.

1. Methyl 2-(6-methoxy-3-oxo-3*H*-xanthen-9-yl)benzoate (**1**)[1,2,6]

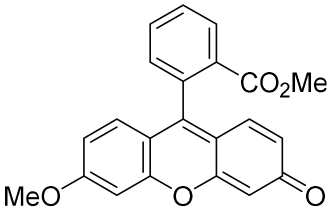

Compound **1** (10.3 g, yellow powder) was synthesized in 99% yield via the alkylation of fluorescein (10 g, 28.6 mmol) using methyl iodide (5.34 mL, 85.8 mmol) and K_2CO_3 (9.88 g, 71.5 mmol) according to general procedure A. ^{1}H-NMR (400 MHz, DMSO-d_6) δ 8.21 (dd, *J* = 8.0, 1.1 Hz, 1H), 7.87 (td, *J* = 7.5, 1.4 Hz, 1H), 7.78 (td, *J* = 7.5, 1.2 Hz, 1H), 7.50 (dd, *J* = 7.5, 1.1 Hz, 1H), 7.23 (d, *J* = 2.7 Hz, 1H), 6.89 (dd, *J* = 8.7, 2.3 Hz, 1H), 6.84 (d, *J* = 9.1 Hz, 1H), 6.80 (d, *J* = 9.6 Hz, 1H), 6.39 (dd, *J* = 9.6, 1.8 Hz, 1H), 6.24 (d, *J* = 1.8 Hz, 1H), 3.91 (s, 3H), 3.58 (s, 3H); ^{13}C-NMR (100 MHz, DMSO- d_6) δ 184.38, 165.72, 164.42, 158.89, 154.10, 150.58, 134.43, 133.73, 131.24, 130.89, 130.58, 130.03, 129.91, 129.38, 117.17, 114.82, 114.10, 105.12, 101.11, 56.82, 52.84; HRMS (ESI$^+$): m/z Calcd for $C_{22}H_{17}O_5$ $[M+H]^+$: 361.1076, Found: 361.1072.

2. Methoxymethyl 2-(6-(methoxymethoxy)-3-oxo-3*H*-xanthen-9-yl)benzoate (**2**)[10]

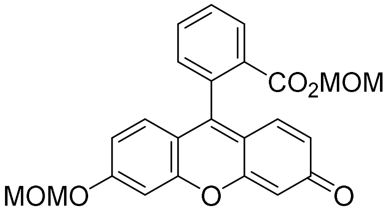

Compound **2** (4.51 g, yellow crystalline powder) was synthesized in 89 % of yield via the alkylation of fluorescein (4 g, 12 mmol) using chloromethyl methyl ether (2.74 mL, 36 mmol) in the presence of K_2CO_3 (3.12 g, 22.56 mmol) according to general procedure A. ^{1}H-NMR (400 MHz, DMSO-d_6) δ 8.25

(dd, J = 7.8, 0.9 Hz, 1H), 7.89 (td, J = 7.5, 1.3 Hz, 1H), 7.80 (td, J = 7.8, 1.3 Hz, 1H), 7.51 (dd, J = 7.3, 0.9 Hz, 1H), 7.25 (d, J = 2.3 Hz, 1H), 6.96 (dd, J = 8.9, 2.5 Hz, 1H), 6.88 (d, J = 8.7 Hz, 1H), 6.82 (d, J = 9.6 Hz, 1H), 6.39 (dd, J = 9.6, 1.8 Hz, 1H), 6.24 (d, J = 1.8 Hz, 1H), 5.35 (s, 2H), 5.14 (q, J = 6.3 Hz, 2H), 3.39 (s, 3H), 3.12 (s, 3H); ^{13}C-NMR (100 MHz, DMSO-d_6) δ 183.94, 164.38, 161.03, 158.30, 153.13, 149.62, 133.79, 133.44, 130.83, 130.25, 129.51, 129.02, 117.10, 115.05, 114.41, 104.67, 102.77, 94.05, 91.16, 56.90, 56.05; HRMS (ESI$^+$): m/z Calcd for $C_{24}H_{21}O_7$ [M+H]$^+$: 421.1243, Found: 421.1278.

3. 6'-Methoxy-3*H*-spiro[isobenzofuran-1,9'-xanthen]-3'-ol (**3**)[13]

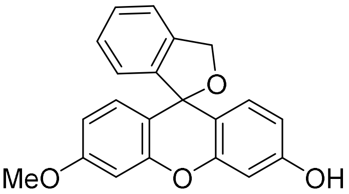

Compound **3** was synthesized from compound **1** (1 g, 2.8 mmol) according to general procedure B. The residue was purified by flash column chromatography on silica gel (CH_2Cl_2/EA = 10:1) to give compound **3** (730 mg, light yellow solid) in 79 % yield over two steps. ^{1}H-NMR (400 MHz, DMSO-d_6) δ 7.44 (d, J = 7.3 Hz, 1H), 7.35 (t, J = 7.3 Hz, 1H), 7.23 (t, J = 7.5 Hz, 1H), 6.80 (d, J = 8.7 Hz, 1H), 6.77 (d, J = 2.7 Hz, 1H), 6.71-6.75 (m, 2H), 6.64 (dd, J = 8.7, 2.3 Hz, 1H), 6.57 (d, J = 2.3 Hz, 1H), 6.50 (dd, J = 8.8, 2.2 Hz, 1H), 5.22 (s, 2H), 3.76 (s, 3H); ^{13}C-NMR (100 MHz, DMSO-d_6) δ 159.81, 158.16, 150.55, 145.36, 138.52, 129.76, 128.09, 123.10, 121.14, 117.35, 115.83, 112.00, 111.04, 101.62, 100.16, 82.59, 71.61, 55.45, 55.18, 54.54; HRMS (ESI$^+$): m/z Calcd for $C_{21}H_{17}O_4$ [M+H]$^+$: 333.1082, Found: 333.1120.

4. 6'-(Methoxymethoxy)-3*H*-spiro[isobenzofuran-1,9'-xanthen]-3'-ol (**4**)[10]

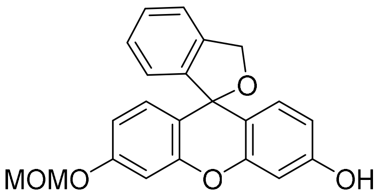

Compound **4** was synthesized from compound **2** (2.5 g, 6.94 mmol) according to general procedure B. The residue was purified by flash column chromatography on silica gel (CH_2Cl_2/EA = 9:1) to afford **4** (1.92 g, yellow powder) in 88% yield over two steps. ^{1}H-NMR (400 MHz, DMSO-d_6) δ 9.82 (s, 1H), 7.44 (d, J = 7.3 Hz, 1H), 7.35 (t, J = 7.1 Hz, 1H), 7.24 (t, J = 7.3 Hz, 1H), 6.86 (d, J = 2.7 Hz, 1H), 6.82 (d, J = 8.7 Hz, 1H), 6.70-6.77 (m, 3H), 6.57 (d, J = 2.5 Hz, 1H), 6.51 (dd, J = 8.5, 2.5 Hz, 1H), 5.23 (s, 2H), 5.20 (s, 2H), 3.36 (s, 3H); ^{13}C-NMR (100 MHz, DMSO-d_6) δ 158.14, 157.13, 150.40, 145.23, 138.50, 129.75, 128.09, 123.10, 121.13, 118.51, 115.73, 112.23, 102.72, 101.61, 93.89, 82.50, 71.63, 55.67; HRMS (ESI$^+$): m/z Calcd for $C_{22}H_{19}O_5$ [M+H]$^+$: 363.1188, Found: 363.1226.

5. 3'-Methoxy-3*H*-spiro[isobenzofuran-1,9'-xanthen]-6'-yl trifluoromethanesulfonate (**5**)

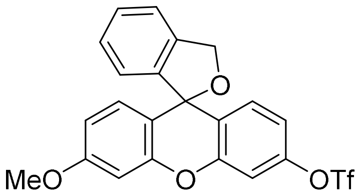

Compound **5** was synthesized from compound **3** (150 mg, 0.45 mmol) according to general procedure C using triflic anhydride (254 mg, 1.80 mmol) and pyridine (0.145 mL, 0.90 mmol) in CH_2Cl_2. The residue was purified by flash column chromatography on silica gel (CH_2Cl_2/EA = 9:1) to give compound **5** (179 mg, yellow gum) in 86% yield. ^{1}H-NMR (400 MHz, DMSO-d_6) δ 7.51 (d, *J* = 2.7 Hz, 1H), 7.49 (d, *J* = 7.3 Hz, 1H), 7.39 (td, *J* = 7.3, 0.9 Hz, 1H), 7.31-7.20 (m, 2H), 7.18 (d, *J* = 8.7 Hz, 1H), 6.91 (d, *J* = 8.7 Hz, 1H), 6.85 (d, *J* = 2.7 Hz, 1H), 6.82 (d, *J* = 7.8 Hz, 1H), 6.73 (dd, *J* = 8.7, 2.7 Hz, 1H), 5.34 (s, 2H), 3.79 (s, 3H); ^{13}C-NMR (100 MHz, DMSO-d_6) δ 160.15, 149.98, 148.63, 144.74, 138.09, 131.02, 129.66, 128.48, 125.96, 122.98, 121.37, 116.66, 111.95, 109.81, 100.26, 81.98, 72.46, 55.53; HRMS (ESI$^+$): m/z Calcd for $C_{22}H_{16}F_3O_6S$ [M+H]$^+$: 465.0575, Found: 465.0616.

6. 3'-(Methoxymethoxy)-3*H*-spiro[isobenzofuran-1,9'-xanthen]-6'-yl trifluoromethanesulfonate (**6**)

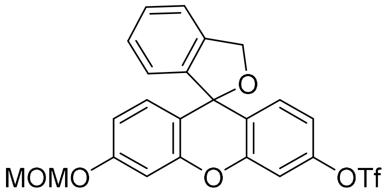

Compound **6** was synthesized from compound **4** (300 mg, 0.83 mmol) according to general procedure C using triflic anhydride (467 mg, 1.66 mmol) and pyridine (265 mg, 3.32 mmol) in CH_2Cl_2. The residue was purified by flash column chromatography on silica gel (CH_2Cl_2/MeOH = 20:1) to give compound **6** (356 mg, yellow oil) in 86% yield. ^{1}H-NMR (400 MHz, DMSO-d_6) δ 7.52 (d, *J* = 2.3 Hz, 1H), 7.49 (d, *J* = 7.3 Hz, 1H), 7.39 (t, *J* = 7.1 Hz, 1H), 7.20-7.30 (m, 2H), 7.18 (d, *J* = 9.1 Hz, 1H), 6.94 (d, *J* = 2.7 Hz, 1H), 6.93 (d, *J* = 3.7 Hz, 1H), 6.79-6.84 (m, 2H), 5.35 (s, 2H), 5.23 (s, 2H), 3.37 (s, 3H); ^{13}C-NMR (100 MHz, DMSO-d_6) δ 157.54, 150.07, 149.76, 148.69, 144.68, 138.14, 131.04, 129.73, 128.59, 128.51, 125.94, 123.04, 121.43, 117.79, 116.84, 113.41, 109.90, 102.83, 93.99, 81.98, 72.56, 55.75; HRMS (ESI$^+$): m/z Calcd for $C_{23}H_{18}F_3O_7S$ [M+H]$^+$: 495.0681, Found: 495.0722.

7. 6'-Methoxy-*N*-propyl-3*H*-spiro[isobenzofuran-1,9'-xanthen]-3'-amine (**7**)

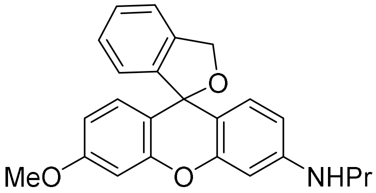

Compound **7** was synthesized via the cross-coupling reaction between compound **5** (100 mg, 0.22 mmol) and *n*-propylamine (0.36 mL, 4.4 mmol) in the presence of $Pd(OAc)_2$ (14.50 mg, 0.022 mmol), BINAP (21.44 mg, 0.034 mmol), and Cs_2CO_3 (210 mg, 0.65 mmol) according to general procedure D. The residue was purified by flash column chromatography on silica gel (CH_2Cl_2/EA = 30:1) to give compound **7** (16.8 mg, light pink powder) in 20% yield. ^{1}H-NMR (400 MHz, DMSO-d_6) δ 7.42 (d, *J* = 7.8 Hz, 1H), 7.34 (td, *J* = 7.5, 0.9 Hz, 1H), 7.23 (t, *J* = 7.1 Hz, 1H), 6.79-6.70 (m, 3H), 6.61 (dd, *J* = 8.7, 2.7 Hz, 1H), 6.57 (d, *J* = 8.7 Hz, 1H), 6.32 (dd, *J* = 8.7, 2.3 Hz, 1H), 6.26 (d, *J* = 2.3 Hz, 1H), 5.91 (t, *J* = 5.3 Hz, 1H), 5.17 (s, 2H), 3.76 (s, 3H), 2.96 (q, *J* = 6.6 Hz, 2H), 1.54 (sext, *J* = 7.3 Hz, 2H), 0.92 (t, *J* = 7.3 Hz, 3H); ^{13}C-NMR (100 MHz, DMSO-d_6) δ 159.68, 150.85, 149.99, 145.46, 138.75, 129.74, 129.19, 128.12, 127.77, 123.16, 121.05, 117.63, 111.68, 110.61, 109.47, 100.12, 96.36, 82.85, 71.25, 55.39, 44.53, 21.76, 11.64; HRMS (ESI$^+$): m/z Calcd for $C_{24}H_{24}NO_3$ [M+H]$^+$: 374.1711, Found: 374.1750.

8. *N*,*N*-Diethyl-6'-methoxy-3*H*-spiro[isobenzofuran-1,9'-xanthen]-3'-amine (**8**)

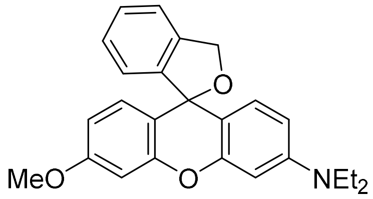

Compound **8** was synthesized via the cross-coupling reaction between compound **5** (650 mg, 1.4 mmol) and diethylamine (1.45 mL, 14 mmol) in the presence of $Pd(OAc)_2$ (94.20 mg, 0.14 mmol), BINAP (131 mg, 0.21 mmol), and Cs_2CO_3 (1.36 g, 4.20 mmol) according to general procedure D. The residue was purified by flash column chromatography on silica gel (CH_2Cl_2/EA = 20:1) to give compound **8** (222 mg, pink powder) in 41% yield. ^{1}H-NMR (400 MHz, DMSO-d_6) 7.43 (d, *J* = 7.8 Hz, 1H), 7.35 (t, *J* = 7.1 Hz, 1H), 7.23 (t, *J* = 7.1 Hz, 1H), 6.84-6.69 (m, 3H), 6.65 (d, *J* = 8.7 Hz, 1H), 6.62 (dd, *J* = 8.7, 2.7 Hz, 1H), 6.41 (dd, *J* = 8.7, 2.7 Hz, 1H), 6.34 (d, *J* = 2.7 Hz, 1H), 5.18 (s, 2H), 3.76 (s, 3H), 3.33 (q, *J* = 6.9 Hz, 4H), 1.07 (t, *J* = 6.9 Hz, 6H); ^{13}C-NMR (100 MHz, DMSO-d_6) δ 159.69, 150.93, 148.13, 145.33, 138.80, 129.64, 127.96, 123.19, 121.04, 117.59, 111.40, 110.57, 108.12, 100.15, 96.67, 82.72, 71.25, 55.36, 43.73, 12.37; HRMS (ESI$^+$): m/z Calcd for $C_{25}H_{26}NO_3$ $[M+H]^+$: 388.1868, Found: 388.1911.

9. *N*-Ethyl-6'-(methoxymethoxy)-3*H*-spiro[isobenzofuran-1,9'-xanthen]-3'-amine (**10**)

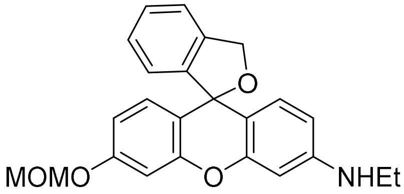

Compound **10** was synthesized via the cross-coupling reaction between compound **6** (100 mg, 0.20 mmol) and ethylamine (2M solution, 2 mL, 4.05 mmol) in anhydrous toluene (3 mL) in the presence of $Pd_2(dba)_3$·$CHCl_3$ (21 mg, 0.02 mmol), Xantphos (18 mg, 0.03 mmol), and Cs_2CO_3 (201 mg, 0.61 mmol) according to procedure D. The residue was purified by flash column chromatography on silica gel to give compound **10** (79 mg, yellow crystalline powder) in 100% yield. ^{1}H-NMR (400 MHz, DMSO-d_6) δ 7.40 (d, *J* = 7.3 Hz, 1H), 7.31 (td, *J* = 7.5, 0.9 Hz, 1H), 7.20 (t, *J* = 7.5 Hz, 1H), 6.80 (d, *J* = 2.7 Hz, 1H), 6.75 (d, *J* = 8.7 Hz, 1H), 6.73 (d, *J* = 7.3 Hz, 1H), 6.66 (dd, *J* = 8.7, 2.3 Hz, 1H), 6.55 (d, *J* = 8.7 Hz, 1H), 6.29 (dd, *J* = 8.2, 2.3 Hz, 1H), 6.23 (d, *J* = 1.8 Hz, 1H), 5.17 (s, 2H), 5.16 (s, 2H), 3.34 (s, 3H), 3.00 (q, *J* = 7.2 Hz, 2H), 1.11 (t, *J* = 7.1 Hz, 3H); ^{13}C-NMR (100 MHz, DMSO-d_6) δ 157.55, 157.55, 151.36, 151.12, 150.44, 145.89, 139.27, 130.28, 129.72, 128.66, 128.33, 123.70, 121.58, 119.32, 112.61, 112.21, 110.10, 103.23, 96.96, 94.41, 83.31, 71.84, 56.18, 37.73, 14.77; HRMS (ESI$^+$): m/z Calcd for $C_{24}H_{24}NO_4$ $[M+H]^+$: 390.1705, Found: 390.1700.

10. *N*,*N*-Diethyl-6'-(methoxymethoxy)-3*H*-spiro[isobenzofuran-1,9'-xanthen]-3'-amine (**11**)

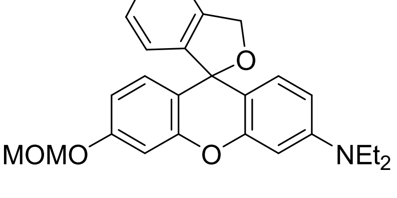

Compound **11** was synthesized via the cross-coupling reaction between compound **6** (100 mg, 0.20 mmol) and diethylamine (0.418 mL, 4.04 mmol) in the presence of $Pd(PPh_3)_4$ (23 mg, 0.02 mmol), BINAP

(20 mg, 0.032 mmol), and Cs_2CO_3 (198 mg, 0.06 mmol) according to procedure D. The residue was purified by flash column chromatography on silica gel (CH_2Cl_2/EA = 30:1) to give the desired product (39 mg, light yellow powder) in 47% yield. ^{1}H-NMR (400 MHz, DMSO-d_6) δ 7.43 (d, *J* = 7.3 Hz, 1H), 7.35 (td, *J* = 7.5, 0.9 Hz, 1H), 7.23 (t, *J* = 7.8 Hz, 1H), 6.83 (d, *J* = 2.3 Hz, 1H), 6.79 (d, *J* = 6.9 Hz, 1H), 6.77 (d, *J* = 5.5 Hz, 1H), 6.69 (dd, *J* = 8.7, 2.3 Hz, 1H), 6.65 (d, *J* = 8.7 Hz, 1H), 6.41 (dd, *J* = 8.9, 2.5 Hz, 1H), 6.35 (d, *J* = 2.3 Hz, 1H), 5.19 (s, 2H), 5.18 (s, 2H), 3.35 (s, 3H), 3.32 (q, *J* = 6.9 Hz, 4H), 1.07 (t, *J* = 6.9 Hz, 6H); ^{13}C-NMR (100 MHz, DMSO-d_6) δ 157.60, 151.51, 151.22, 148.70, 145.76, 139.36, 130.31, 130.06, 128.68, 128.37, 123.75, 121.59, 119.30, 112.59, 111.84, 108.69, 103.29, 97.23, 94.45, 83.20, 71.83, 56.19, 44.26, 12.92; HRMS (ESI$^+$): m/z Calcd for $C_{26}H_{28}NO_4$ [M+H]$^+$: 418.1974, Found: 418.2010.

11. 6'-Methoxy-3*H*-spiro[isobenzofuran-1,9'-xanthen]-3'-amine (**12**)

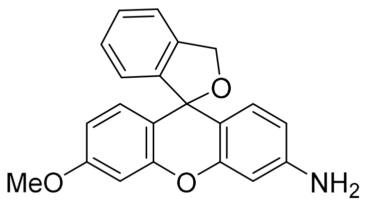

The intermediate imine **9** was synthesized via the cross-coupling reaction between compound **5** (500 mg, 1.08 mmol) and benzophenone imine (236 mg, 1.3 mmol) in the presence of $Pd(OAc)_2$ (74 mg, 0.11 mmol), BINAP (108 mg, 0.17 mmol), and Cs_2CO_3 (1.06 g, 3.23 mmol) according to general procedure D. The crude product (**9**) was used to prepare compound **12** without any further purification. Product **9** was dissolved in THF, followed by the addition of 1 N HCl (3 mL). The reaction mixture was stirred at rt for 30 min, and then, the reaction was quenched with 1 N NaOH solution and extracted with CH_2Cl_2. The organic layer was dried over Na_2SO_4, filtered, and concentrated *in vacuo*. The residue was purified by flash column chromatography on silica gel (CH_2Cl_2/EA = 30:1) to give compound **12** (200 mg, shiny light yellow crystal) in 55% yield over two steps. ^{1}H-NMR (400 MHz, DMSO-d_6) δ 7.42 (d, *J* = 7.3 Hz, 1H), 7.33 (t, *J* = 7.3 Hz, 1H), 7.23 (t, *J* = 7.1 Hz, 1H), 6.73-6.76 (m, 3H), 6.60 (dd, *J* = 8.7, 2.3 Hz, 1H), 6.54 (d, *J* = 8.7 Hz, 1H), 6.33 (d, *J* = 1.8 Hz, 1H), 6.29 (dd, *J* = 8.7, 2.3 Hz, 1H), 5.35 (s, 2H), 5.17 (s, 2H), 3.76 (s, 3H); ^{13}C-NMR (100 MHz, DMSO-d_6) δ 159.66, 150.73, 149.81, 145.46, 138.73, 129.49, 127.92, 123.13, 121.02, 117.59, 112.18, 110.69, 100.09, 98.96, 82.80, 71.22, 55.38; HRMS (ESI$^+$): m/z Calcd for $C_{21}H_{18}NO_3$ [M+H]$^+$: 332.1242, Found: 332.1279.

12. 3'-(Ethylamino)-3*H*-spiro[isobenzofuran-1,9'-xanthen]-6'-ol (**13**)

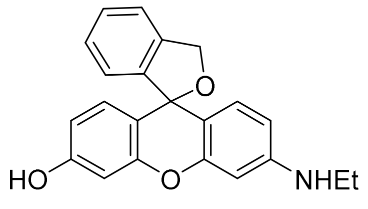

Compound **13** (264 mg, red powder) was synthesized in 55% yield from compound **10** (543 mg) according to general procedure E. ^{1}H-NMR (400 MHz, DMSO-d_6) δ 9.69 (s, 1H), 7.38 (d, *J* = 7.8 Hz, 1H), 7.30 (td, *J* = 7.5, 0.9 Hz, 1H), 7.19 (t, *J* = 7.1 Hz, 1H), 6.71 (d, *J* = 7.8 Hz, 1H), 6.63 (d, *J* = 8.2 Hz, 1H), 6.52 (d, *J* = 8.2 Hz, 1H), 6.49 (d, *J* = 2.3 Hz, 1H), 6.43 (dd, *J* = 8.7, 2.3 Hz, 1H), 6.26 (dd, *J* = 8.7, 2.3 Hz, 1H), 6.21 (d, *J* = 2.3 Hz, 1H), 5.80 (t, *J* = 5.3 Hz, 1H), 5.12 (s, 2H), 2.99 (sext, *J* = 7.3 Hz, 2H), 1.11 (t, *J* = 7.1 Hz, 3H); ^{13}C-NMR (100 MHz, DMSO-d_6) δ 158.43, 151.45, 151.33, 150.34, 146.06, 139.36, 130.28, 129.70, 128.59,

128.19, 123.72, 121.50, 116.74, 112.46, 111.98, 109.98, 102.10, 96.98, 83.47, 71.61, 37.74, 14.79; HRMS (ESI+): m/z Calcd for $C_{22}H_{20}NO_3$ $[M+H]^+$: 346.1443, Found: 346.1439.

13. 3'-(Diethylamino)-3*H*-spiro[isobenzofuran-1,9'-xanthen]-6'-ol (**14**)

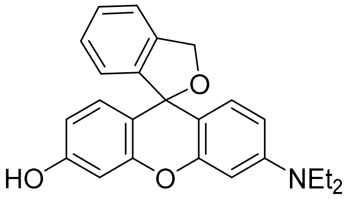

Compound **14** (278 mg, red powder) was synthesized in 84% yield from compound **11** (367 mg) according to general procedure E. ^{1}H-NMR (400 MHz, DMSO-d_6) δ 9.71 (s, 1H), 7.39 (d, *J* = 7.8 Hz, 1H), 7.31 (td, *J* = 7.3, 0.9 Hz, 1H), 7.20 (t, *J* = 7.1 Hz, 1H), 6.73 (d, *J* = 7.3 Hz, 1H), 6.63 (d, *J* = 8.7 Hz, 1H), 6.59 (d, *J* = 8.7 Hz, 1H), 6.51 (d, *J* = 2.3 Hz, 1H), 6.43 (dd, *J* = 8.7, 2.3 Hz, 1H), 6.36 (dd, *J* = 8.9, 2.5 Hz, 1H), 6.31 (d, *J* = 2.1 Hz, 1H), 5.13 (s, 2H), 3.29 (q, *J* = 6.9 Hz, 4H), 1.04 (t, *J* = 7.1 Hz, 6H); ^{13}C-NMR (100 MHz, DMSO- d_6) δ 157.92, 150.97, 148.07, 145.39, 138.89, 129.63, 127.88, 123.21, 120.98, 116.17, 111.51, 107.96, 101.59, 96.77, 82.82, 71.07, 43.69, 12.40; HRMS (ESI+): m/z Calcd for $C_{24}H_{24}NO_3$ $[M+H]^+$: 374.1711, Found: 374.1753.

14. 3'-(Ethylamino)-3*H*-spiro[isobenzofuran-1,9'-xanthen]-6'-yl trifluoromethanesulfonate (**15**)

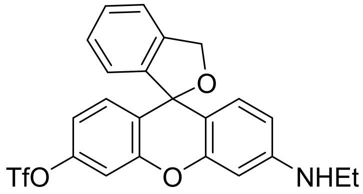

Compound **15** was synthesized from compound **13** (264 mg, 0.76 mmol) using *N*-phenyl-bis-(trifluoromethanesulfonimide) (546 mg, 1.53 mmol) and K_2CO_3 (423 mg, 3.06 mmol) in CH_3CN according to general procedure C. The residue was purified by flash column chromatography on silica gel (CH_2Cl_2/EA = 20:1) to give **15** (250 mg, pink oil) in 69% yield. ^{1}H-NMR (400 MHz, DMSO-d_6) δ 7.47-7.41 (m, 2H), 7.35 (td, *J* = 7.3, 0.9 Hz, 1H), 7.22 (t, *J* = 7.1 Hz, 1H), 7.13 (dd, *J* = 8.9, 2.5 Hz, 1H), 7.06 (d, *J* = 8.7 Hz, 1H), 6.77 (d, *J* = 7.8 Hz, 1H), 6.61 (d, *J* = 8.7 Hz, 1H), 6.34 (dd, *J* = 8.7, 2.3 Hz, 1H), 6.28 (d, *J* = 2.3 Hz, 1H), 5.95 (s, 1H), 5.23 (s, 2H), 3.01 (q, *J* = 6.7 Hz, 2H), 1.12 (t, *J* = 7.1 Hz, 3H) ; ^{13}C-NMR (100 MHz, DMSO- d_6) δ 150.96, 150.91, 150.74, 149.01, 145.37, 139.03, 131.59, 129.65, 128.92, 128.72, 126.77, 123.64, 121.78, 116.73, 111.41, 110.73, 110.23, 96.88, 82.90, 72.41, 40.68, 40.47, 40.26, 40.05, 39.84, 39.64, 39.43, 37.70, 14.72; HRMS (ESI+): m/z Calcd for $C_{23}H_{19}F_3NO_5S$ $[M+H]^+$: 478.0936, Found: 478.0930.

15. 3'-(Diethylamino)-3*H*-spiro[isobenzofuran-1,9'-xanthen]-6'-yl trifluoromethanesulfonate (**16**)

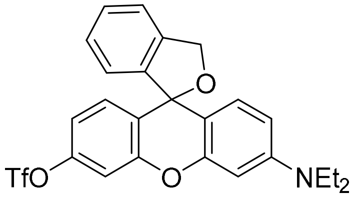

Compound **16** was synthesized from compound **14** (10 mg, 0.03 mmol) using *N*-phenyl-bis-(trifluoromethanesulfonimide) (21 mg, 0.06 mmol) and K_2CO_3 (17 mg, 0.12 mmol) in CH_3CN according

to general procedure C. The residue was purified by flash column chromatography to give **16** (8.5 mg, pink powder) in 57% yield. ^{1}H-NMR (400 MHz, DMSO-d_6) δ 7.44 (d, *J* = 7.8 Hz, 1H), 7.41 (d, *J* = 2.7 Hz, 1H), 7.36 (td, *J* = 7.5, 0.9 Hz, 1H), 7.24 (t, *J* = 3.9 Hz, 1H), 7.14 (dd, *J* = 8.7, 2.7 Hz, 1H), 7.07 (d, *J* = 8.7 Hz, 1H), 6.79 (d, *J* = 7.8 Hz, 1H), 6.68 (d, *J* = 8.7 Hz, 1H), 6.44 (dd, *J* = 9.1, 2.7 Hz, 1H), 6.36 (d, *J* = 2.3 Hz, 1H), 5.22 (s, 2H), 3.31 (q, *J* = 7.8 Hz, 4H), 1.05 (t, *J* = 6.9 Hz, 6H); ^{13}C-NMR (100 MHz, DMSO-d_6) δ 151.0, 149.05, 148.96, 145.23, 139.12, 131.566, 130.00, 128.95, 128.77, 126.74, 123.69, 121.79, 116.76, 111.02, 110.20, 109.32, 97.13, 82.79, 72.40, 44.29, 12.87; HRMS (ESI$^+$): m/z Calcd for $C_{25}H_{23}F_3NO_5S$ [M+H]$^+$: 506.1204, Found: 506.1243

16. *N*$^{3'}$-Ethyl-3*H*-spiro[isobenzofuran-1,9'-xanthene]-3',6'-diamine (**17**)

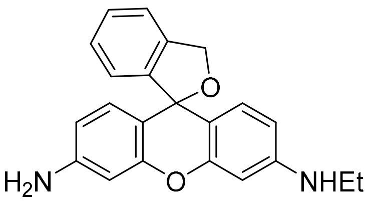

The intermediate imine was synthesized via the cross-coupling reaction between compound **15** (63 mg, 0.13 mmol) and benzophenone imine (35 mg, 0.20 mmol) in anhydrous toluene (1.5 mL) in the presence of Cs_2CO_3 (129 mg, 0.40 mmol), $Pd(OAc)_2$ (9 mg, 0.013 mmol), and BINAP (13 mg, 0.021 mmol) according to general procedure D. The crude imine intermediate was used to prepare compound **17** without any further purification. The crude intermediate was dissolved in THF (2 mL), followed by the addition of 1 N HCl (0.5 mL), and the reaction mixture was stirred at rt for 30 min. The reaction was quenched with 1 N NaOH solution and extracted with CH_2Cl_2. The organic layer was dried over Na_2SO_4, filtered, and concentrated *in vacuo*. The residue was purified by flash column chromatography on silica gel (CH_2Cl_2/EA = 20:1) to give **17** (20 mg, dark red powder) in 44% yield. ^{1}H-NMR (400 MHz, DMSO-d_6) δ 7.36 (d, *J* = 7.3 Hz, 1H), 7.29 (td, *J* = 7.4, 1.1 Hz, 1H), 7.19 (t, *J* = 7.3 Hz, 1H), 6.70 (d, *J* = 7.3 Hz, 1H), 6.48 (d, *J* = 8.7 Hz, 1H), 6.45 (d, *J* = 8.2 Hz, 1H), 6.29-6.17 (m, 4H), 5.74 (t, *J* = 5.3 Hz, 1H), 5.26 (s, 2H), 5.08 (s, 2H), 2.99 (sext, *J* = 7.3 Hz, 2H), 1.11 (t, *J* = 7.1 Hz, 3H); ^{13}C-NMR (100 MHz, DMSO- d_6) δ 151.63, 151.48, 150.21, 150.09, 146.20, 139.56, 129.82, 129.69, 128.46, 128.01, 123.76, 121.417, 113.23, 112.83, 110.81, 109.65, 99.59, 97.07, 83.73, 71.29, 37.76, 14.82; HRMS (ESI$^+$): m/z Calcd for $C_{22}H_{21}N_2O_2$ [M+H]$^+$: 345.1603, Found: 345.1597.

17. *N*$^{3'}$,*N*$^{3'}$-Diethyl-3*H*-spiro[isobenzofuran-1,9'-xanthene]-3',6'-diamine (**18**)

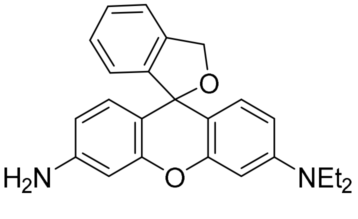

The intermediate imine was synthesized via the cross-coupling reaction between compound **16** (90 mg, 0.18 mmol) and benzophenone imine (39.9 mg, 0.22 mmol) in anhydrous toluene (5 mL) in the presence of Cs_2CO_3 (176 mg, 0.54 mmol), $Pd(OAc)_2$ (13.5 mg, 0.02 mmol), and BINAP (18.7 mg, 0.03 mmol) according to general procedure D. The crude imine intermediate was used to prepare compound **18**, without any further purification. The crude intermediate was dissolved in THF (2 mL) followed by the addition of 1 N HCl (3 mL), and the reaction mixture was stirred at rt for 2 h. The reaction was quenched with 1 N NaOH solution and extracted with CH_2Cl_2. The organic layer was

dried over Na_2SO_4, filtered, and concentrated *in vacuo*. The residue was purified by flash column chromatography on silica gel (CH_2Cl_2/EA = 7:3) to give **18** (20 mg, dark red powder) in 30% yield. ^{1}H-NMR (400 MHz, DMSO-d_6) δ 7.40 (d, *J* = 7.8 Hz, 1H), 7.32 (td, *J* = 7.3, 0.9 Hz, 1H), 7.22 (t, *J* = 7.1 Hz, 1H), 6.74 (d, *J* = 7.3 Hz, 1H), 6.58 (d, *J* = 8.7 Hz, 1H), 6.49 (d, *J* = 8.2 Hz, 1H), 6.35 (dd, *J* = 8.7, 2.7 Hz, 1H), 6.30 (t, *J* = 2.7 Hz, 2H), 6.25 (dd, *J* = 8.7, 2.3 Hz, 1H), 5.29 (s, 2H), 5.11 (s, 2H), 3.32 (q, *J* = 10.7 Hz, 4H), 1.07 (t, *J* = 7.1 Hz, 6H); ^{13}C-NMR (100 MHz, DMSO- d$_6$) δ 151.79, 151.58, 150.13, 148.51, 146.07, 139.63, 130.03, 129.85, 128.49, 128.06, 123.79, 121.44, 113.20, 112.49, 110.84, 108.21, 99.61, 97.38, 83.62, 71.29, 44.20, 12.96; HRMS (ESI$^+$): m/z Calcd for $C_{24}H_{25}N_2O_2$ $[M+H]^+$: 373.1871, Found: 373.1911.

18. 3',6'-Dimethoxy-3*H*-spiro[isobenzofuran-1,9'-xanthene] (**19**)

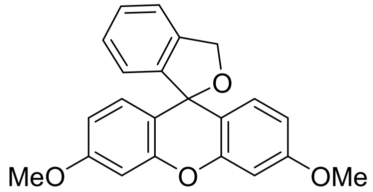

To a solution of compound **3** (20 mg, 0.06 mmol) in DMF (1.5 mL) were added K_2CO_3 (12.5 mg, 0.09 mmol) and methyl iodide (0.01 mL, 0.07 mmol), and the reaction mixture was stirred at rt for 2 h. After completion of the reaction, ice-water was added to the reaction mixture and stirred at 0 °C for 30 min. The resulting yellow solid was filtered and washed with water to completely remove K_2CO_3. The solid was dried to afford **19** (18 mg, light yellow powder) in 86% yield. ^{1}H-NMR (400 MHz, DMSO-d_6) 7.42 (d, *J* = 7.3 Hz, 1H), 7.33 (td, *J* = 7.4, 1.1 Hz, 1H), 7.21 (t, *J* = 7.1 Hz, 1H), 6.81 (d, *J* = 8.7 Hz, 2H), 6.75 (d, *J* = 2.3 Hz, 2H), 6.72 (d, *J* = 7.8 Hz, 1H), 6.64 (dd, *J* = 8.7, 2.7 Hz, 2H), 5.23 (s, 2H), 3.74 (s, 6H); ^{13}C-NMR (100 MHz, DMSO-d_6) δ 160.38, 151.00, 145.83, 138.92, 130.30, 128.83, 128.56, 123.58, 121.72, 117.77, 111.66, 100.67, 82.97, 72.33, 55.97; HRMS (ESI$^+$): m/z Calcd for $C_{22}H_{19}O_4$ $[M+H]^+$: 347.1283, Found: 347.1274.

19. 3'-(Benzyloxy)-6'-methoxy-3*H*-spiro[isobenzofuran-1,9'-xanthene] (**20**)

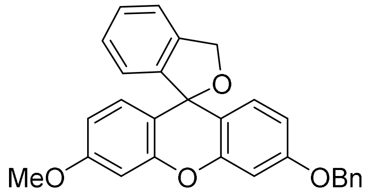

To a solution of compound **3** (20 mg, 0.06 mmol) in acetone (2.5 mL) were added DBU (0.009 mL mg, 0.09 mmol) and benzyl bromide (0.012 mL, 0.07 mmol), and the reaction mixture was stirred at rt for 10 min. The reaction mixture was concentrated *in vacuo*, and the crude residue was purified by flash column chromatography on silica gel (Hex/EA = 9:1) to afford **20** (25 mg, light yellow powder) in 98% yield. ^{1}H-NMR (400 MHz, DMSO-d_6) δ 7.43-7.39 (m, 3H), 7.39-7.27 (m, 4H), 7.21 (t, *J* = 7.5 Hz, 1H), 6.81 (dd, *J* = 8.5, 2.5 Hz, 3H), 6.77-6.69 (m, 3H), 6.64 (dd, *J* = 8.9, 2.5 Hz, 1H), 5.23 (s, 2H), 5.11 (s, 2H), 3.74 (s, 3H); ^{13}C-NMR (100 MHz, DMSO-d_6) δ 160.40, 159.40, 151.00, 150.93, 145.79, 138.94, 137.30, 130.33, 129.02, 128.82, 128.56, 128.46, 128.24, 123.60, 121.71, 118.01, 117.73, 112.39, 111.72, 101.62, 100.68, 82.95, 72.33, 70.01, 55.98; HRMS (ESI$^+$): m/z Calcd for $C_{28}H_{23}O_4$ $[M+H]^+$: 423.1596, Found: 423.1587.

20. *N*-(3'-Methoxy-3*H*-spiro[isobenzofuran-1,9'-xanthen]-6'-yl)benzamide (**21**)

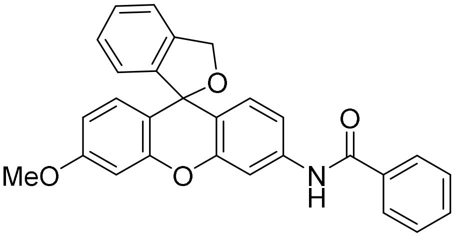

To a solution of compound **12** (20 mg, 0.06 mmol) in DMF (1.5 mL) were added EDC (15 mg, 0.08 mmol), HOBt (11 mg. 0.08 mmol), *i*$PrNEt_2$ (0.021 mL, 0.12 mmol), and benzoic acid (8.5 mg, 0.07 mmol), and the reaction mixture stirred at rt for 12 h. The reaction mixture was extracted with CH_2Cl_2. The organic layer was dried over Na_2SO_4, filtered, and concentrated *in vacuo*. The crude residue was purified by flash column chromatography on silica gel (Hex/EA = 1:1) to afford **21** (13 mg, white crystalline powder) in 50% yield. ^{1}H-NMR (400 MHz, DMSO-*d*$_6$) δ 10.38 (s, 1H), 7.95-7.88 (m, 2H), 7.84 (d, *J* = 1.8 Hz, 1H), 7.61-7.54 (m, 1H), 7.53-7.46 (m, 2H), 7.44 (d, *J* = 7.3 Hz, 1H), 7.39 (dd, *J* = 8.7, 1.8 Hz, 1H), 7.34 (td, *J* = 7.3, 0.9 Hz, 1H), 7.22 (t, *J* = 7.1 Hz, 1H), 6.92 (d, *J* = 8.7 Hz, 1H), 6.84 (d, *J* = 8.7 Hz, 1H), 6.82 (d, *J* = 2.7 Hz, 1H), 6.75 (d, *J* = 7.8 Hz, 1H), 6.65 (dd, *J* = 8.7, 2.7 Hz, 1H), 5.28 (s, 2H), 3.76 (s, 3H); ^{13}C-NMR (100 MHz, DMSO-*d*$_6$) δ 166.39, 160.46, 151.04, 150.03, 145.93, 140.46, 138.79, 135.24, 132.30, 130.21, 129.45, 128.97, 128.85, 128.60, 128.25, 123.55, 121.77, 120.70, 117.71, 116.40, 111.84, 107.42, 100.75, 82.94, 72.57, 56.02, 55.44; HRMS (ESI$^+$): m/z Calcd for $C_{28}H_{22}NO_4$ $[M+H]^+$: 436.1549, Found: 436.1543.

21. Methyl 2-(6-methoxy-3-oxo-3*H*-xanthen-9-yl)benzoate (**24**)

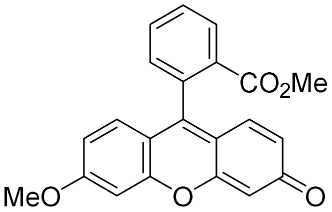

To a solution of compound **22** (20 mg, 0.06 mmol) in DMF (1.5 mL) were added K_2CO_3 (12.5 mg, 0.09 mmol) and methyl iodide (10 mg, 0.07 mmol), and the reaction mixture was stirred at rt for 10 min. After completion of the reaction, ice-water was added to the reaction mixture and stirred at 0 °C for 30 min. The resulting yellow solid was filtered and washed with water to completely remove the K_2CO_3 reagent. The solid was dried to afford **24** (18 mg, yellow powder) in 87% yield. ^{1}H NMR (400 MHz, DMSO-*d*$_6$) δ 8.18 (dd, *J* = 7.8, 1.4 Hz, 1H), 7.83 (td, *J* = 7.5, 1.4 Hz, 1H), 7.75 (td, *J* = 7.5, 1.4 Hz, 1H), 7.46 (dd, *J* = 7.5, 1.1 Hz, 1H), 7.19 (d, *J* = 2.3 Hz, 1H), 6.86 (dd, *J* = 9.1, 2.3 Hz, 1H), 6.80 (d, *J* = 8.7 Hz, 1H), 6.76 (d, *J* = 10.1 Hz, 1H), 6.35 (dd, *J* = 9.6, 1.8 Hz, 1H), 6.21 (d, *J* = 1.8 Hz, 1H), 3.87 (s, 3H), 3.54 (s, 3H); ^{13}C-NMR (100 MHz, DMSO-*d*$_6$) δ 184.41, 165.73, 164.44, 158.92, 154.11, 150.67, 134.42, 133.75, 131.23, 130.91, 130.59, 130.02, 129.89, 129.39, 117.16, 114.82, 114.13, 105.10, 101.11, 56.82, 52.84; HRMS (ESI$^+$): m/z Calcd for $C_{22}H_{17}O_5$ $[M+H]^+$: 361.1076, Found: 361.1072.

22. Benzyl 2-(6-methoxy-3-oxo-3*H*-xanthen-9-yl)benzoate (**25**)

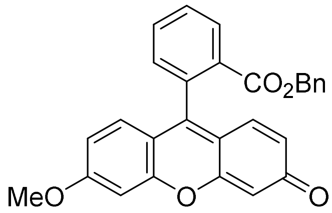

To a solution of compound **22** (20 mg, 0.06 mmol) in acetone (2.5 mL) were added DBU (13 mg, 0.09 mmol) and benzyl bromide (12 mg, 0.07 mmol), and the reaction mixture was stirred at rt for 10 min. The reaction mixture was concentrated *in vacuo*, and the crude residue was purified by flash column chromatography on silica gel (Hex/EA = 9:1) to afford **25** (18 mg, yellow powder) in 71% yield. ^{1}H-NMR (400 MHz, DMSO-d_6) δ 8.19 (dd, *J* = 7.8, 1.4 Hz, 1H), 7.82 (td, *J* = 7.5, 1.4 Hz, 1H), 7.75 (td, *J* = 7.5, 1.4 Hz, 1H), 7.44 (dd, *J* = 7.5, 1.1 Hz, 1H), 7.30-7.20 (m, 1H), 7.20-7.12 (m, 2H), 7.06 (d, *J* = 2.3 Hz, 1H), 6.99-6.92 (m, 2H), 6.81 (dd, *J* = 8.7, 2.3 Hz, 1H), 6.78 (d, *J* = 9.1 Hz, 1H), 6.75 (d, *J* = 9.6 Hz, 1H), 6.33 (dd, *J* = 9.8, 2.1 Hz, 1H), 6.11 (d, *J* = 1.8 Hz, 1H), 4.94 (dd, *J* = 16.2, 12.1 Hz, 2H), 3.87 (s, 3H); ^{13}C-NMR (100 MHz, DMSO-d_6) δ 184.35, 165.45, 164.36, 158.73, 154.01, 150.16, 135.30, 134.09, 133.73, 131.39, 131.19, 130.81, 130.58, 130.21, 129.93, 129.33, 128.83, 128.65, 128.32, 117.24, 114.78, 114.03, 105.09, 101.07, 67.32, 56.80; HRMS (ESI$^+$): m/z Calcd for $C_{28}H_{21}O_5$ [M+H]$^+$: 437.1389, Found: 437.1381.

23. *N*-(3'-Methoxy-3-oxo-3*H*-spiro[isobenzofuran-1,9'-xanthen]-6'-yl)benzamide (**26**)

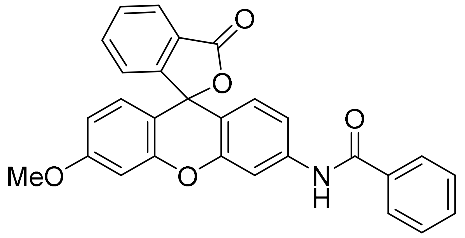

To a solution of compound **23** (20 mg, 0.06 mmol) in DMF (1.5 mL) were added EDC (15 mg, 0.08 mmol), HOBt (11 mg. 0.08 mmol), *i*PrNEt$_2$ (0.021 mL, 0.12 mmol), and benzoic acid (8.5 mg, 0.07 mmol), and the reaction mixture stirred at rt for 12 h. The reaction mixture was extracted with CH_2Cl_2, and the organic layer dried over Na_2SO_4, filtered, and concentrated *in vacuo*. The crude residue was purified by flash column chromatography on silica gel (Hex/EA = 1:1) to afford **26** (2 mg, light yellow powder) in 8% yield. ^{1}H-NMR (400 MHz, DMSO-d_6) δ 10.49 (s, 1H), 8.04-7.97 (m, 2H), 7.97-7.88 (m, 2H), 7.77 (td, *J* = 7.5, 1.2 Hz, 1H), 7.70 (td, *J* = 7.5, 0.9 Hz, 1H), 7.58 (tt, *J* = 7.3, 1.8 Hz, 1H), 7.54-7.48 (m, 2H), 7.42 (dd, *J* = 8.7, 2.3 Hz, 1H), 7.26 (d, *J* = 7.3 Hz, 1H), 6.97 (d, *J* = 2.3 Hz, 1H), 6.76 (d, *J* = 8.7 Hz, 1H), 6.70 (dd, *J* = 9.1, 2.3 Hz, 1H), 6.65 (d, *J* = 9.1 Hz, 1H), 3.79 (s, 3H); ^{13}C-NMR (100 MHz, DMSO-d_6) δ 169.22, 166.56, 161.66, 153.11, 152.35, 151.28, 141.93, 136.31, 135.06, 132.43, 130.79, 129.51, 128.99, 128.84, 128.32, 126.31, 125.77, 125.33, 124.52, 116.88, 114.10, 112.68, 111.28, 107.72, 101.41, 82.67, 56.24; HRMS (ESI$^+$): m/z Calcd for $C_{28}H_{20}NO_5$ [M+H]$^+$: 450.1341, Found: 450.1338.

24. 3'-Methoxy-6'-((4-nitrobenzyl)oxy)-3*H*-spiro[isobenzofuran-1,9'-xanthene] (**27**).

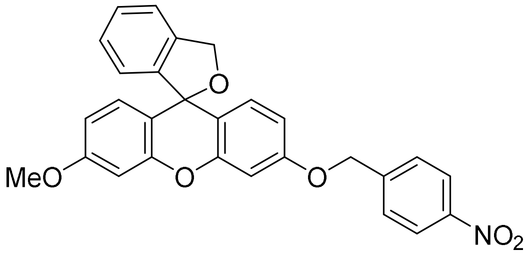

To a mixture of compound **3** (50 mg, 0.15 mmol) and 4-nitrobenzyl bromide (39 mg, 0.18 mmol) in toluene (5 mL) was added silver(I)oxide (52 mg, 0.23 mmol). The reaction mixture was heated under reflux with stirring for 5 h. The reaction mixture was filtered through a short pad of Celite, which was subsequently washed with CH_2Cl_2. The filtrate was concentrated *in vacuo,* and the residue was purified by flash column chromatography on silica gel (Hex/EA = 3:1) to afford **27** (38 mg, white powder) in 54% yield. ^{1}H NMR (400 MHz, DMSO-d_6) δ 8.26 (d, *J* = 9.1 Hz, 2H), 7.72 (d, *J* = 9.1 Hz, 2H), 7.45 (d, *J* = 7.8 Hz, 1H), 7.36 (td, *J* = 7.5, 0.9 Hz, 1H), 7.23 (t, *J* = 7.1 Hz, 1H), 6.83-6.88 (m, 3H), 6.77-6.79 (m, *J* = 2.2 Hz, 2H), 6.75 (d, 1H), 6.67 (dd, *J* = 8.9, 2.5 Hz, 1H), 5.33 (s, 2H), 5.26 (s, 2H), 3.77 (s, 3H); ^{13}C-NMR (100 MHz, DMSO-d_6) δ 159.88, 158.42, 150.41, 147.05, 145.20, 144.71, 138.39, 129.84, 128.04-128.29, 123.67, 123.03, 121.18, 117.87, 117.17, 111.84, 111.20, 101.23, 100.15, 82.38, 71.82, 68.27, 55.45; HRMS (ESI$^+$): m/z Calcd for $C_{28}H_{22}NO_6$ [M+H]$^+$: 468.1402, Found: 468.1441.

25. *N*,*N*-Diethyl-6'-((4-nitrobenzyl)oxy)-3*H*-spiro[isobenzofuran-1,9'-xanthen]-3'-amine (**28**)

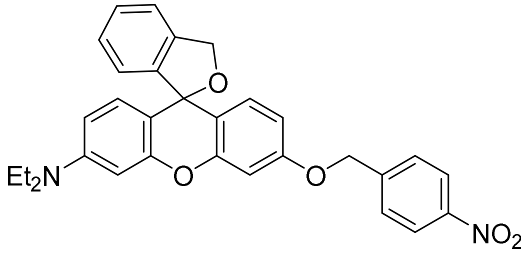

To a mixture of compound **14** (20 mg, 0.05 mmol) and 4-nitrobenzyl bromide (14 mg, 0.014 mmol) in toluene (5 mL) was added silver(I)oxide (19 mg, 0.08 mmol), and the reaction mixture was heated under reflux with stirring for 5 h. The reaction mixture was filtered through a short pad of Celite, which was subsequently washed with CH_2Cl_2. The filtrate was concentrated *in vacuo*, and the residue was purified by flash column chromatography on silica gel (Hex/EA = 3:1) to afford **28** (13 mg, red oil) in 48% yield. ^{1}H-NMR (400 MHz, DMSO-d_6) δ 8.23 (d, *J* = 8.7 Hz, 2H), 7.68 (d, *J* = 9.1 Hz, 2H), 7.41 (d, *J* = 7.8 Hz, 1H), 7.32 (td, *J* = 7.3, 0.9 Hz, 1H), 7.20 (t, *J* = 7.1 Hz, 1H), 6.80 (d, *J* = 2.3 Hz, 1H), 6.79-6.68 (m, 3H), 6.62 (d, *J* = 8.7 Hz, 1H), 6.39 (dd, *J* = 8.7, 2.7 Hz, 1H), 6.30 (d, *J* = 2.3 Hz, 1H), 5.29 (s, 2H), 5.15 (s, 2H), 3.29 (q, *J* = 6.9 Hz, 4H), 1.04 (t, *J* = 6.9 Hz, 6H); ^{13}C-NMR (100 MHz, DMSO-d_6) δ 158.80, 151.47, 151.36, 148.70, 147.59, 145.77, 145.34, 139.35, 130.45, 130.07, 128.76, 128.69, 128.37, 124.20, 123.72, 121.60, 118.75, 111.85, 101.78, 97.20, 71.82, 68.76, 44.26; HRMS (ESI$^+$): m/z Calcd for $C_{31}H_{29}N_2O_5$ [M+H]$^+$: 509.2076, Found: 509.2070.

26. 4-Nitrobenzyl (3'-methoxy-3*H*-spiro[isobenzofuran-1,9'-xanthen]-6'-yl)carbamate (**29**)

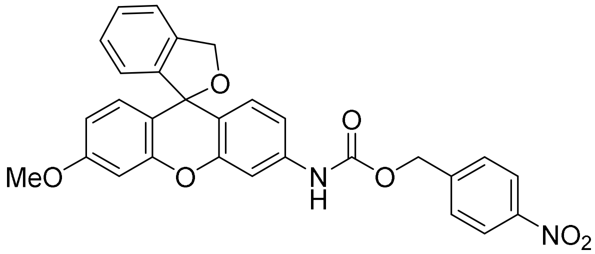

To a solution of compound **12** (20 mg, 0.06 mmol) in CH_2Cl_2 (4 mL) at 0 °C was added a solution of *i*PrNEt$_2$ (20.90 mg, 0.12 mmol) in CH_2Cl_2 (2 mL), followed by a solution of 4-nitrobenzyl chloroformate (15.52 mg, 0.07 mmol) in CH_2Cl_2 (2 mL), and the reaction mixture was stirred at 0 °C for 20 min. The reaction mixture was allowed to warm to rt and stirred at rt for 5 h. The reaction was concentrated *in vacuo*, and the residue was purified by flash column chromatography on silica gel to afford **29** (27 mg, white crystalline powder) in 86% yield. ^{1}H-NMR (400 MHz, DMSO-*d*$_6$) δ 10.10 (s, 1H), 8.26 (d, *J* = 11.4 Hz, 2H), 7.69 (d, *J* = 8.7 Hz, 2H), 7.45 (t, *J* = 3.2 Hz, 2H), 7.36 (t, *J* = 7.5 Hz, 1H), 7.23 (t, *J* = 7.3 Hz, 1H), 7.13 (dd, *J* = 8.7, 2.3 Hz, 1H), 6.88 (d, *J* = 8.7 Hz, 1H), 6.85 (d, *J* = 8.7 Hz, 1H), 6.82 (d, *J* = 2.7 Hz, 1H), 6.75 (d, *J* = 7.8 Hz, 1H), 6.66 (dd, *J* = 8.7, 2.3 Hz, 1H), 5.31 (s, 2H), 5.27 (s, 2H), 3.77 (s, 3H); ^{13}C-NMR (100 MHz, DMSO-*d*$_6$) δ 160.43, 153.55, 150.99, 150.28, 147.65, 145.86, 144.91, 140.27, 138.82, 130.20, 129.75, 129.06, 128.82, 128.56, 124.17, 123.54, 121.73, 119.79, 117.74, 114.55, 111.78, 105.33, 100.76, 82.90, 72.48, 65.20, 56.00; HRMS (ESI$^+$): m/z Calcd for $C_{29}H_{23}N_2O_7$ [M+H]$^+$: 511.1505, Found: 511.1501.

27. 4-Nitrobenzyl (3'-(diethylamino)-3*H*-spiro[isobenzofuran-1,9'-xanthen]-6'-yl)carbamate (**30**)

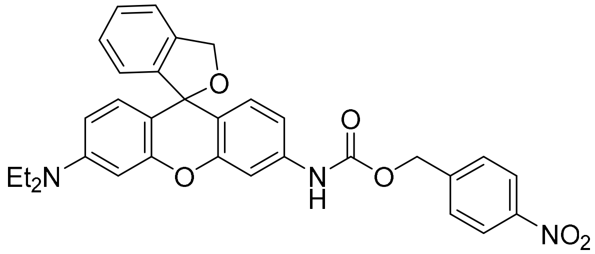

To a solution of compound **18** (20 mg, 0.05 mmol) in anhydrous CH_2Cl_2 (2.5 mL) were added *i*PrNEt$_2$ (12.9 mg, 0.1 mmol) and 4-nitrobenzyl chloroformate (12.9 mg, 0.06 mmol), and the reaction mixture was stirred at 0 °C for 20 min. The reaction mixture was allowed to warm to rt and stirred at rt for 6 h. The reaction was quenched with water and extracted with CH_2Cl_2. The organic layer was dried over Na_2SO_4 and concentrated *in vacuo*. The residue was purified by flash chromatography on silica gel (CH_2CH_2/EA = 9:1) to afford **30** (13 mg, yellow powder) in 47% yield. ^{1}H-NMR (400 MHz, DMSO-*d*$_6$) δ 10.03 (s, 1H), 8.23 (d, *J* = 9.1 Hz, 2H), 7.66 (d, *J* = 19.7 Hz, 2H), 7.40 (d, *J* = 7.8 Hz, 1H), 7.37 (s, 1H), 7.32 (t, *J* = 7.1 Hz, 1H), 7.20 (t, *J* = 7.1 Hz, 1H), 7.07 (dd, *J* = 8.7, 2.3 Hz, 1H), 6.78 (d, *J* = 8.7 Hz, 1H), 6.72 (d, 1H), 6.63 (d, *J* = 9.1 Hz, 1H), 6.34-6.39 (m, 2H), 5.28 (s, 2H), 5.17 (s, 2H), 3.26 (q, *J* = 6.9 Hz, 4H), 1.04 (t, *J* = 6.9 Hz, 6H); ^{13}C-NMR (100 MHz, DMSO-*d*$_6$) δ 153.01, 150.95, 150.16, 148.19, 147.09, 145.33, 144.40, 138.71, 129.34, 128.52, 127.97, 123.62, 123.14, 121.07, 119.59, 113.55, 111.37, 108.12, 104.85, 96.80, 82.62, 71.40, 64.63, 43.68, 12.40; HRMS (ESI$^+$): m/z Calcd for $C_{32}H_{30}N_3O_6$ [M+H]$^+$: 552.2090, Found: 552.2129.

B. Spectral data of all compounds

^{1}H NMR

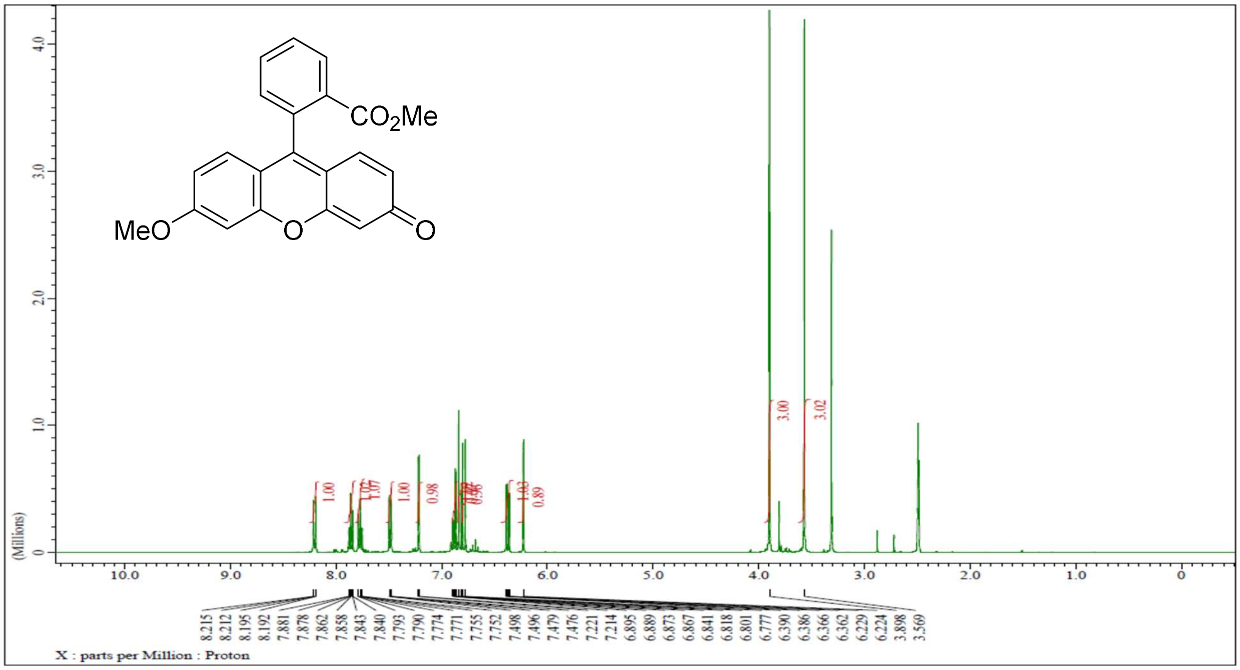

^{13}C NMR

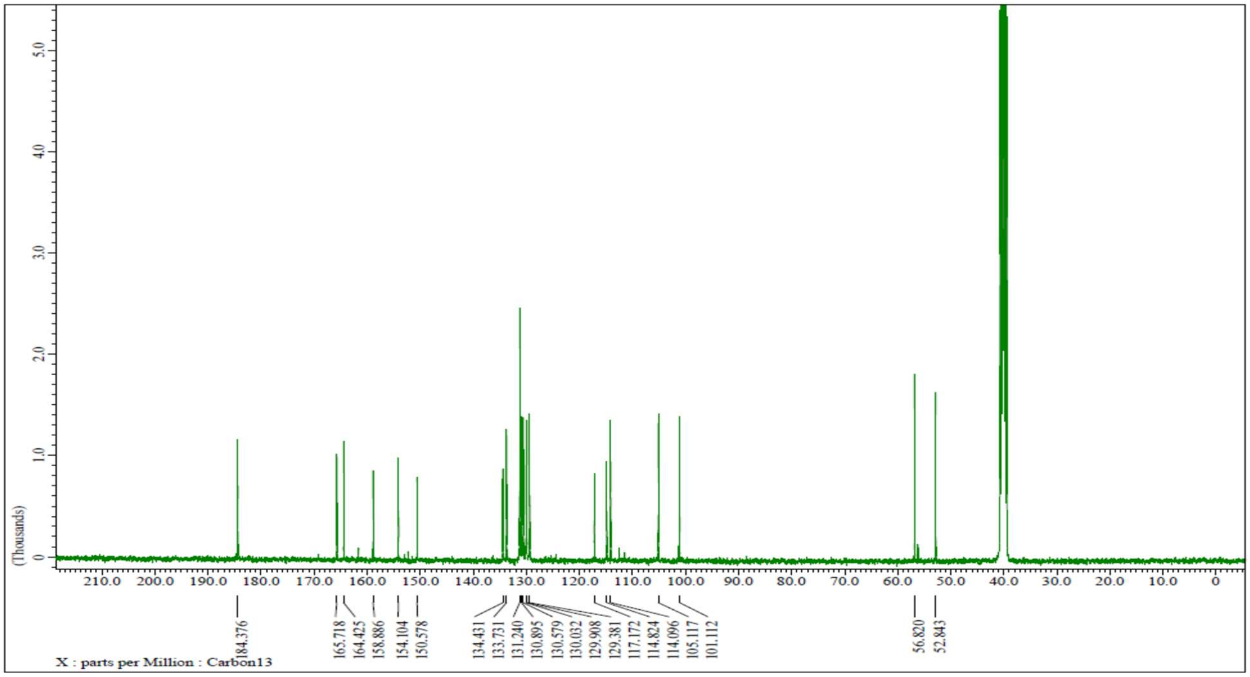

Mass Spectra

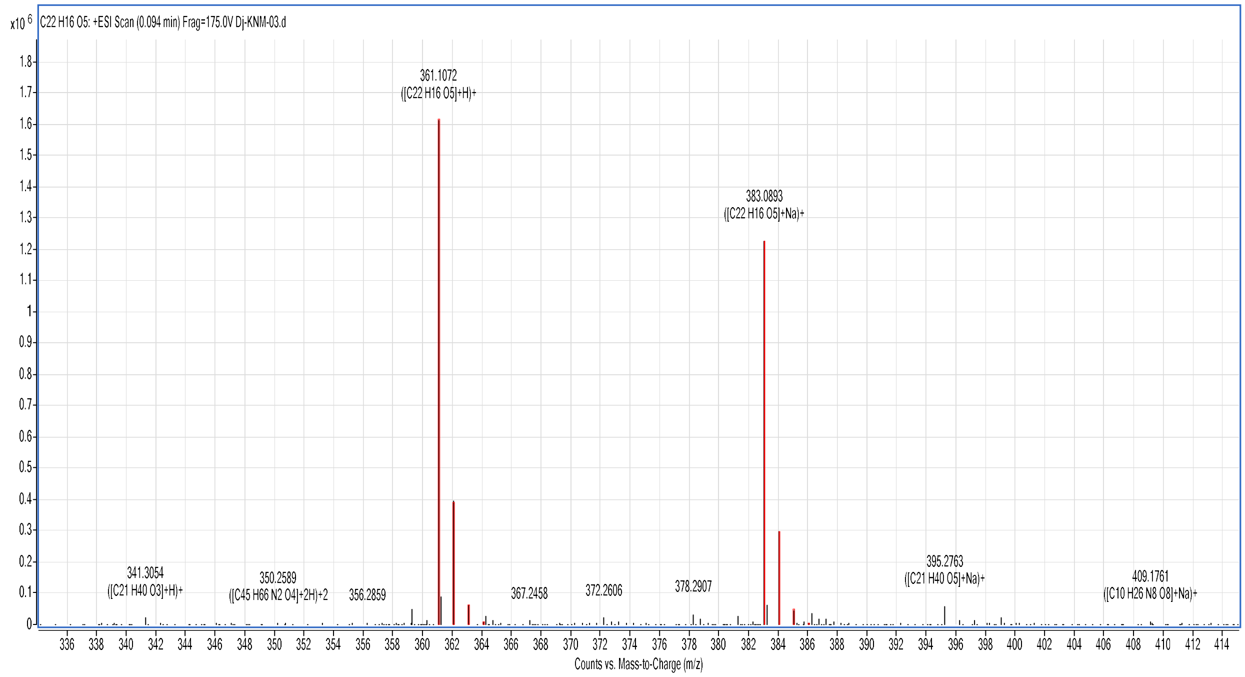

Figure S1: Structure, ^{1}H NMR, ^{13}C NMR and HRMS of Compound 1.

^{1}H NMR

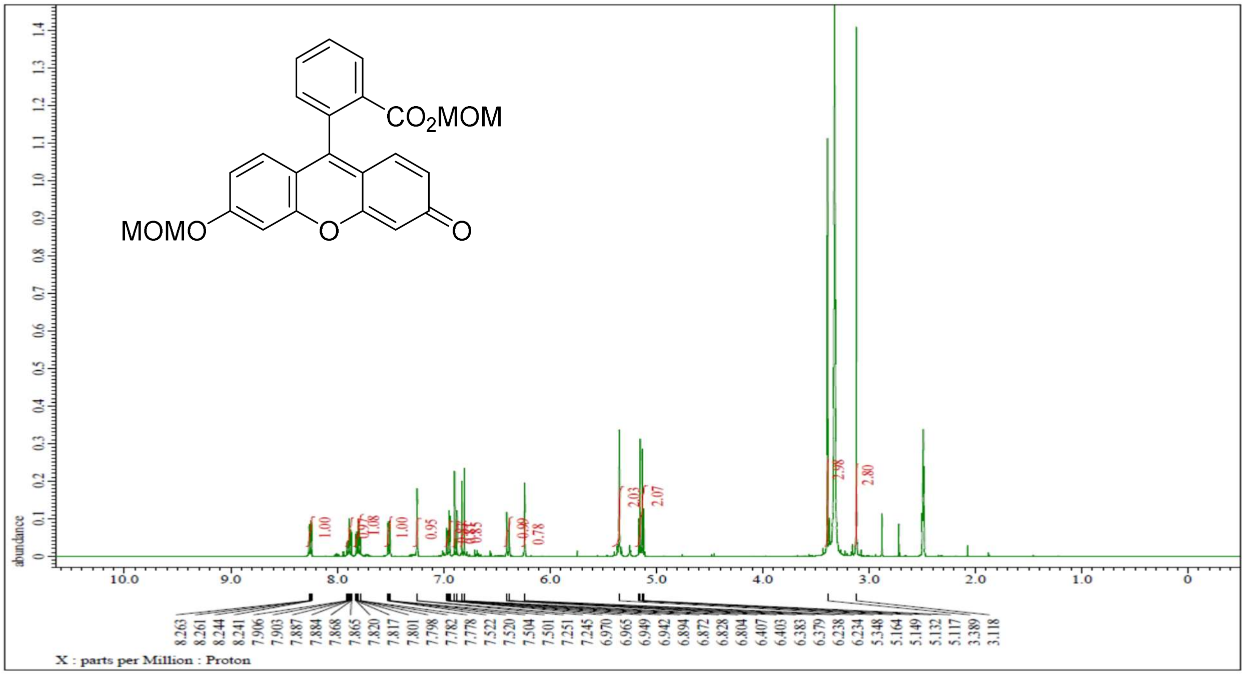

^{13}C NMR

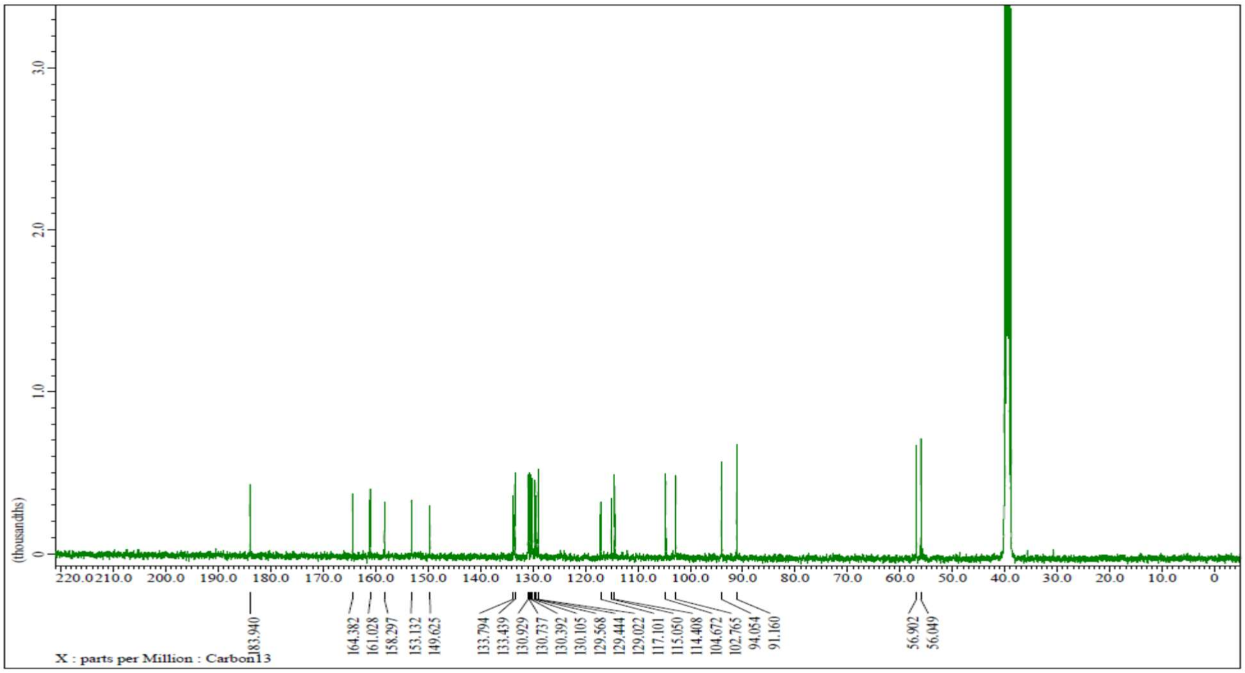

Mass Spectra

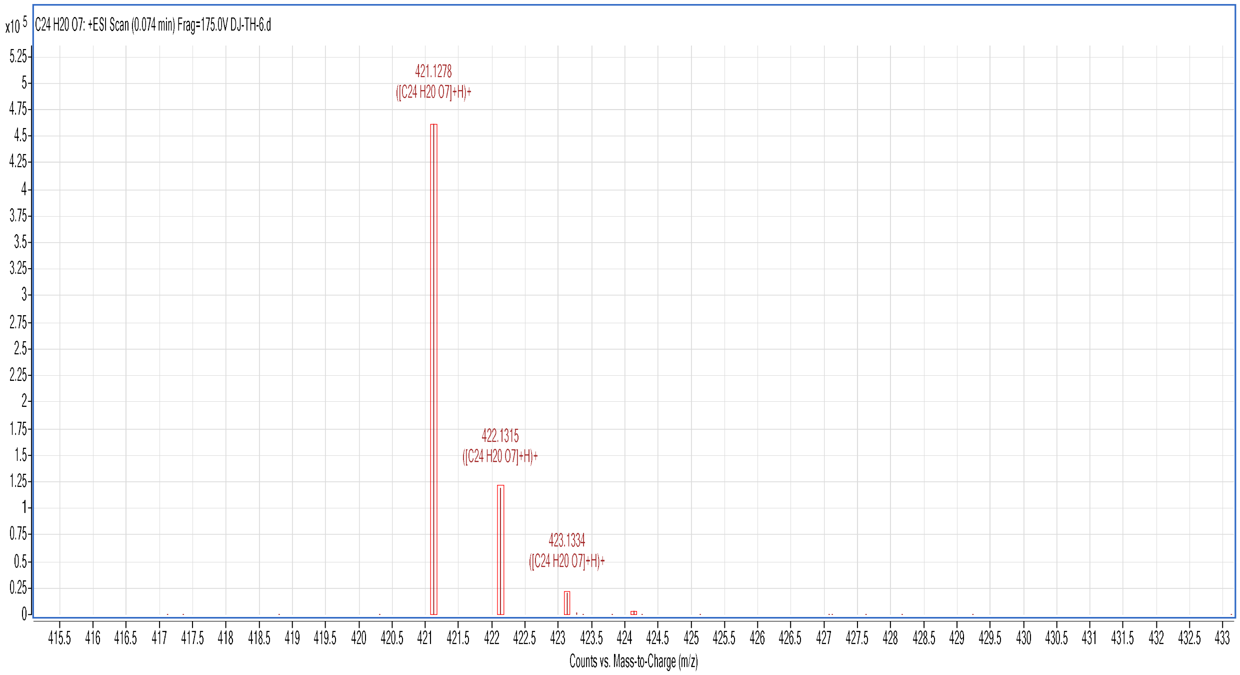

Figure S2: Structure, ^{1}H NMR, ^{13}C NMR and HRMS of Compound 2.

^{1}H NMR

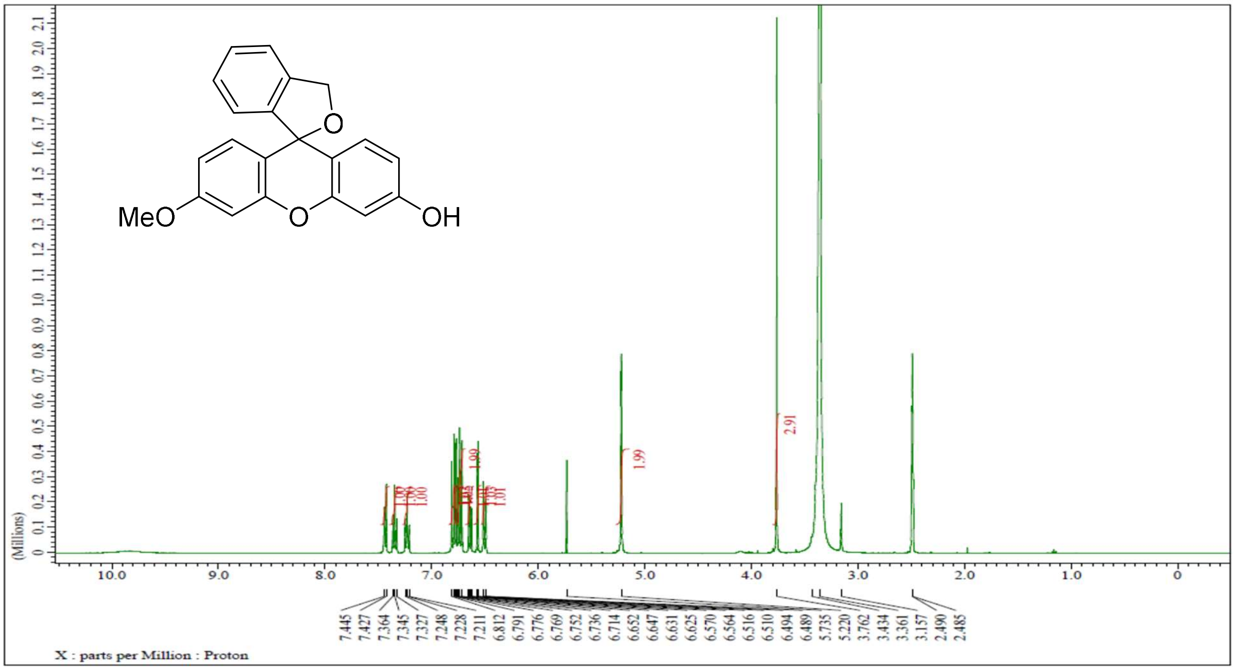

^{13}C NMR

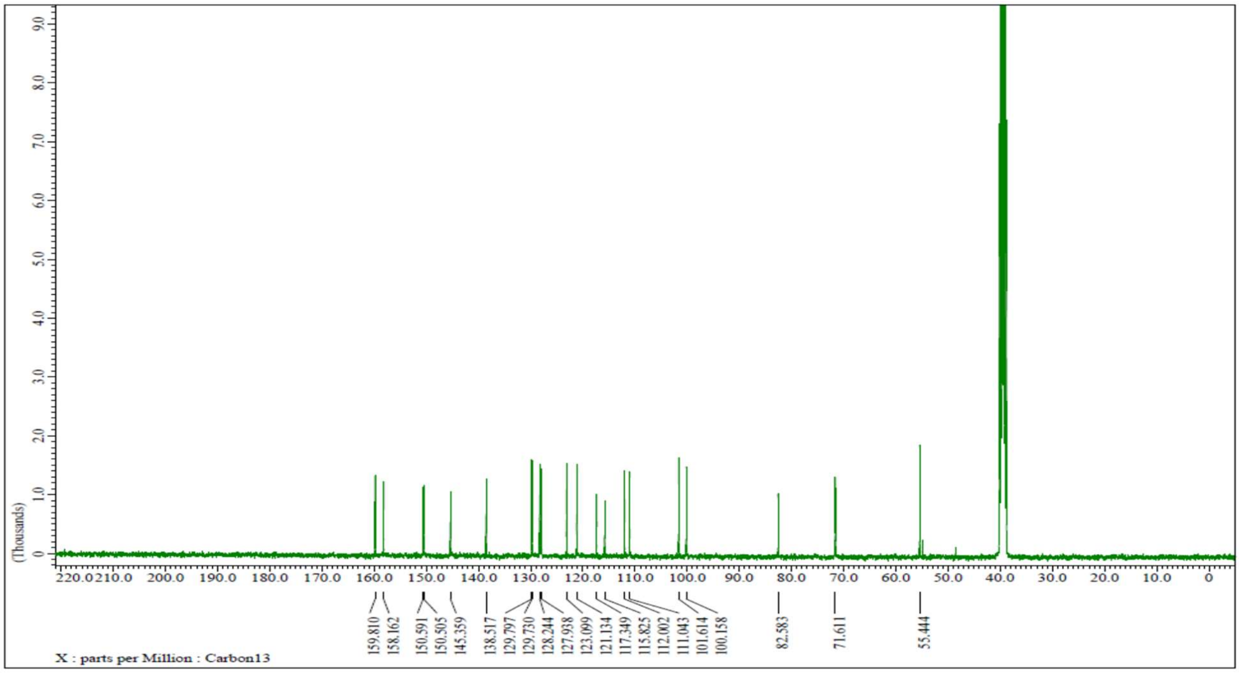

Mass Spectra

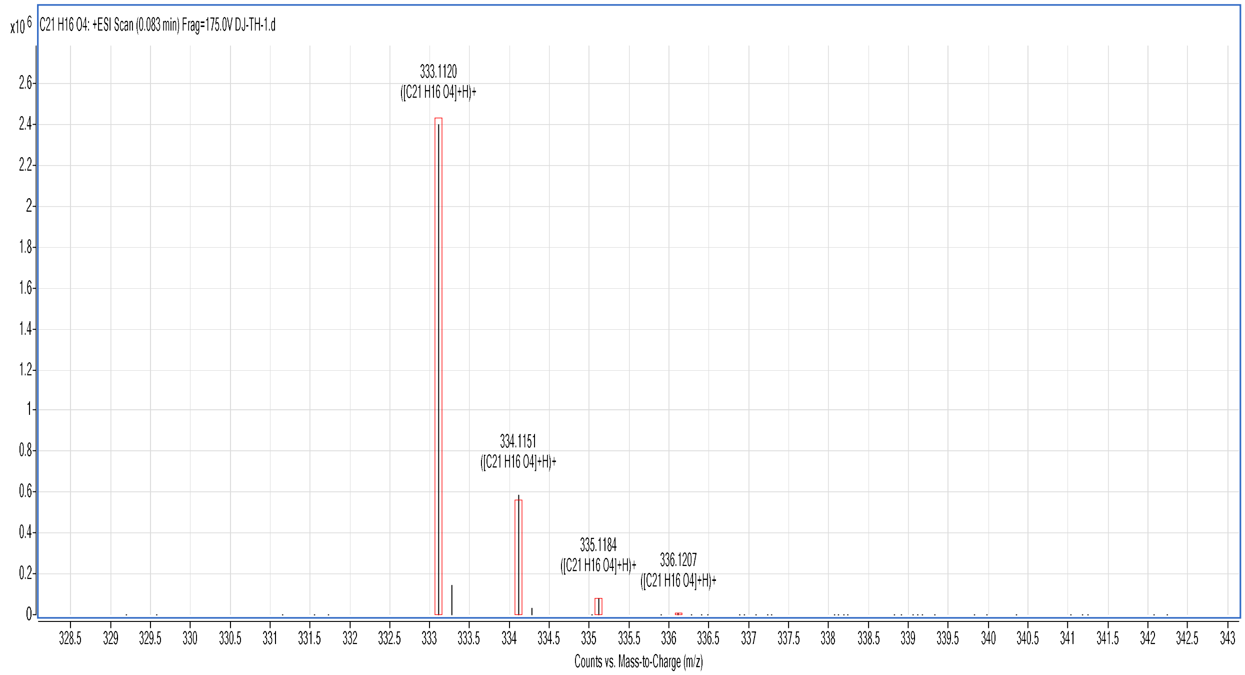

Figure S3: Structure, ^{1}H NMR, ^{13}C NMR and HRMS of Compound 3.

^{1}H NMR

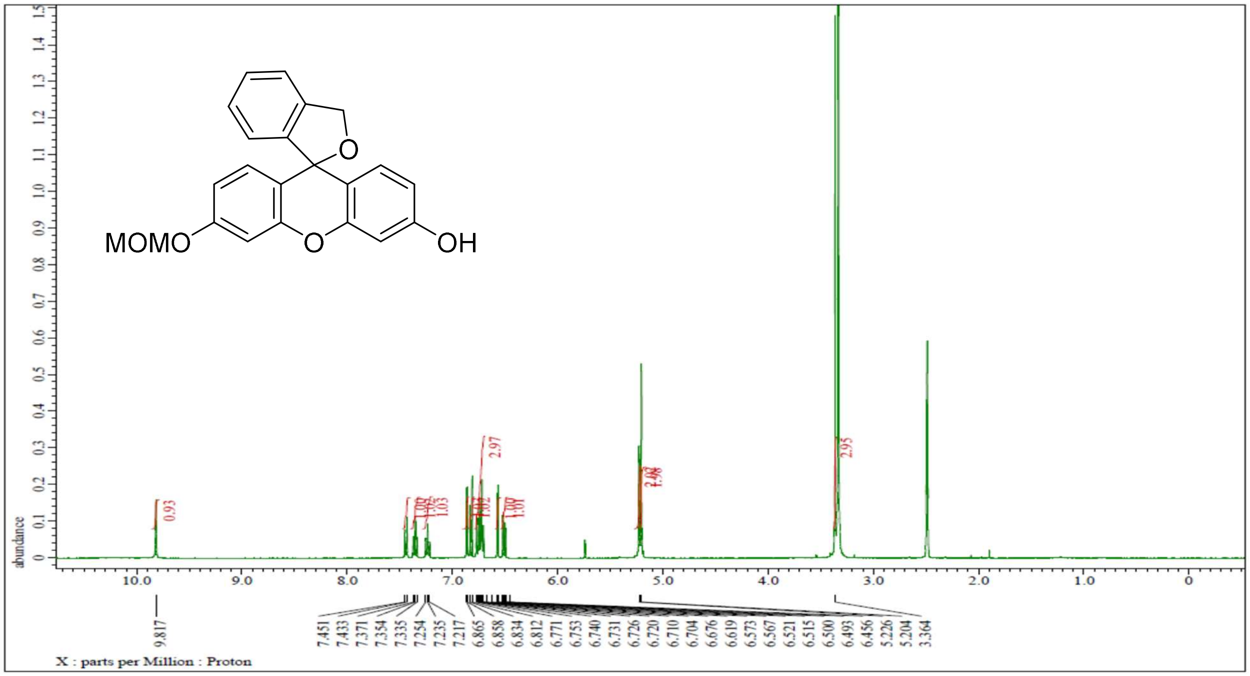

^{13}C NMR

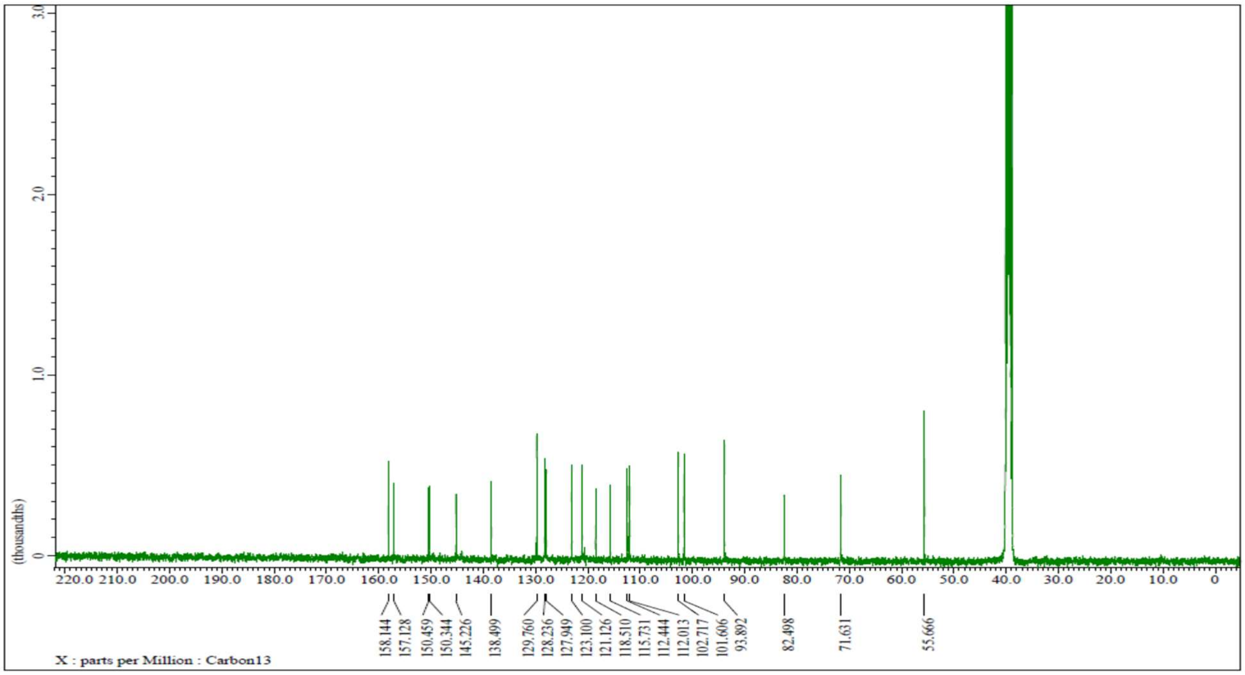

Mass Spectra

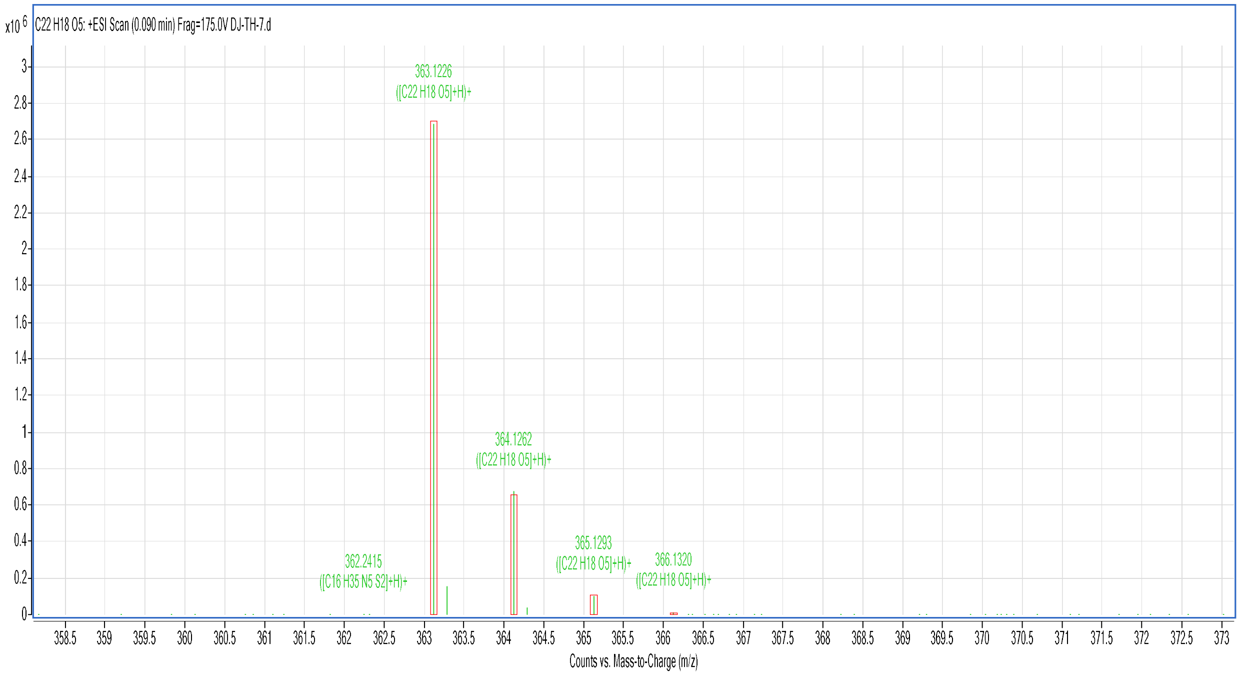

Figure S4: Structure, ^{1}H NMR, ^{13}C NMR and HRMS of Compound 4.

^{1}H NMR

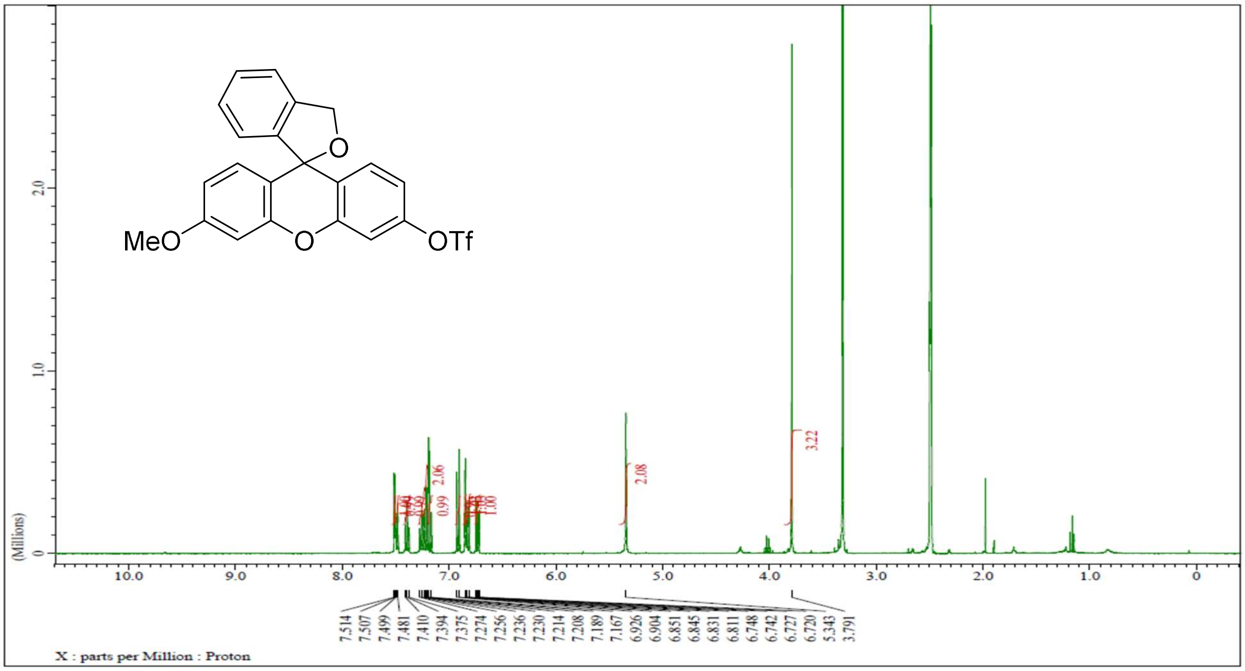

^{13}C NMR

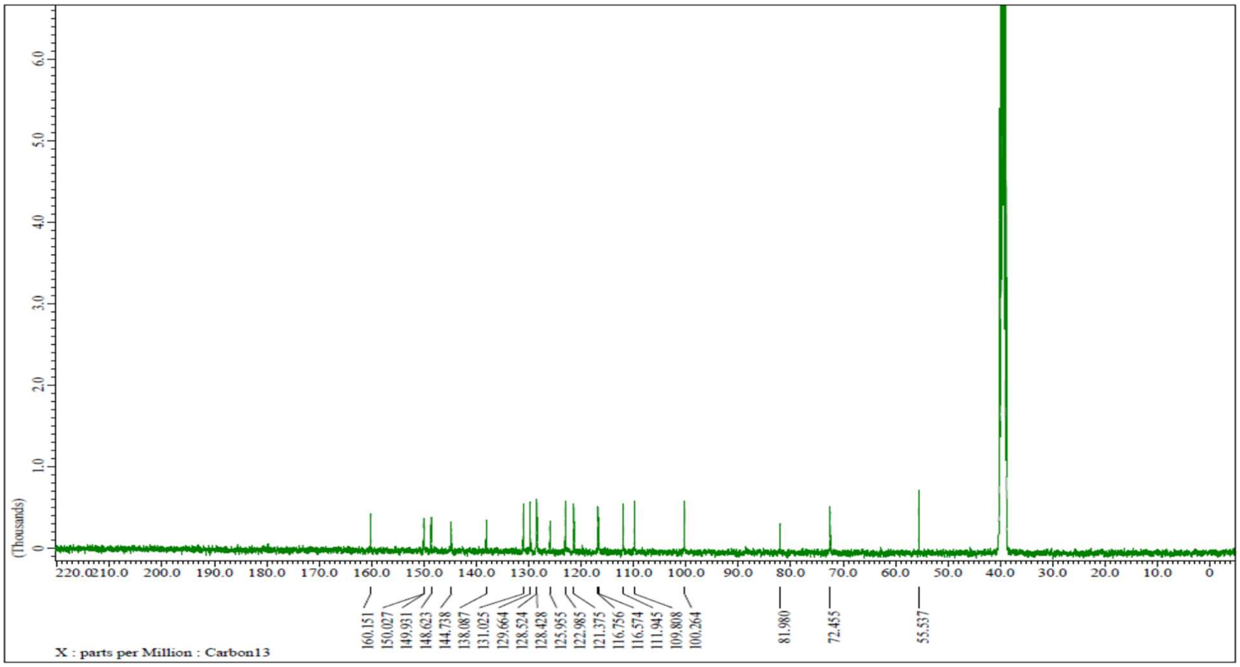

Mass Spectra

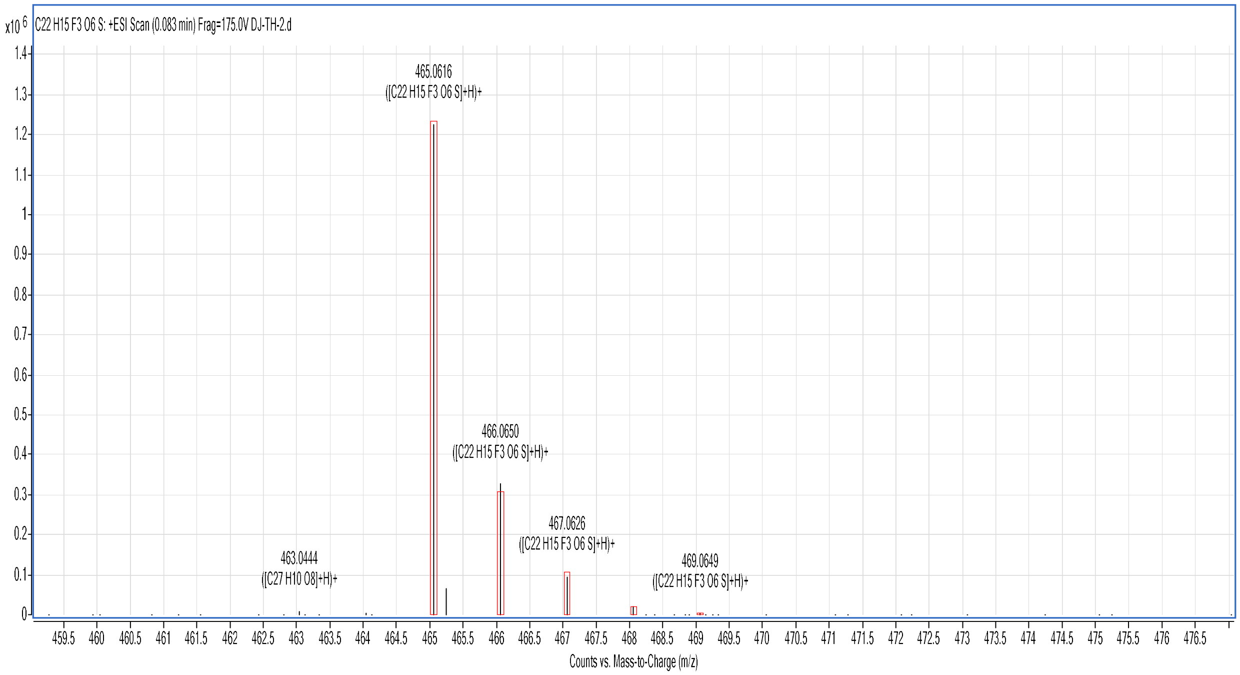

Figure S5: Structure, ^{1}H NMR, ^{13}C NMR and HRMS of Compound 5.

^{1}H NMR

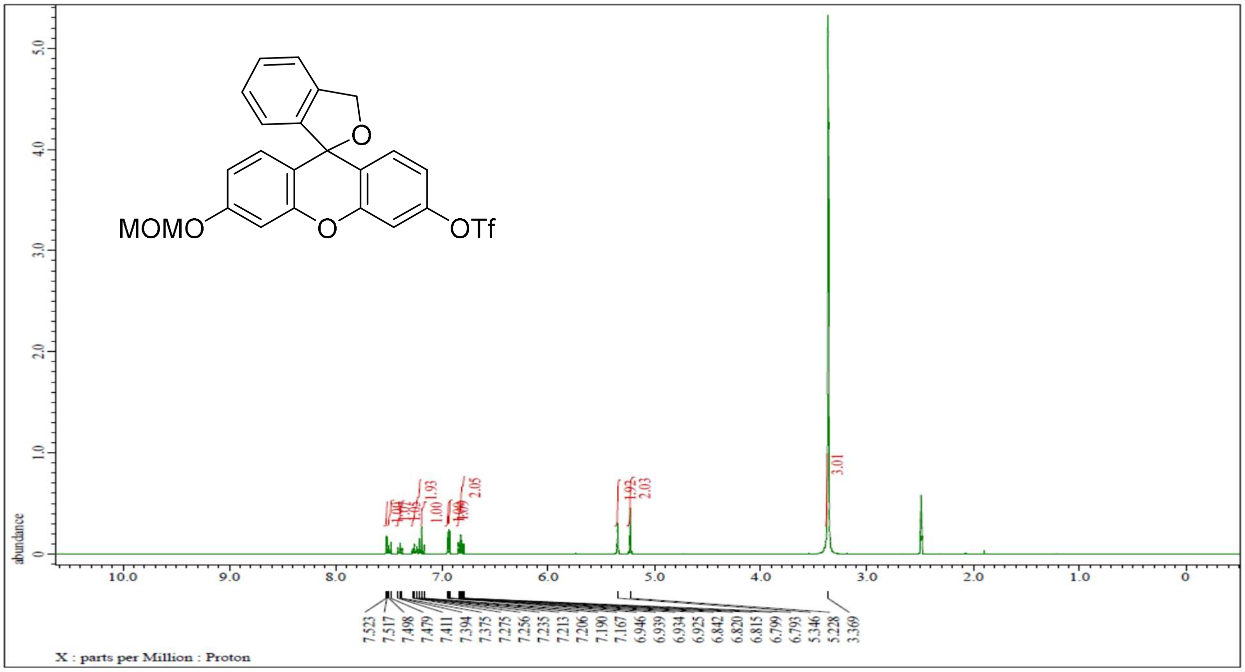

^{13}C NMR

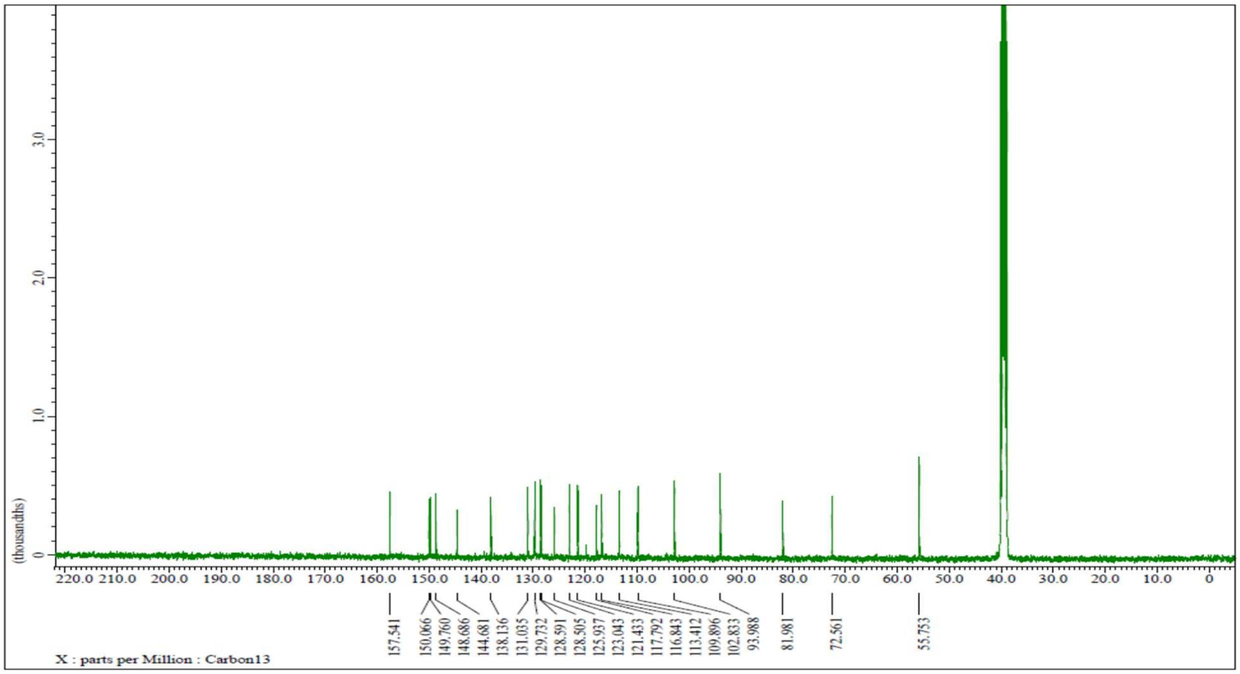

Mass Spectra

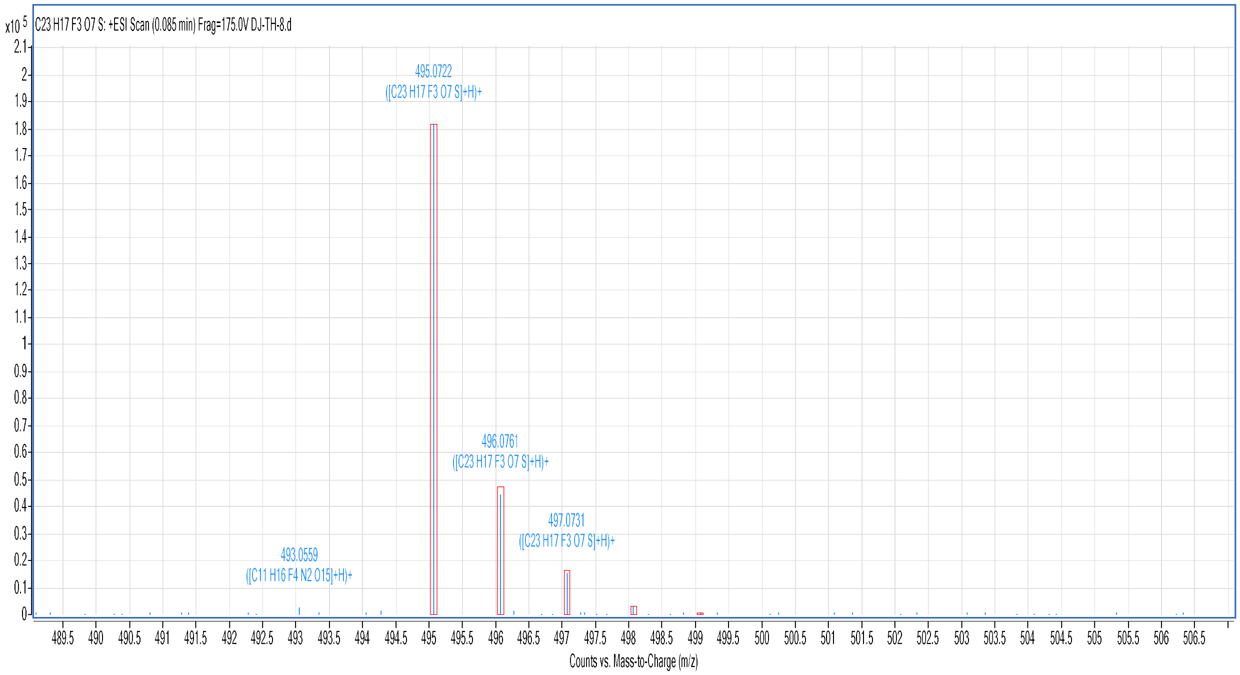

Figure S6: Structure, ^{1}H NMR, ^{13}C NMR and HRMS of Compound 6.

^{1}H NMR

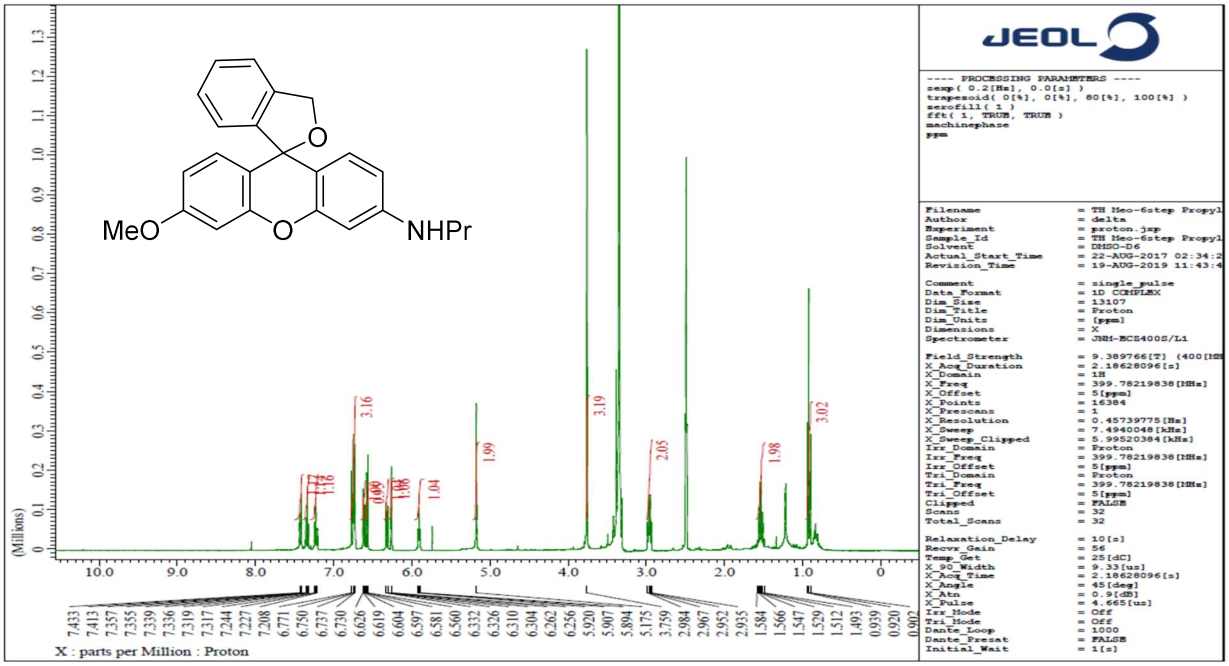

^{13}C NMR

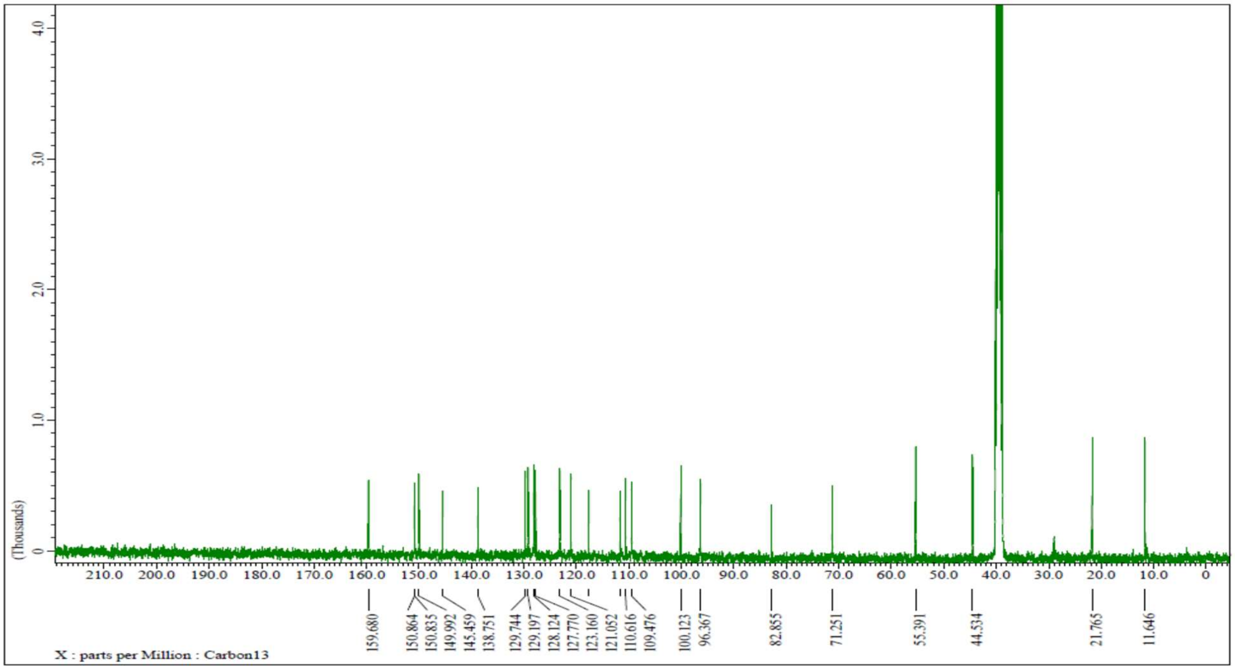

Mass Spectra

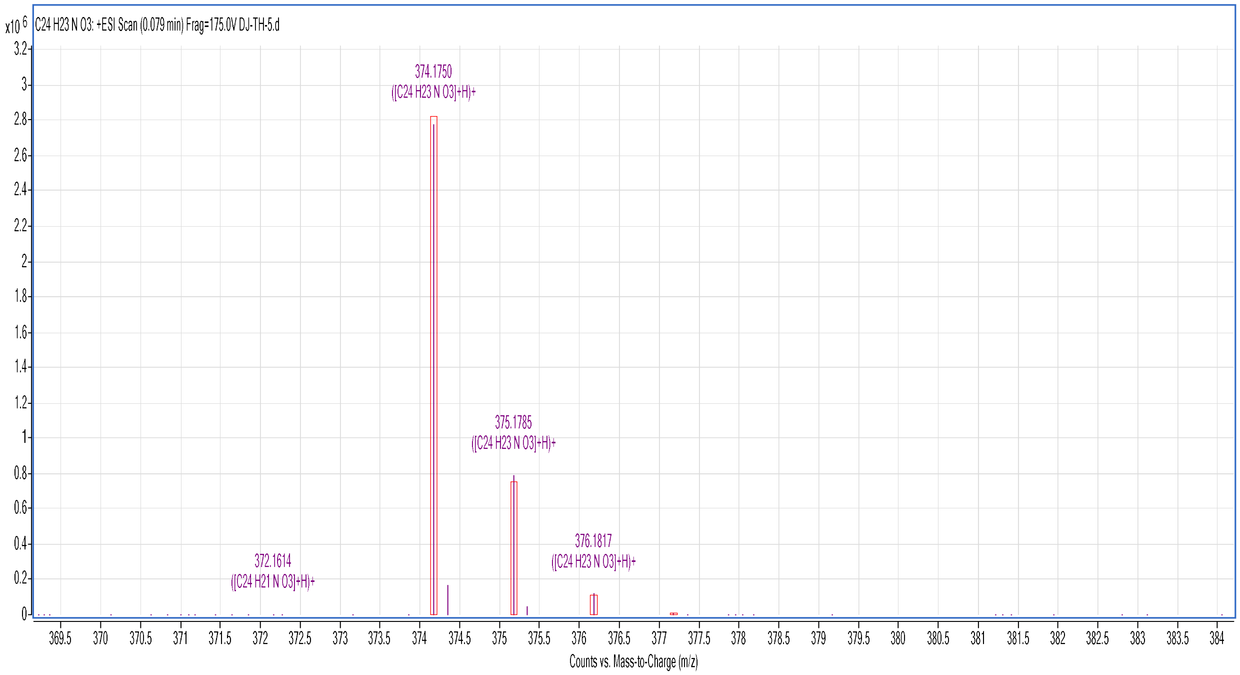

Figure S7: Structure, ^{1}H NMR, ^{13}C NMR and HRMS of Compound 7.

^{1}H NMR

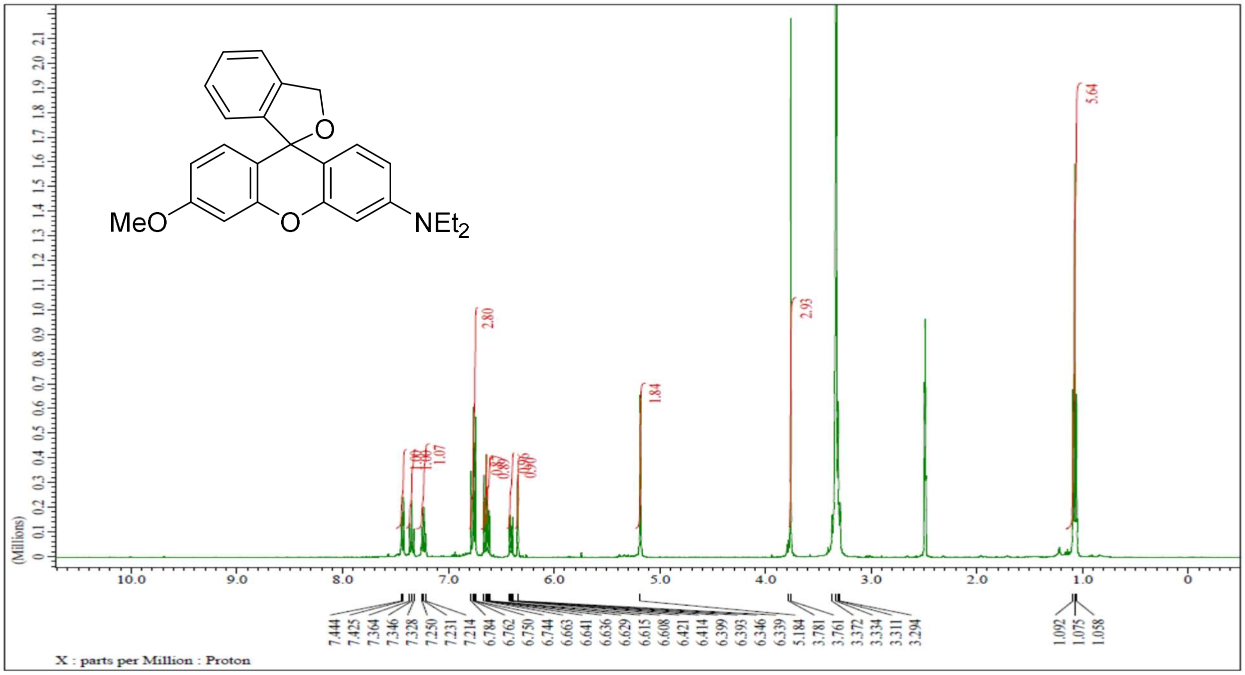

^{13}C NMR

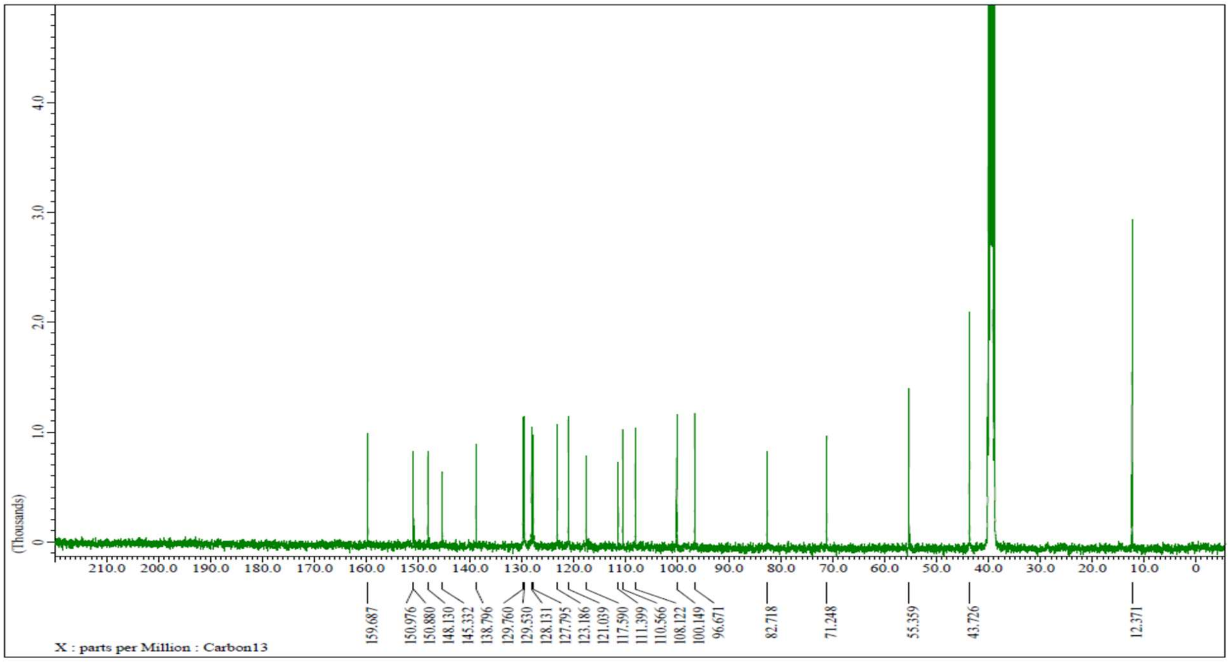

Mass Spectra

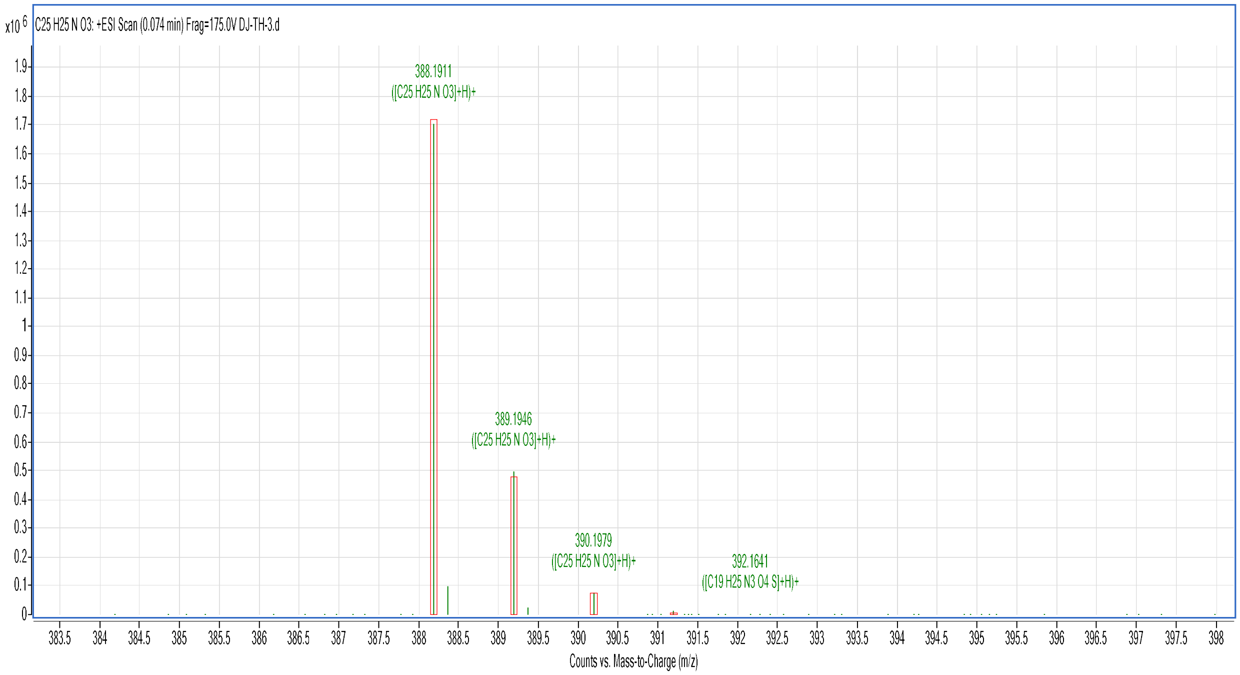

Figure S8: Structure, ^{1}H NMR, ^{13}C NMR and HRMS of Compound 8.

^{1}H NMR

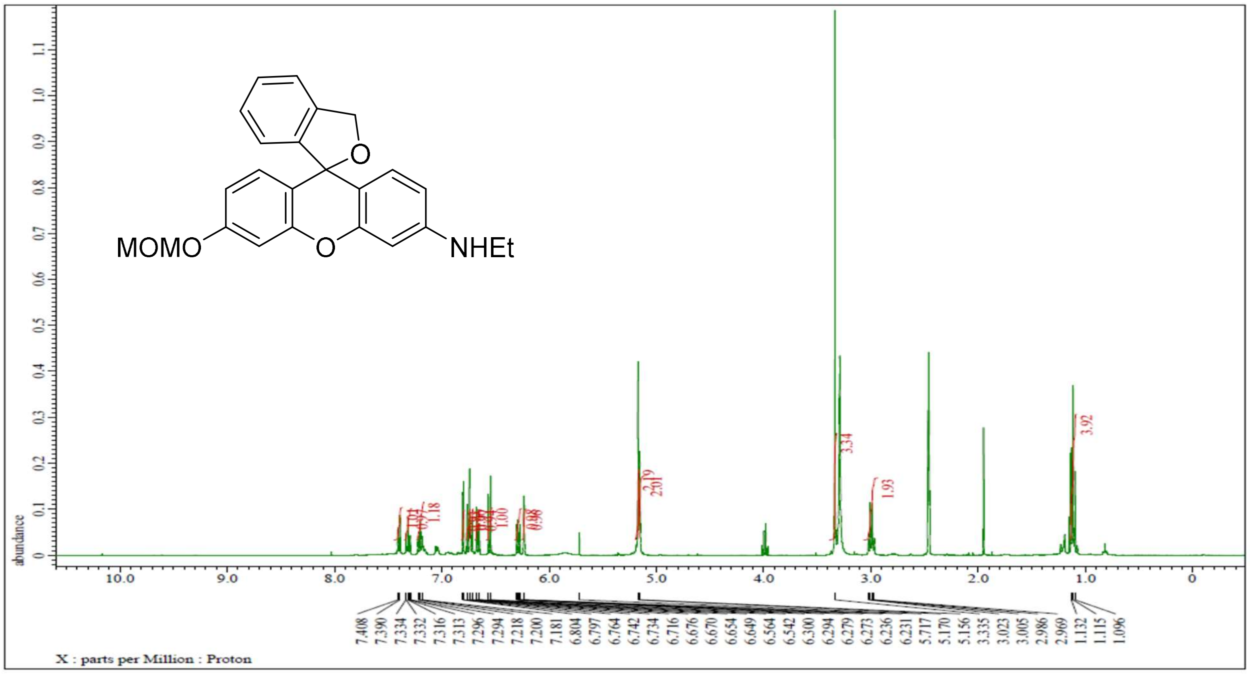

^{13}C NMR

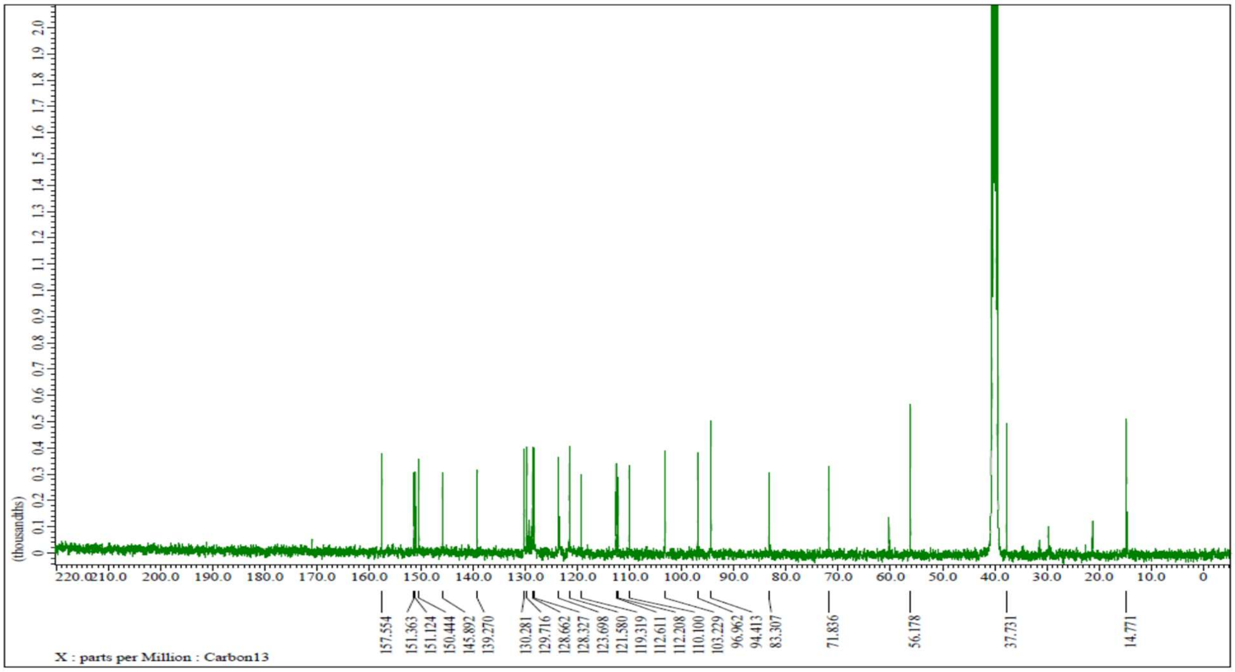

Mass Spectra

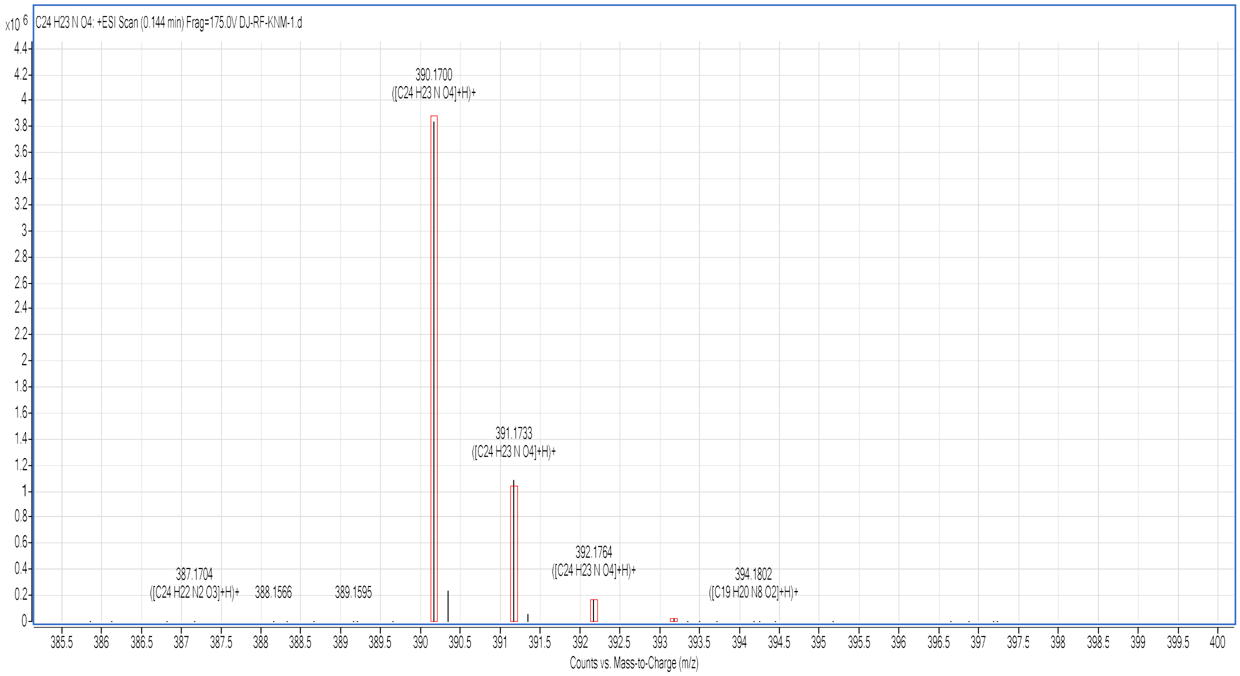

Figure S9: Structure, ^{1}H NMR, ^{13}C NMR and HRMS of Compound 10.

^{1}H NMR

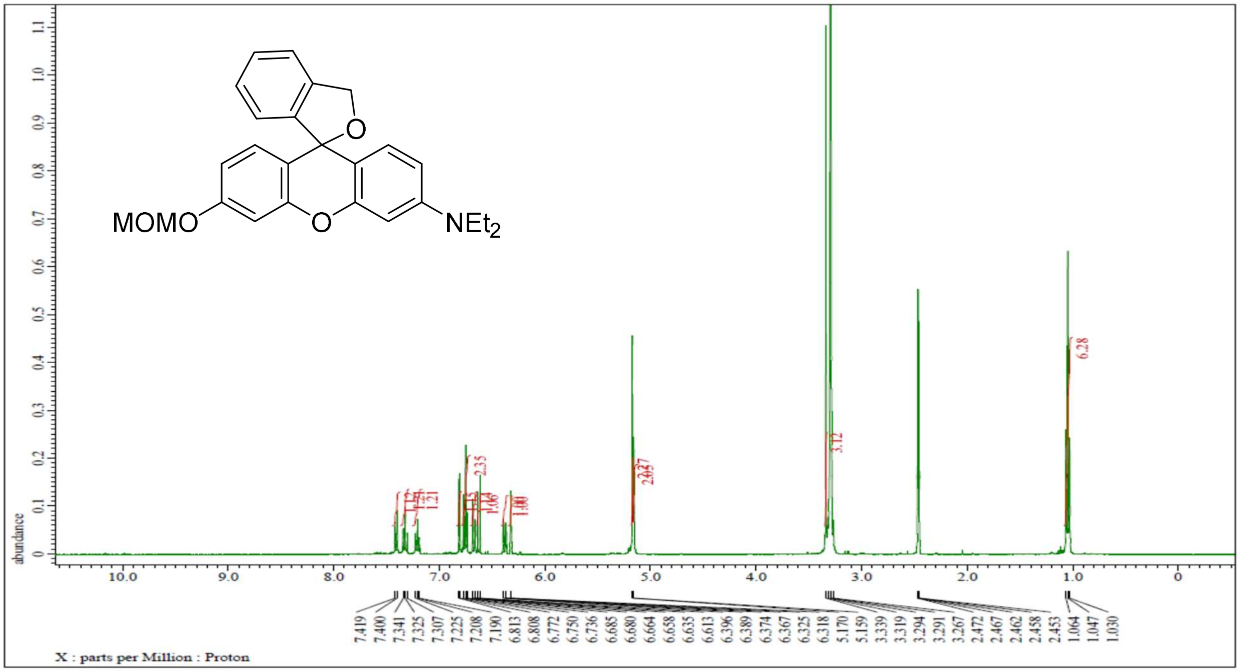

^{13}C NMR

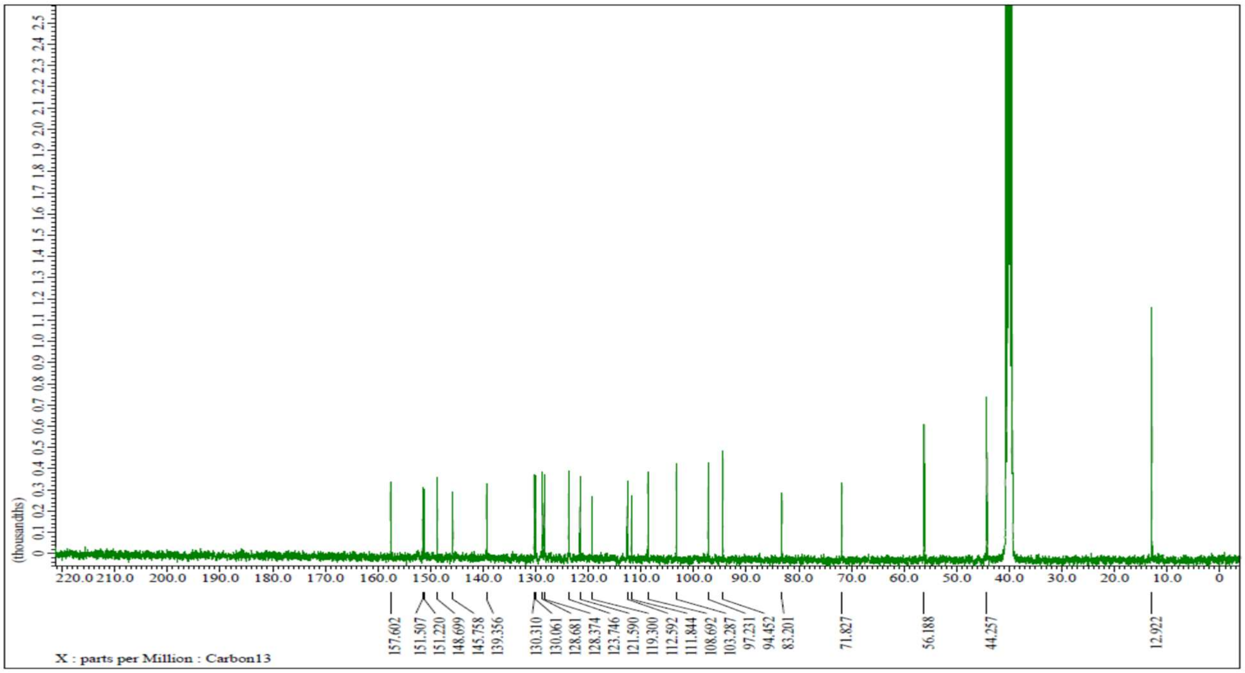

Mass Spectra

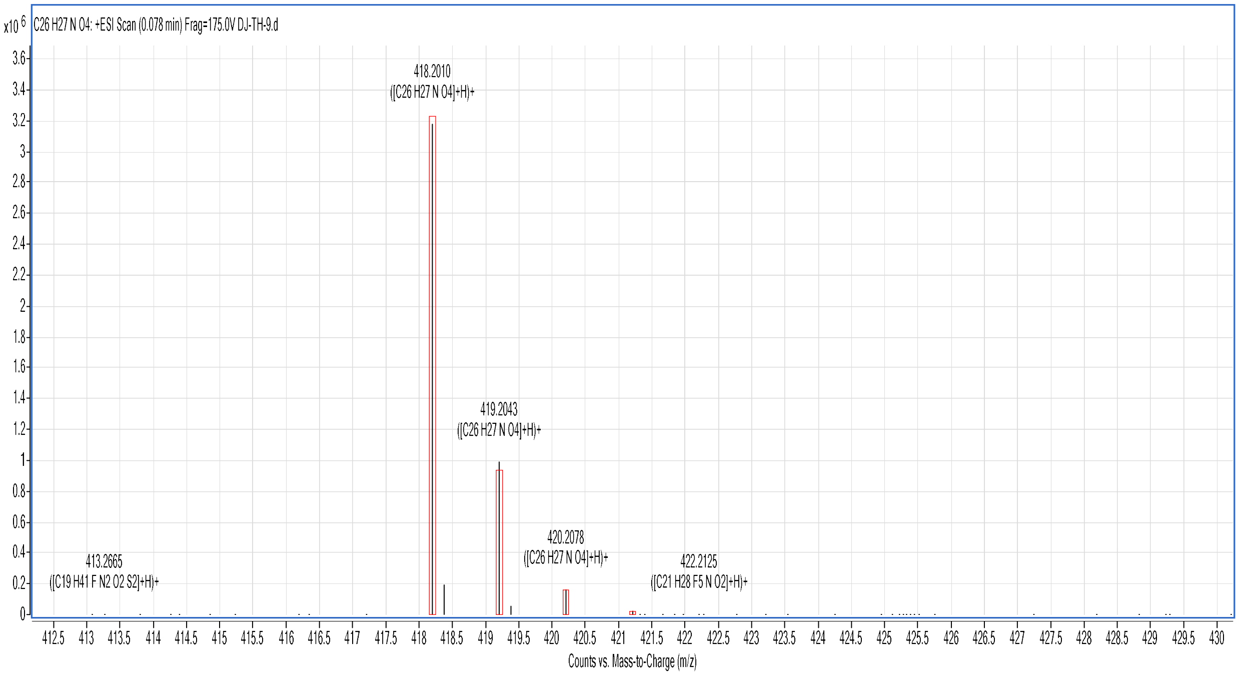

Figure S10: Structure, ^{1}H NMR, ^{13}C NMR and HRMS of Compound 11.

^1H NMR

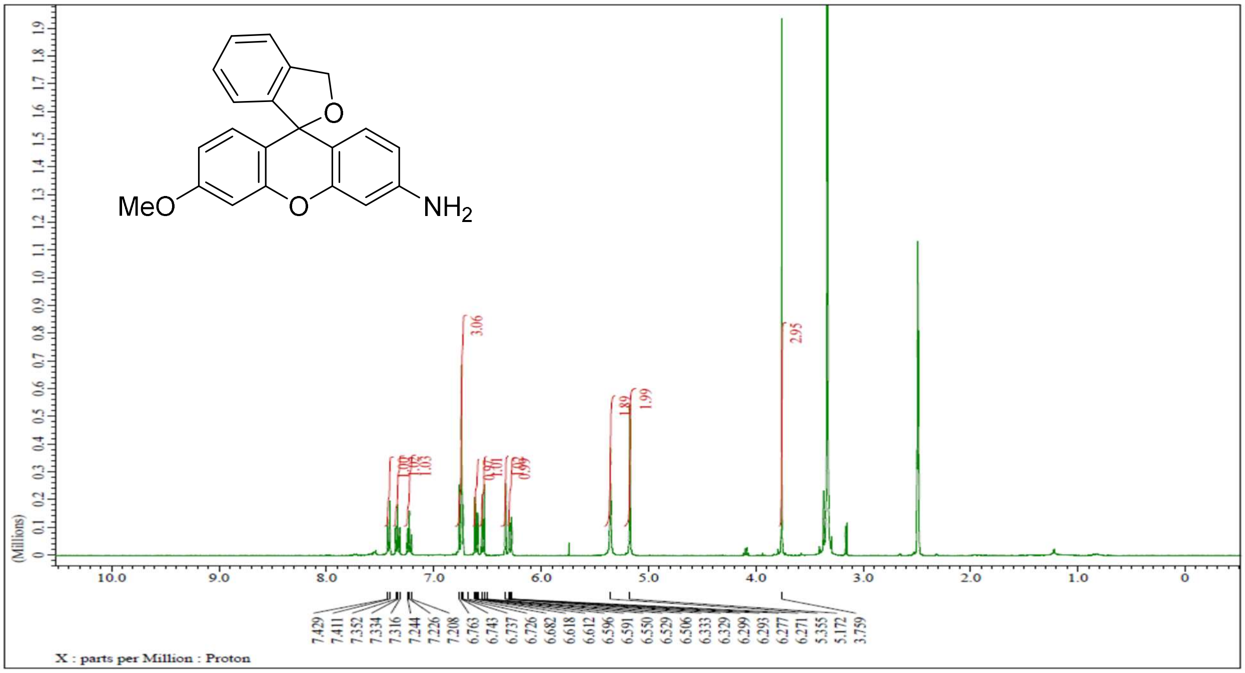

^{13}C NMR

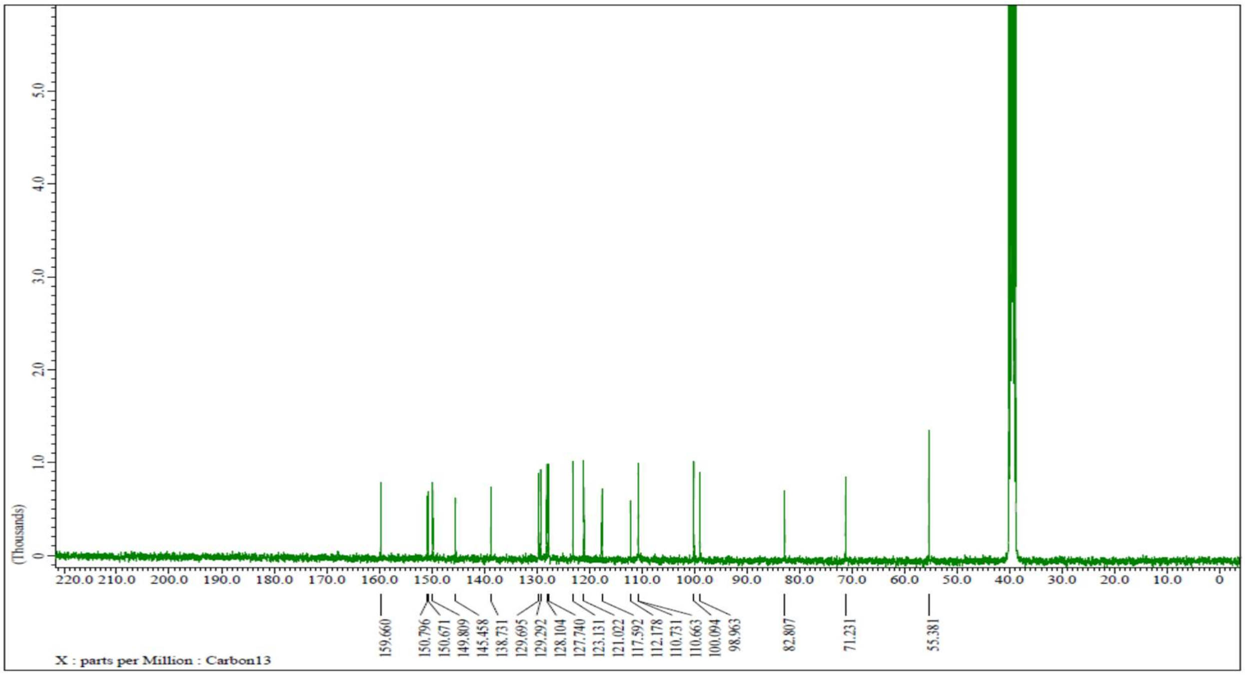

Mass Spectra

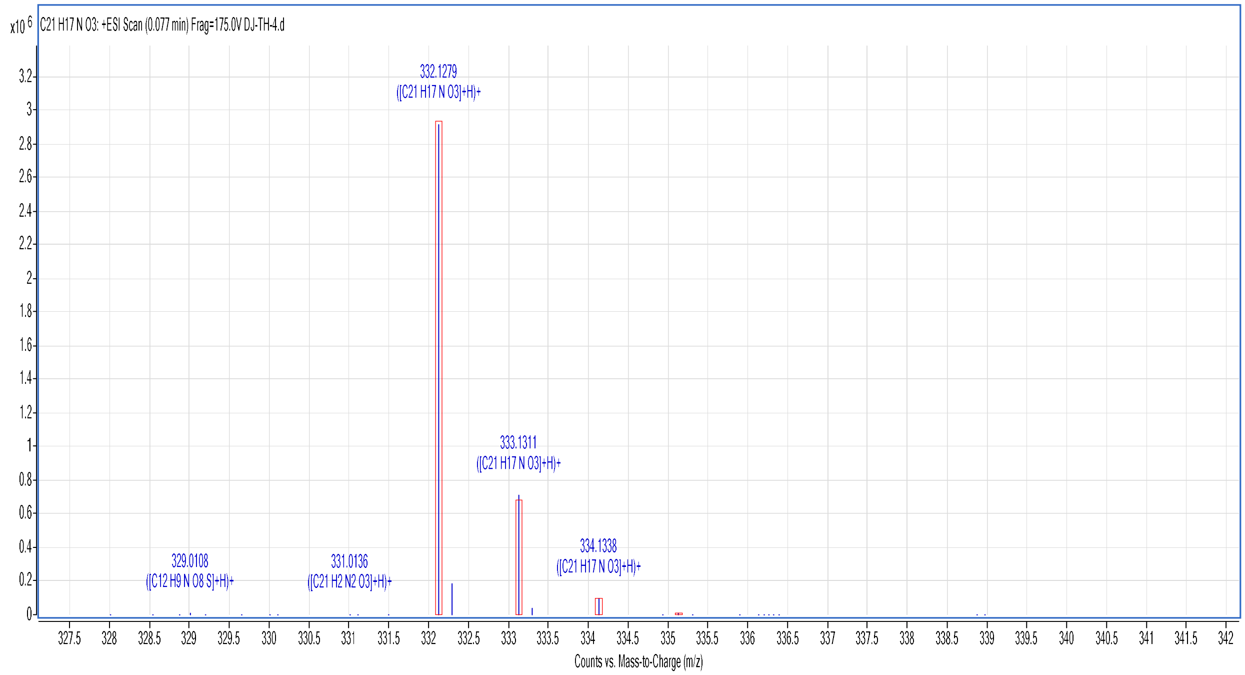

Figure S11: Structure, ^{1}H NMR, ^{13}C NMR and HRMS of Compound 12.

^{1}H NMR

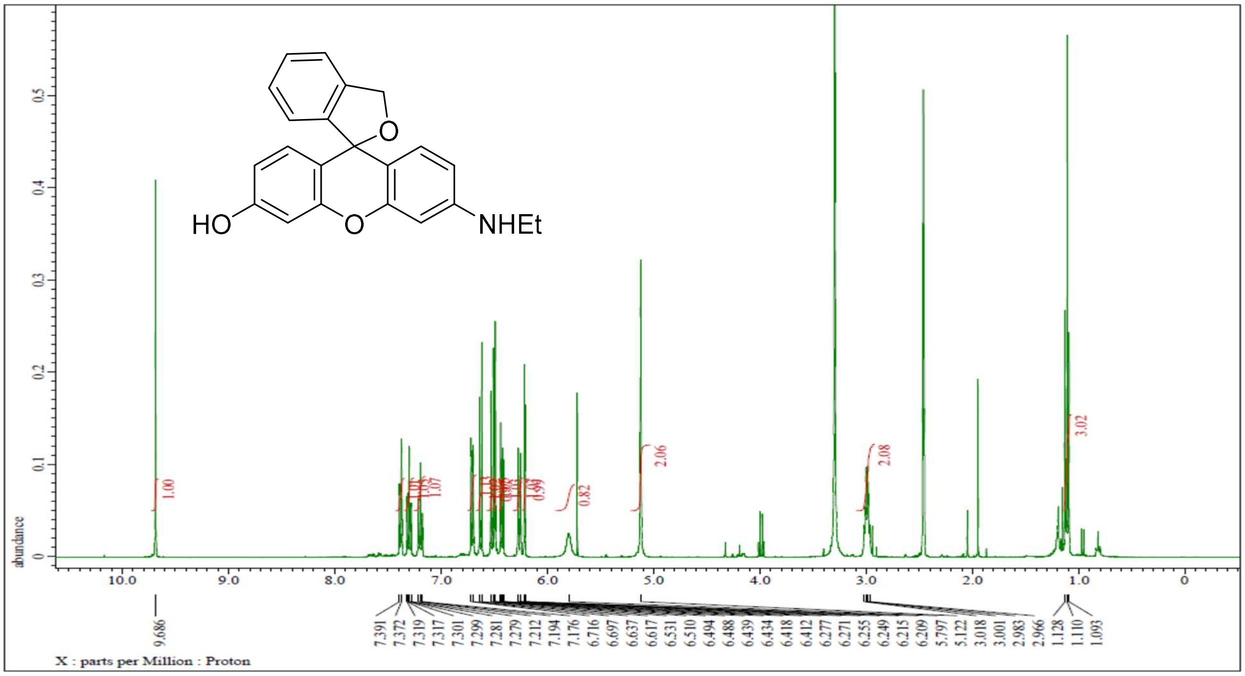

^{13}C NMR

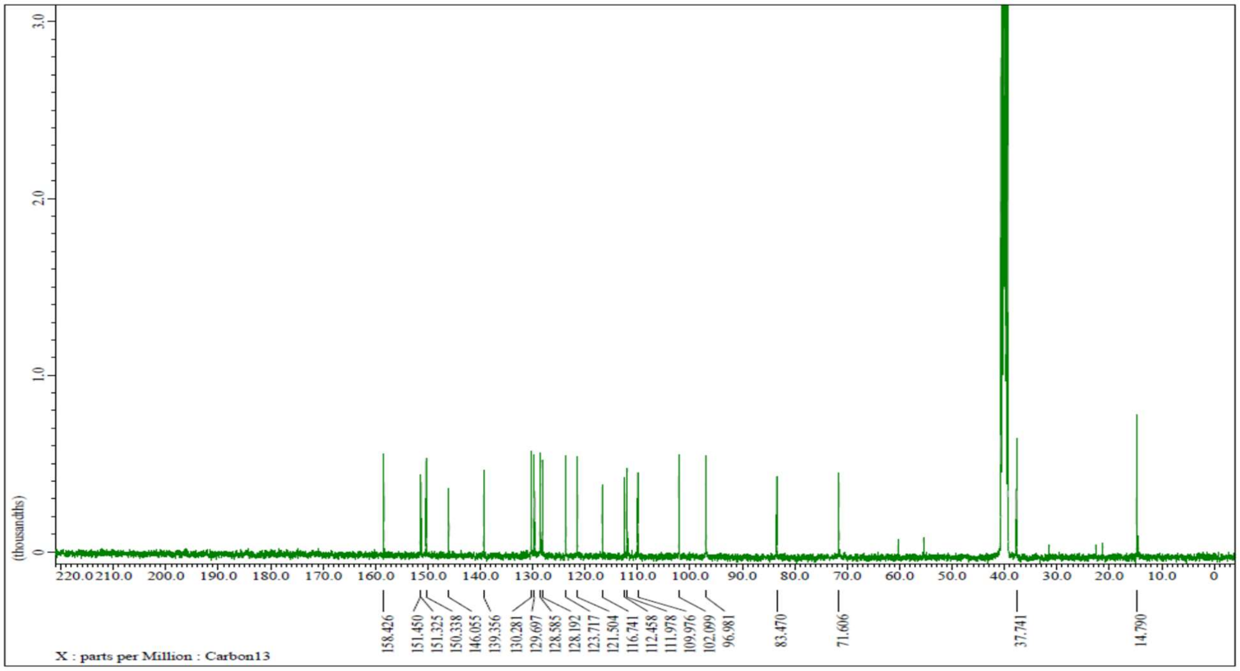

Mass Spectra

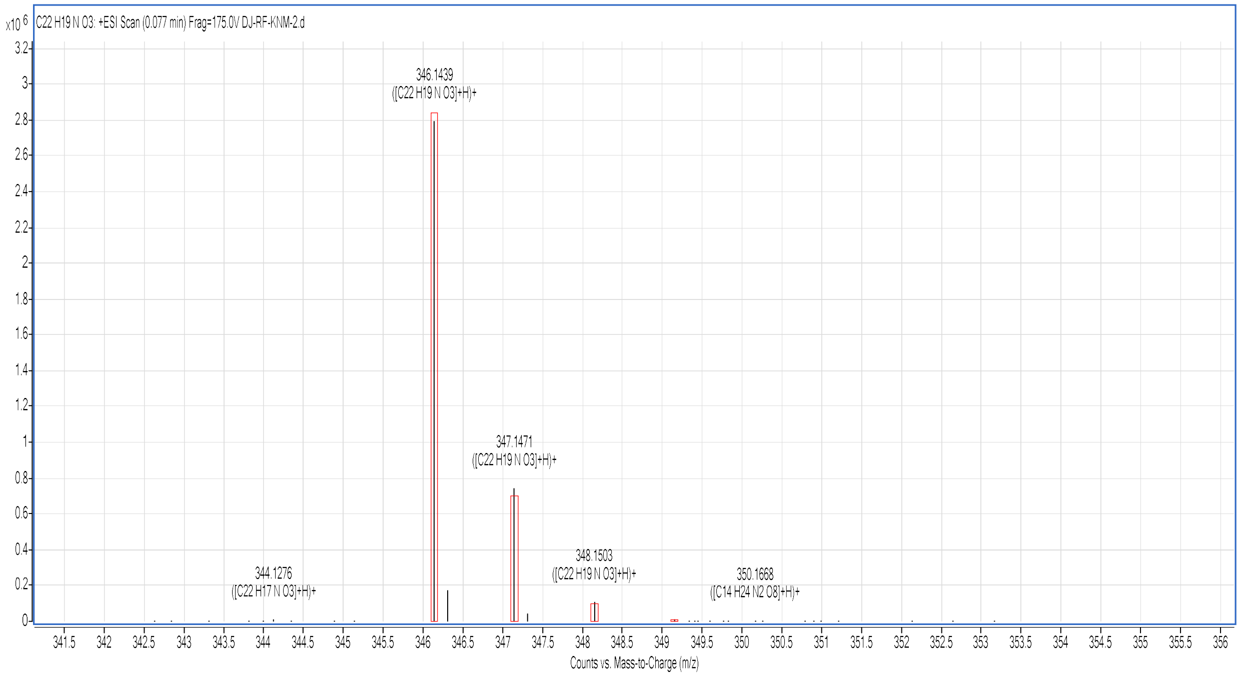

Figure S12: Structure, ^{1}H NMR, ^{13}C NMR and HRMS of Compound 13.

^{1}H NMR

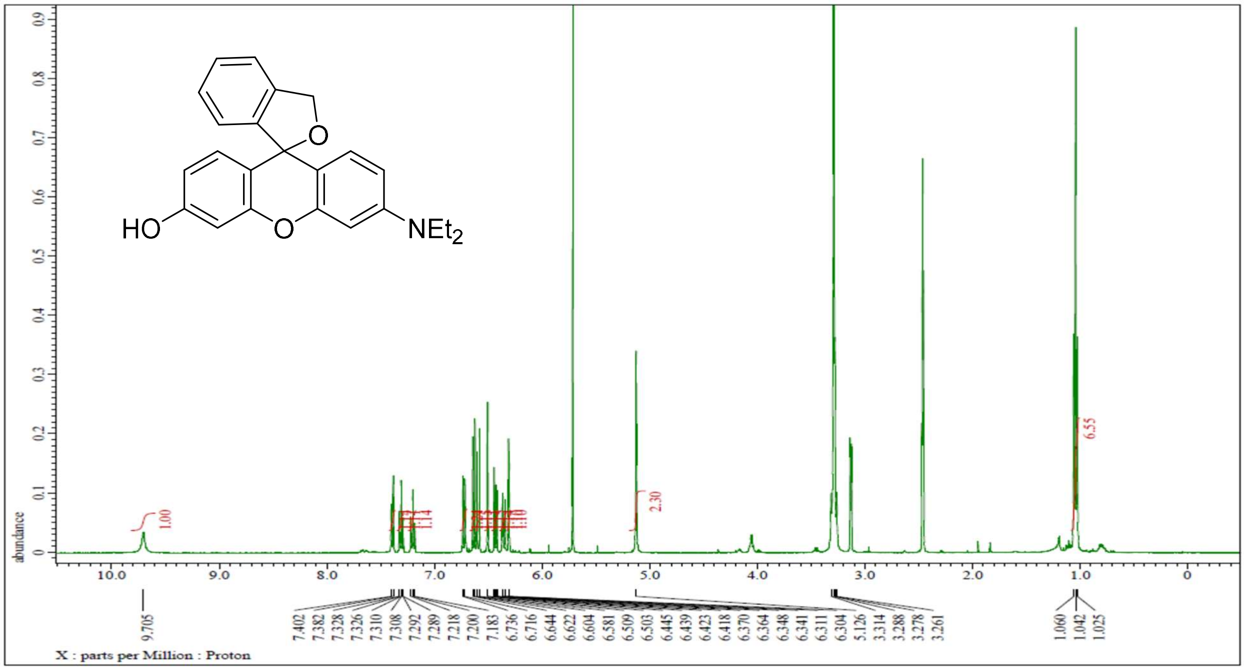

^{13}C NMR

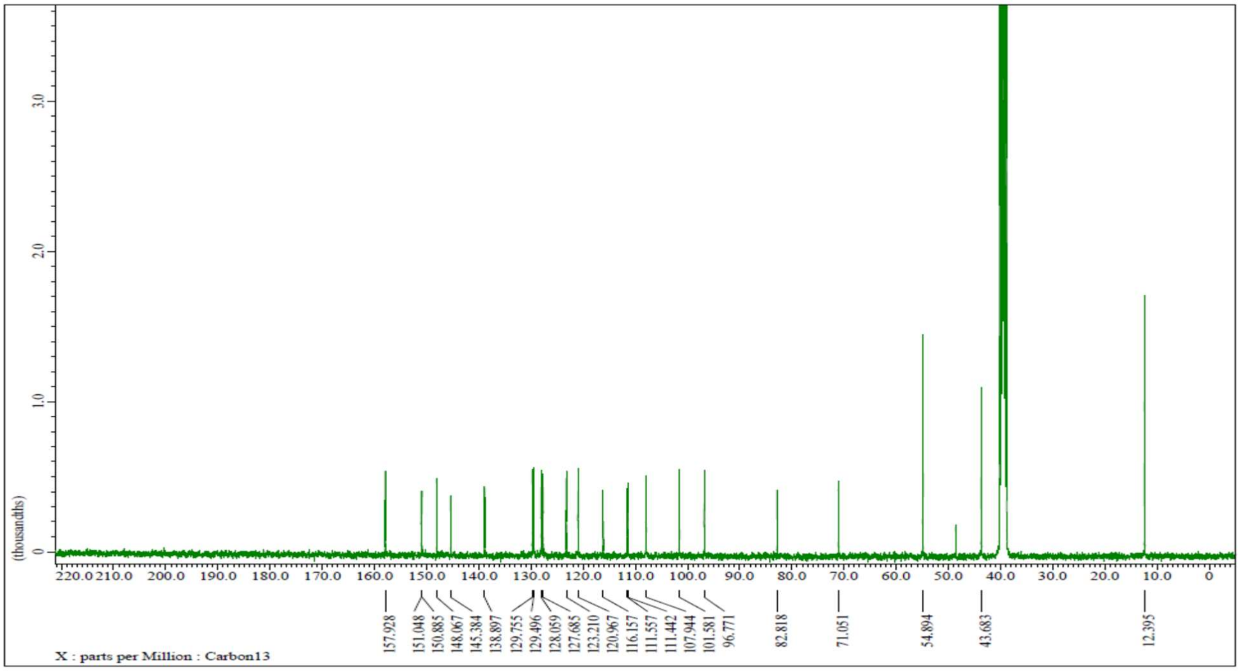

Mass Spectra

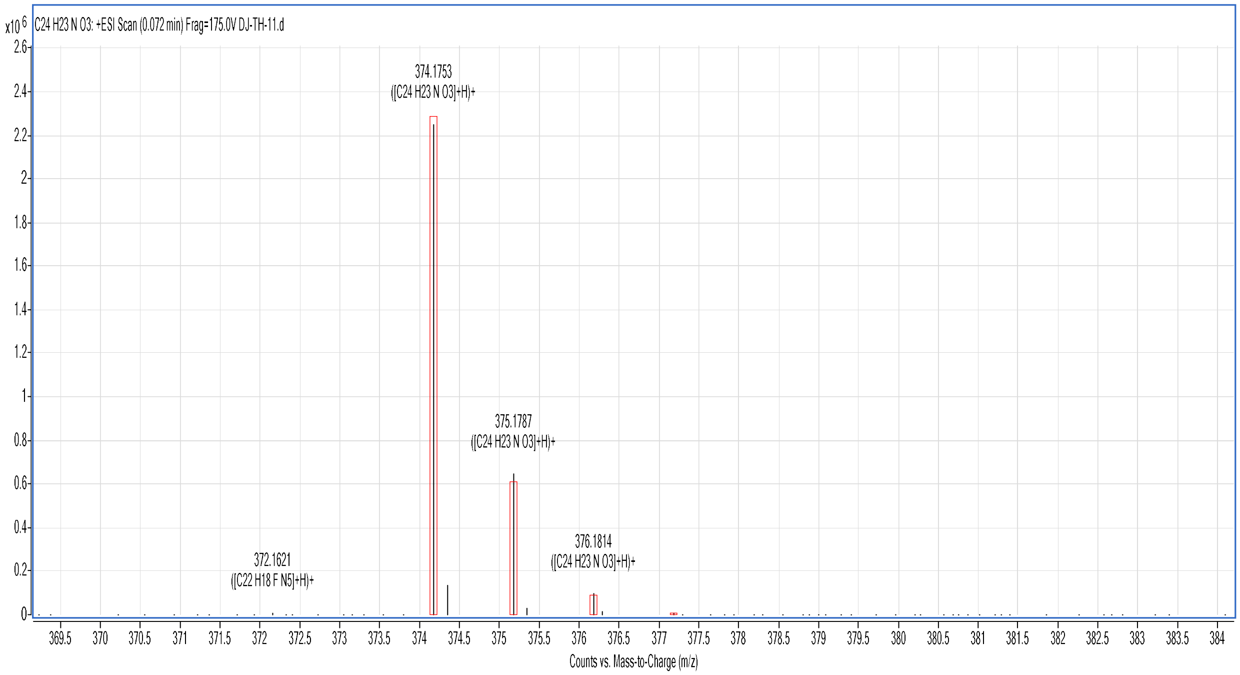

Figure S13: Structure, ^{1}H NMR, ^{13}C NMR and HRMS of Compound 14.

^{1}H NMR

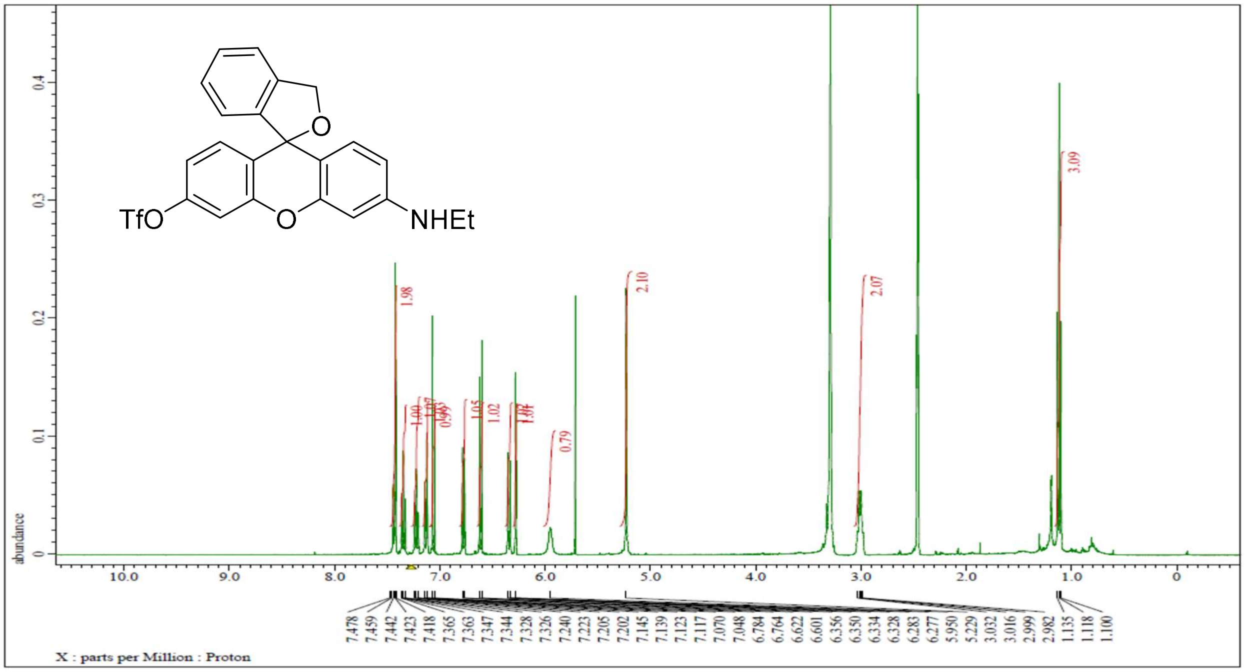

^{13}C NMR

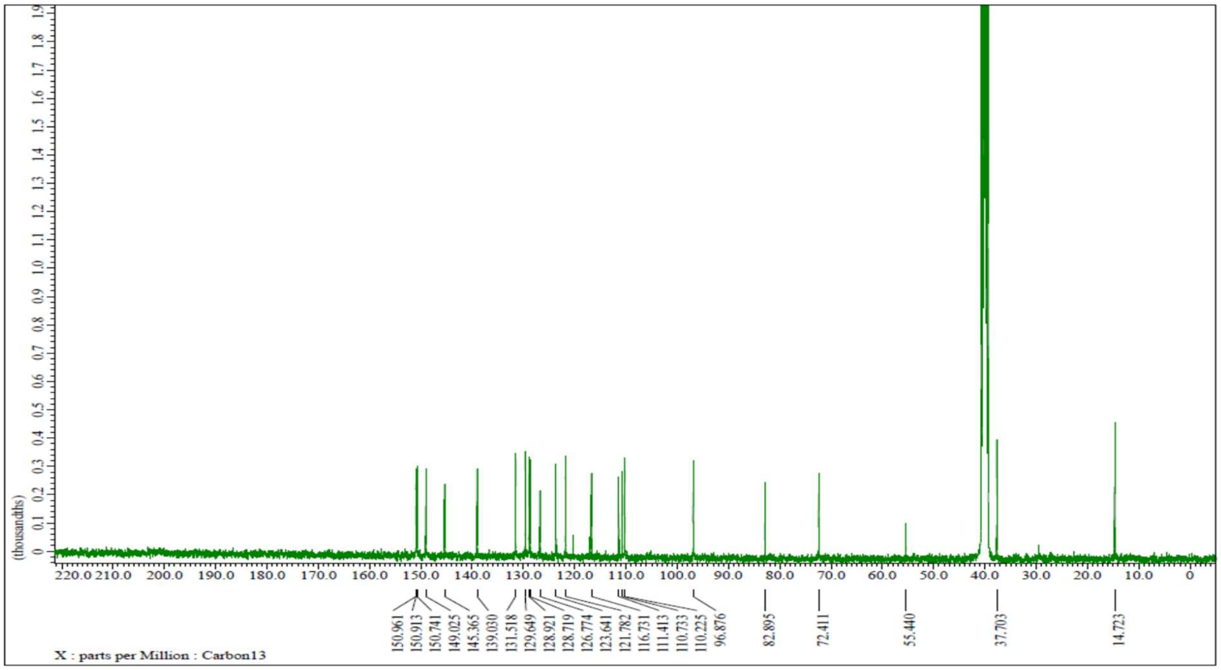

Mass Spectra

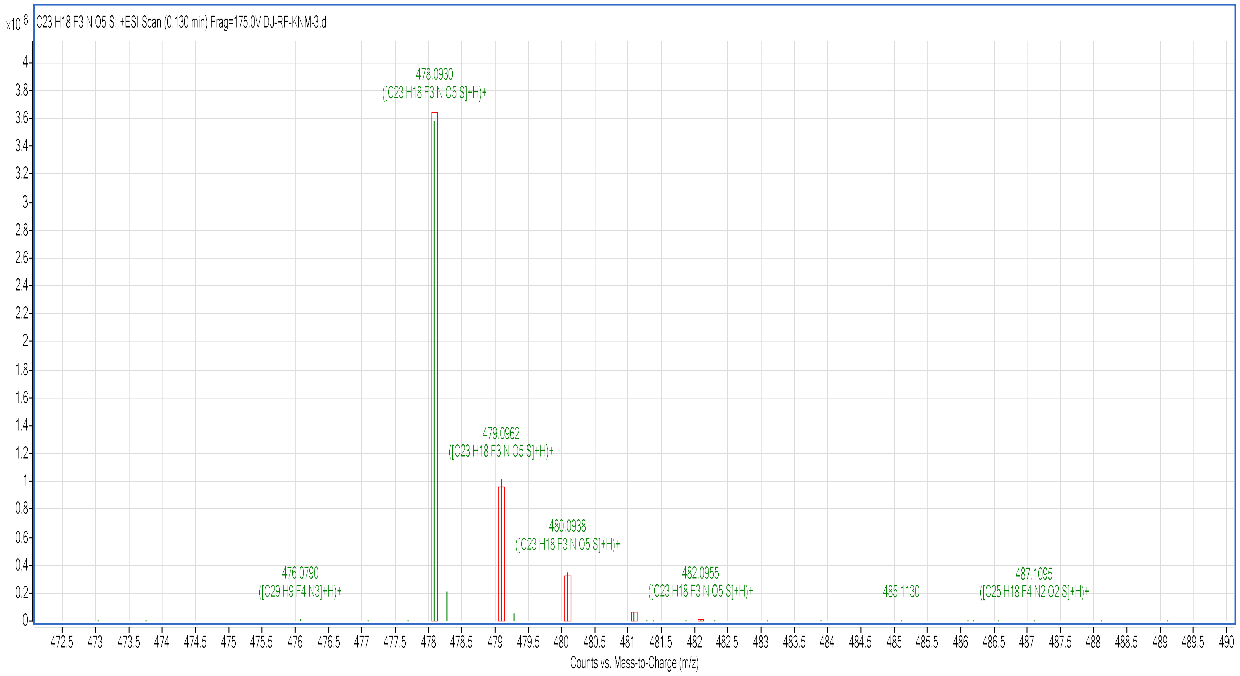

Figure S14: Structure, ^{1}H NMR, ^{13}C NMR and HRMS of Compound 15.

^{1}H NMR

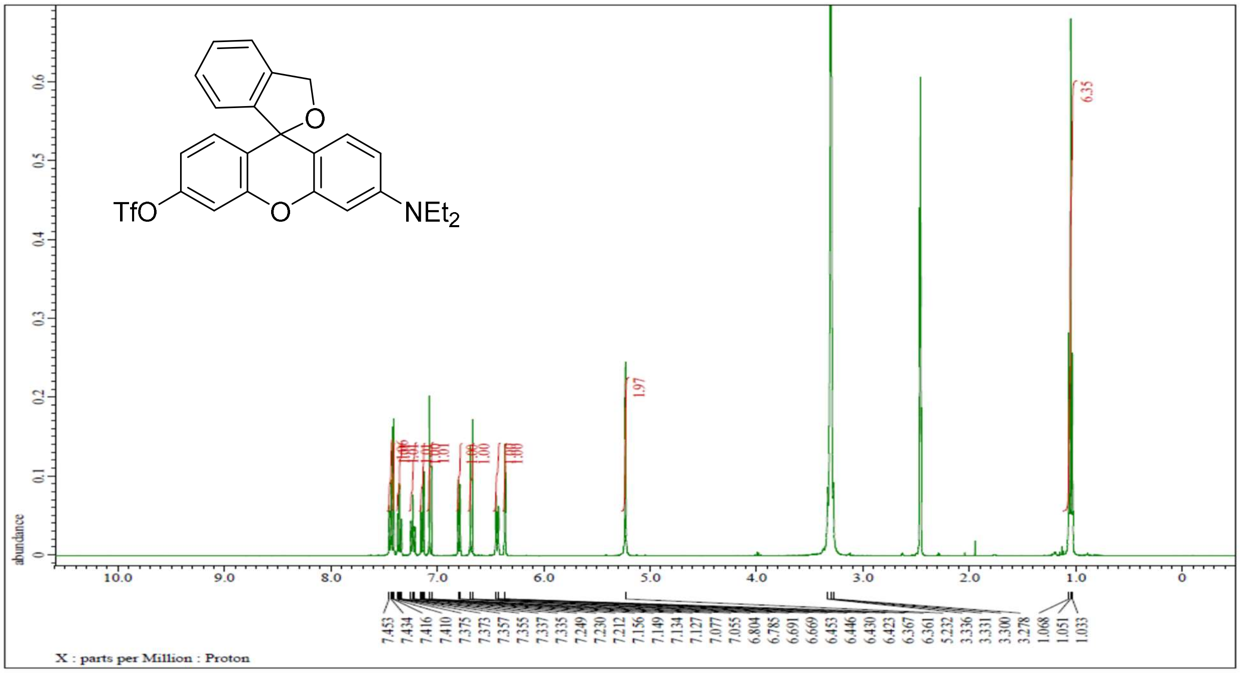

^{13}C NMR

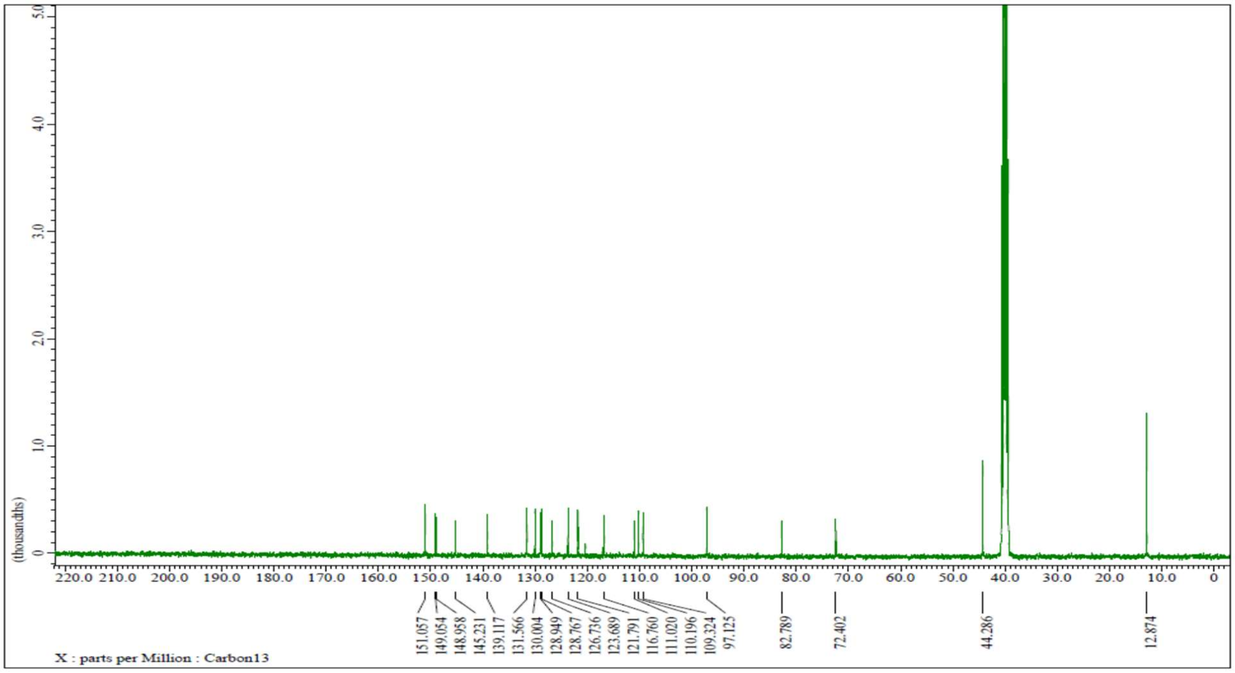

Mass Spectra

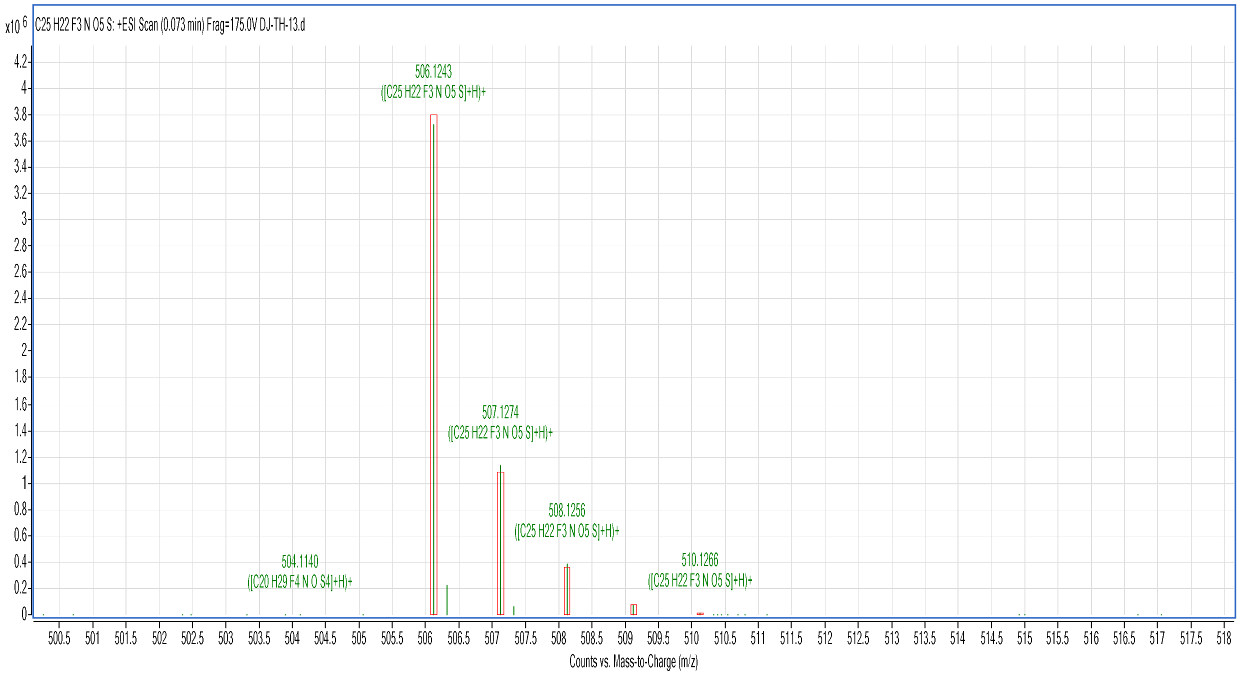

Figure S15: Structure, ^{1}H NMR, ^{13}C NMR and HRMS of Compound 16.

^{1}H NMR

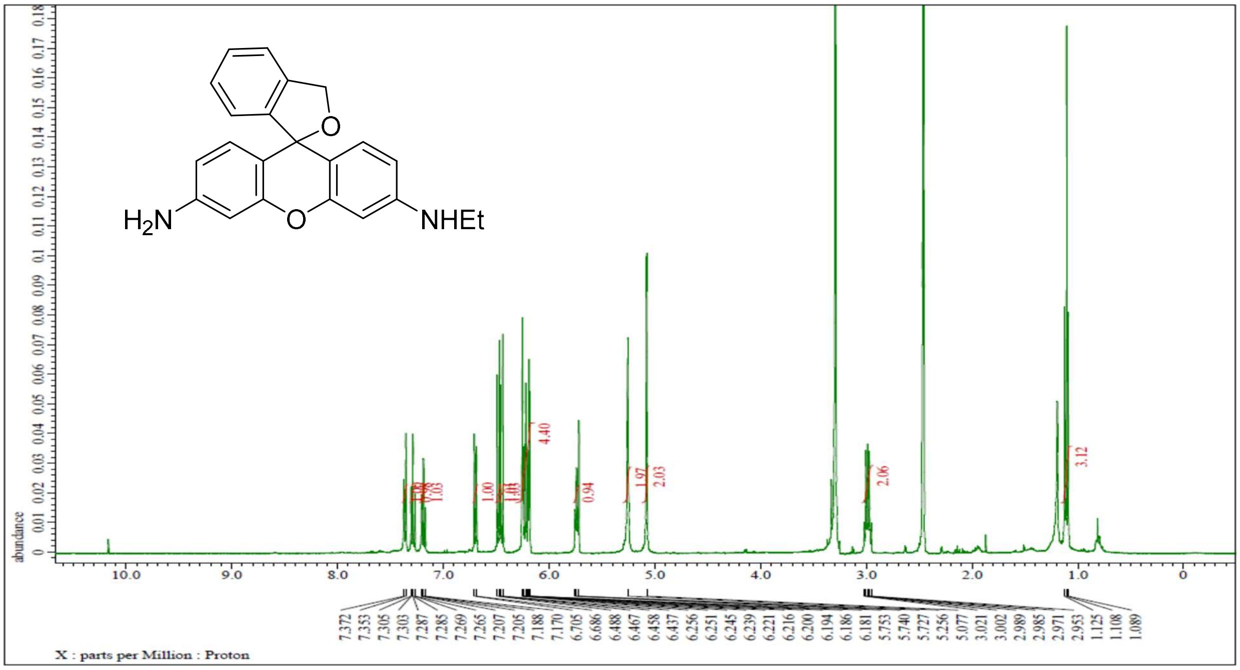

^{13}C NMR

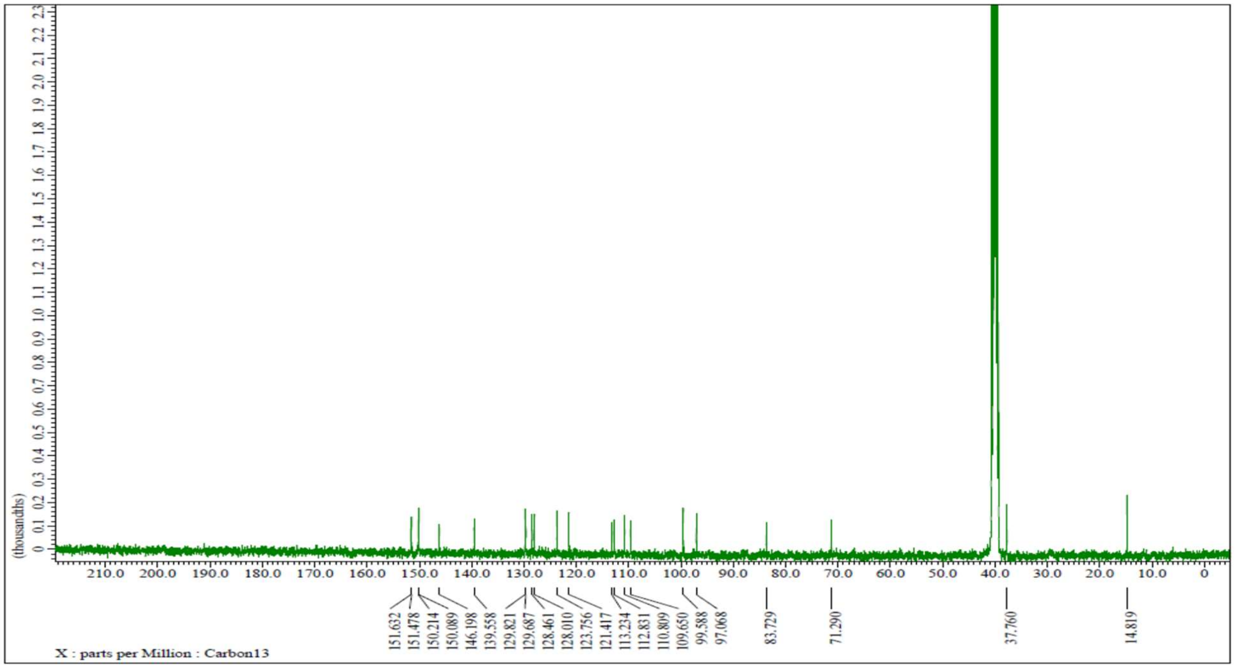

Mass Spectra

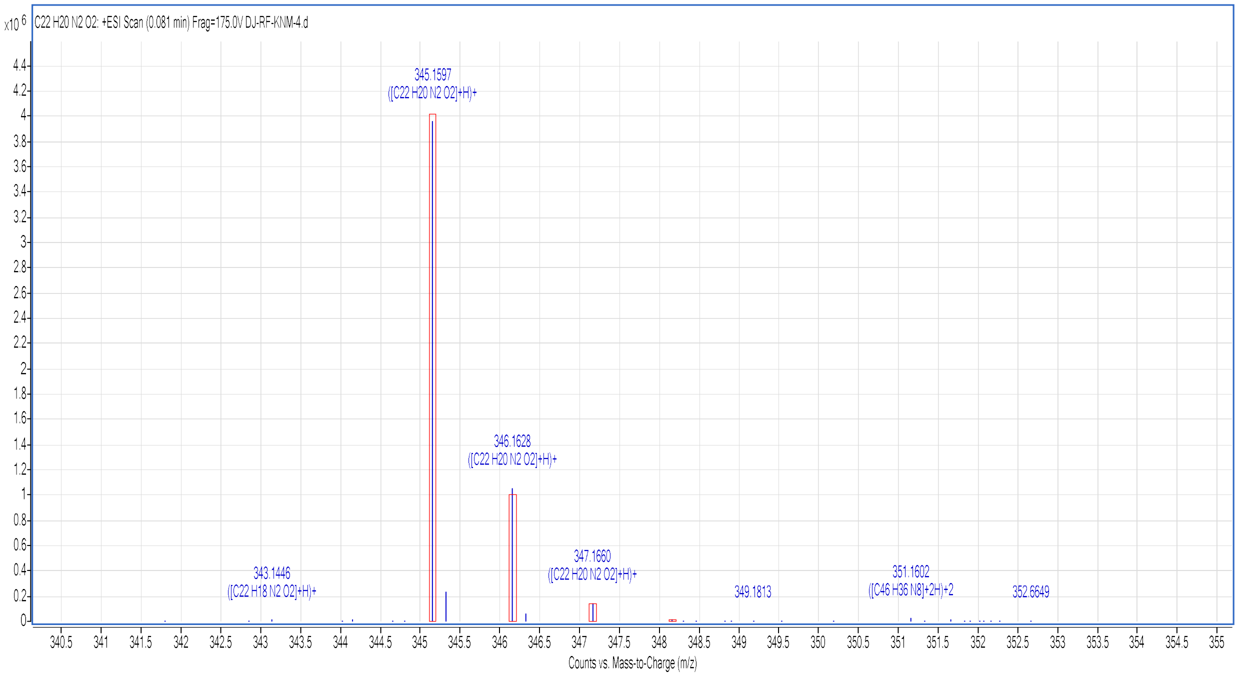

Figure S16: Structure, ^{1}H NMR, ^{13}C NMR and HRMS of Compound 17.

^{1}H NMR

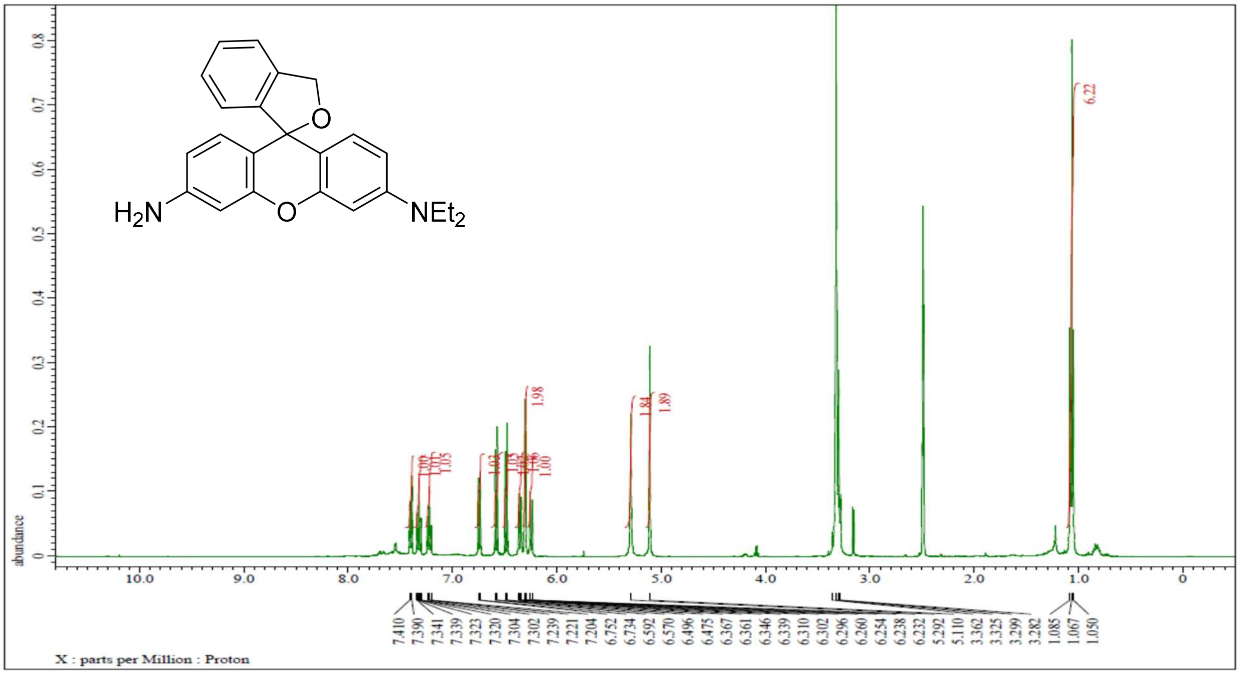

^{13}C NMR

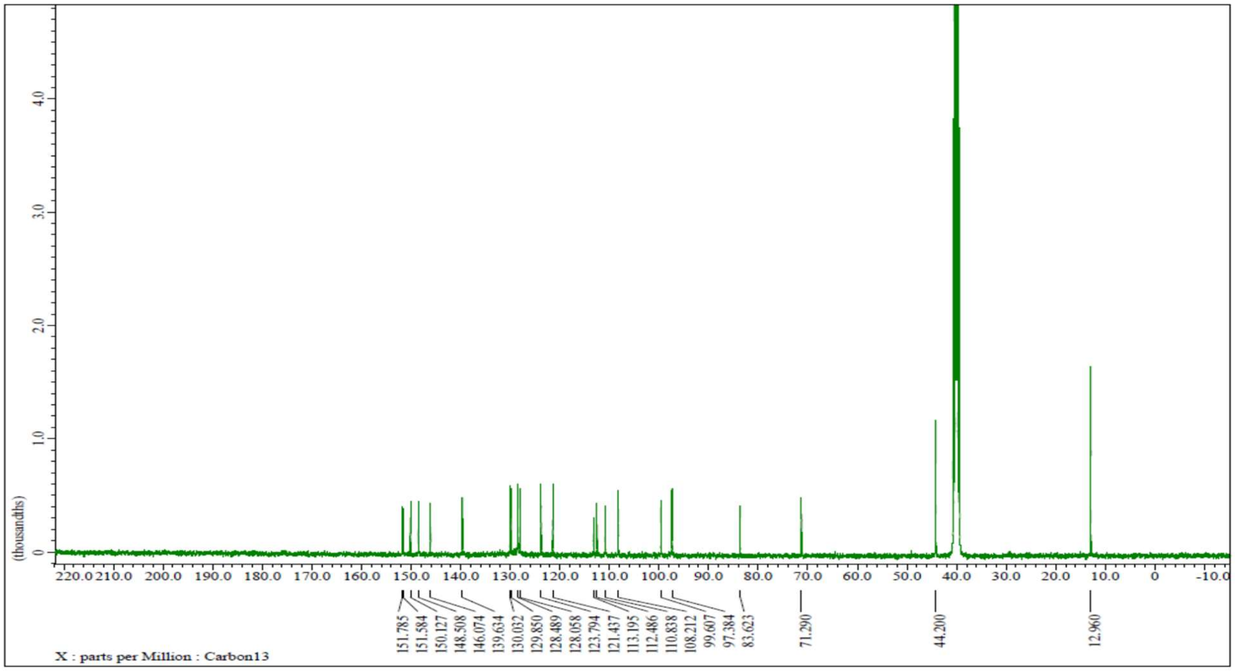

Mass Spectra

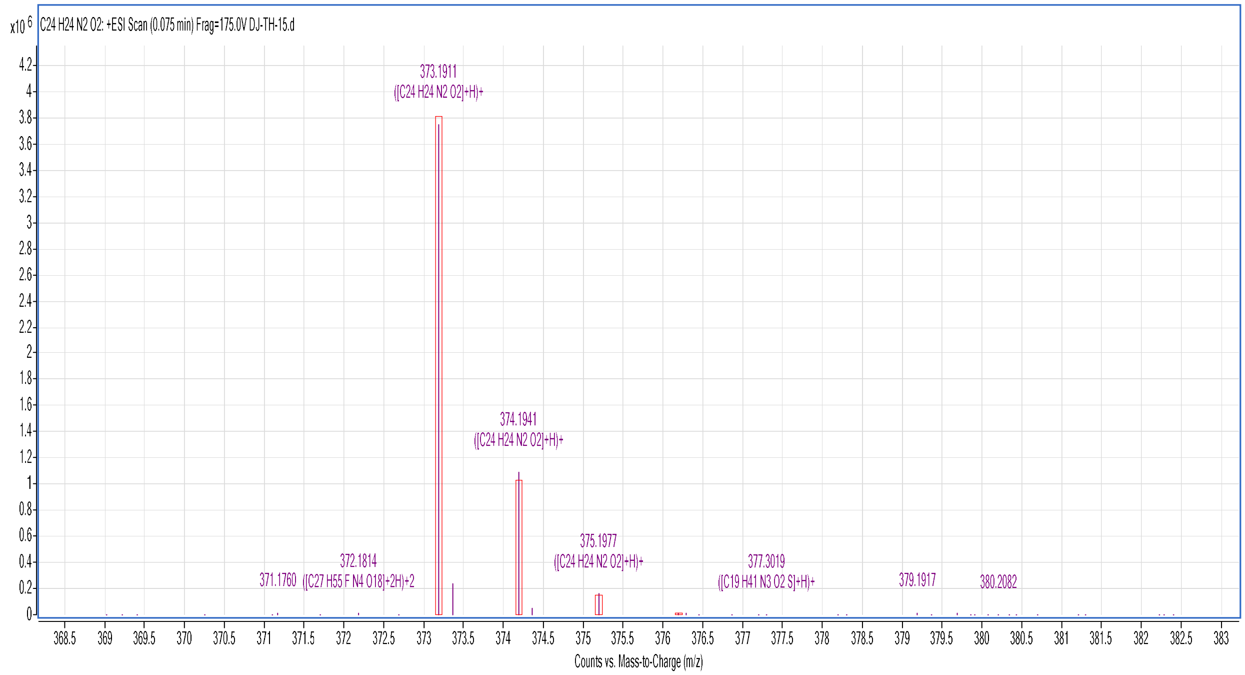

Figure S17: Structure, ^{1}H NMR, ^{13}C NMR and HRMS of Compound 18.

^{1}H NMR

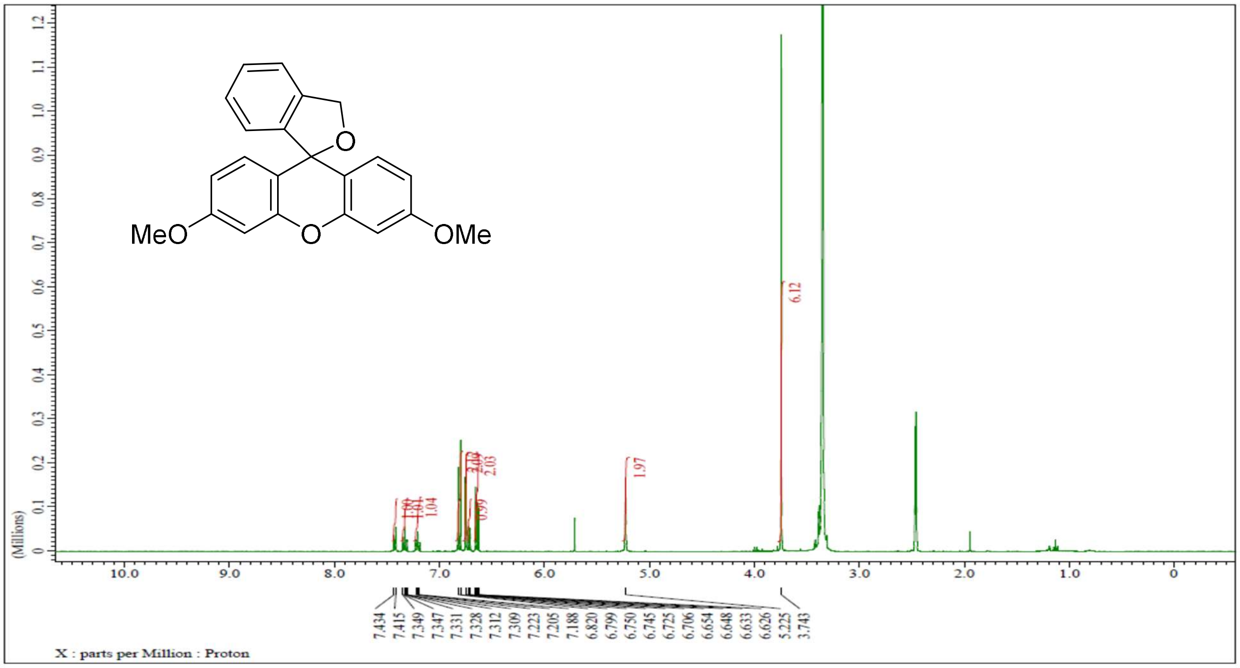

^{13}C NMR

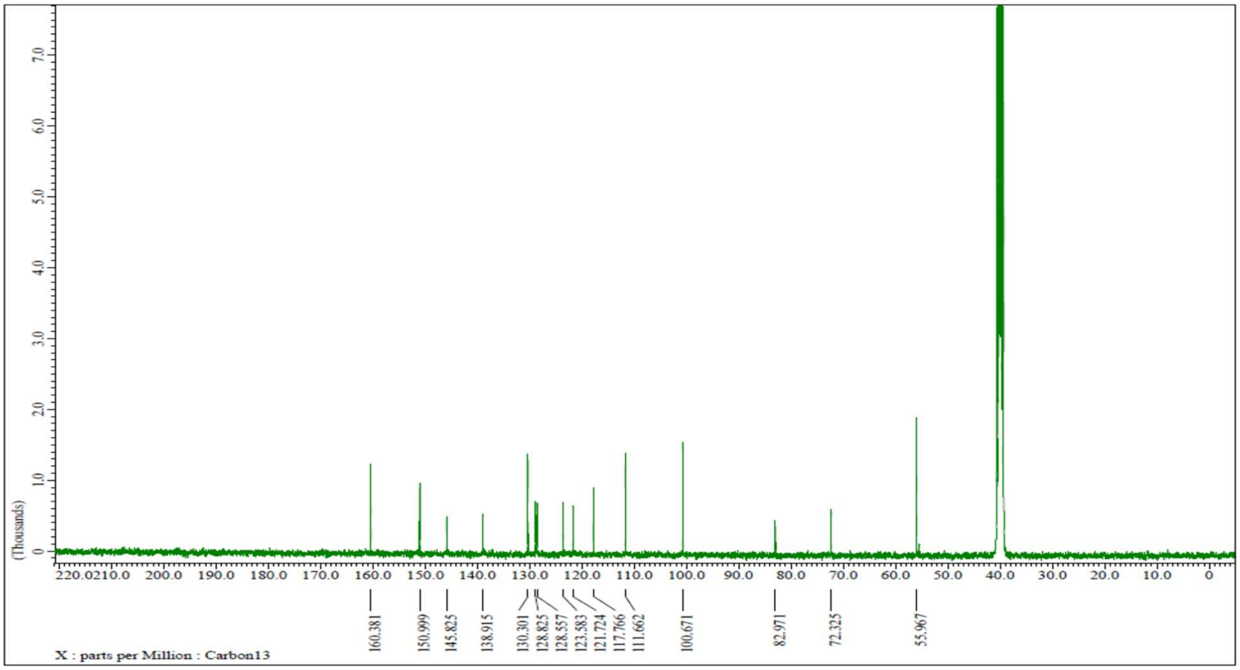

Mass Spectra

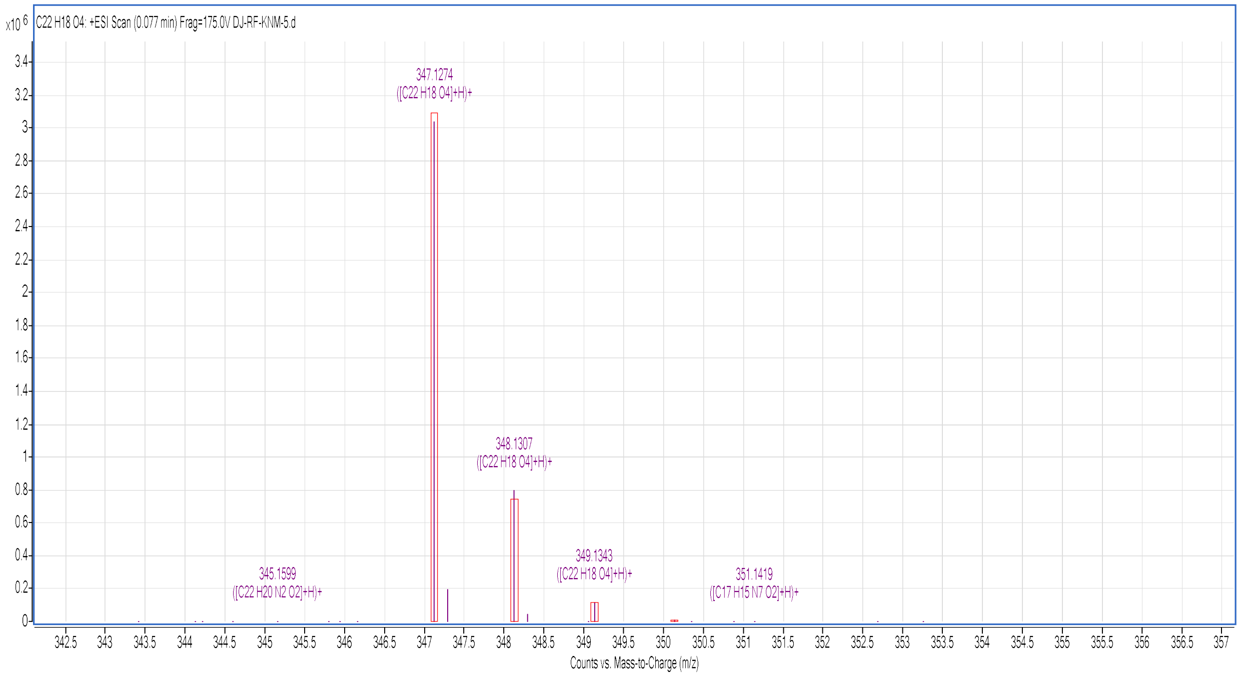

Figure S18: Structure, ^{1}H NMR, ^{13}C NMR and HRMS of Compound 19.

^{1}H NMR

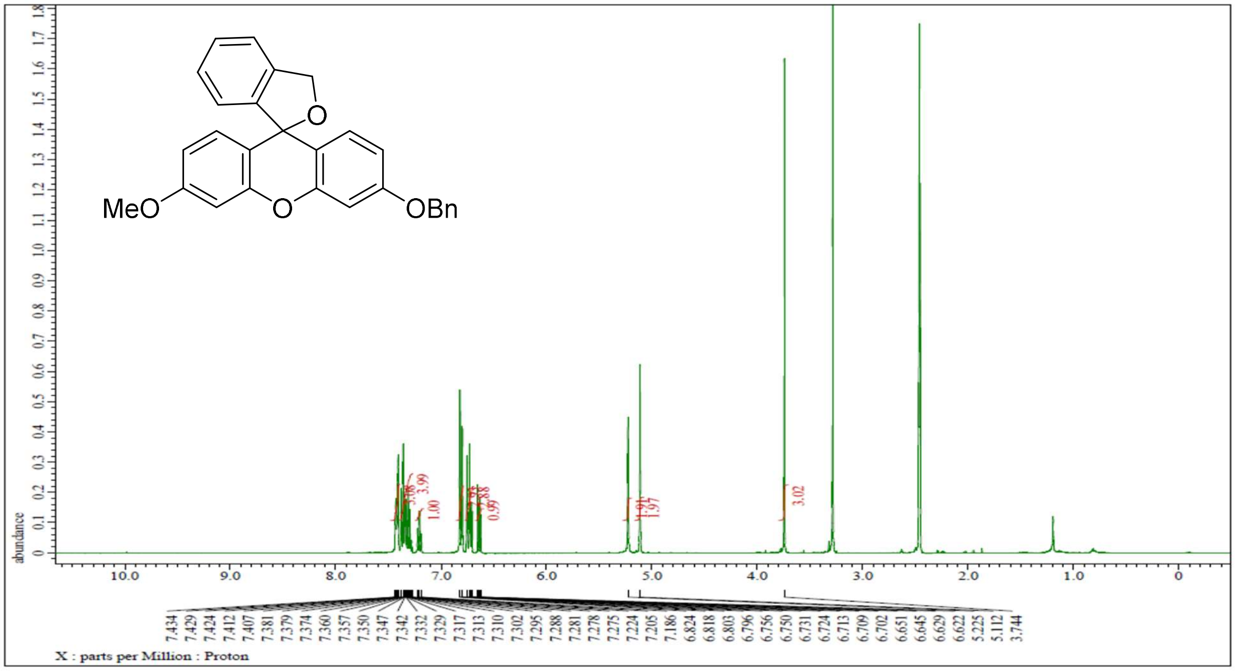

^{13}C NMR

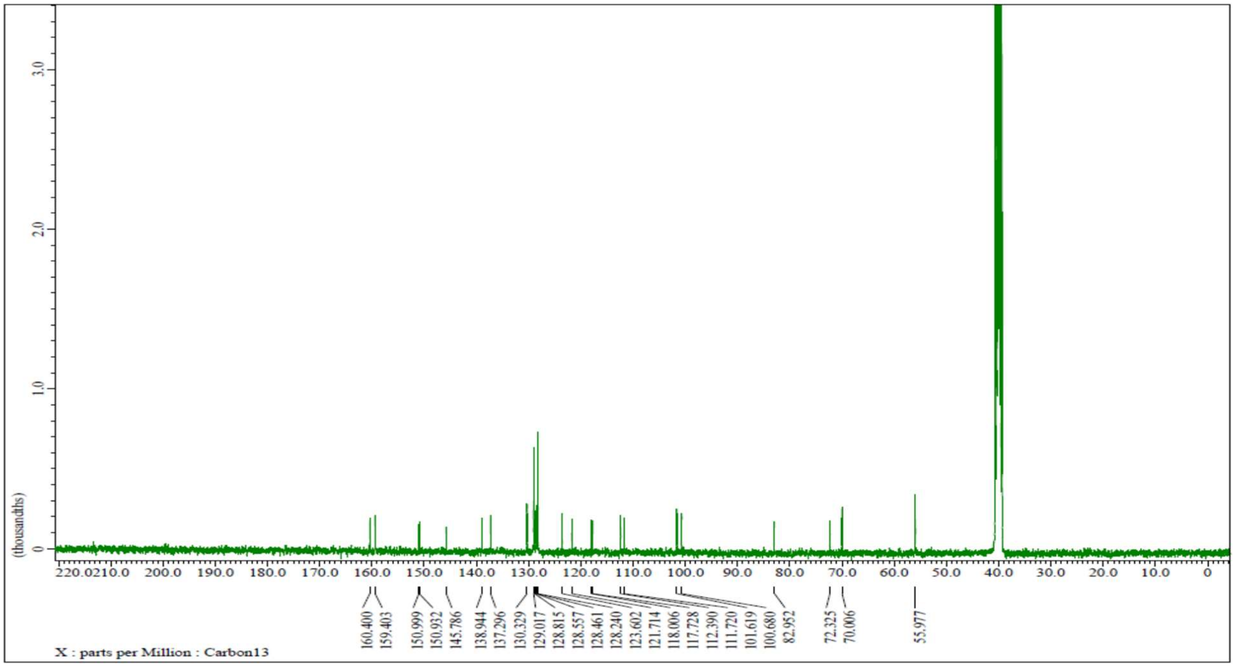

Mass Spectra

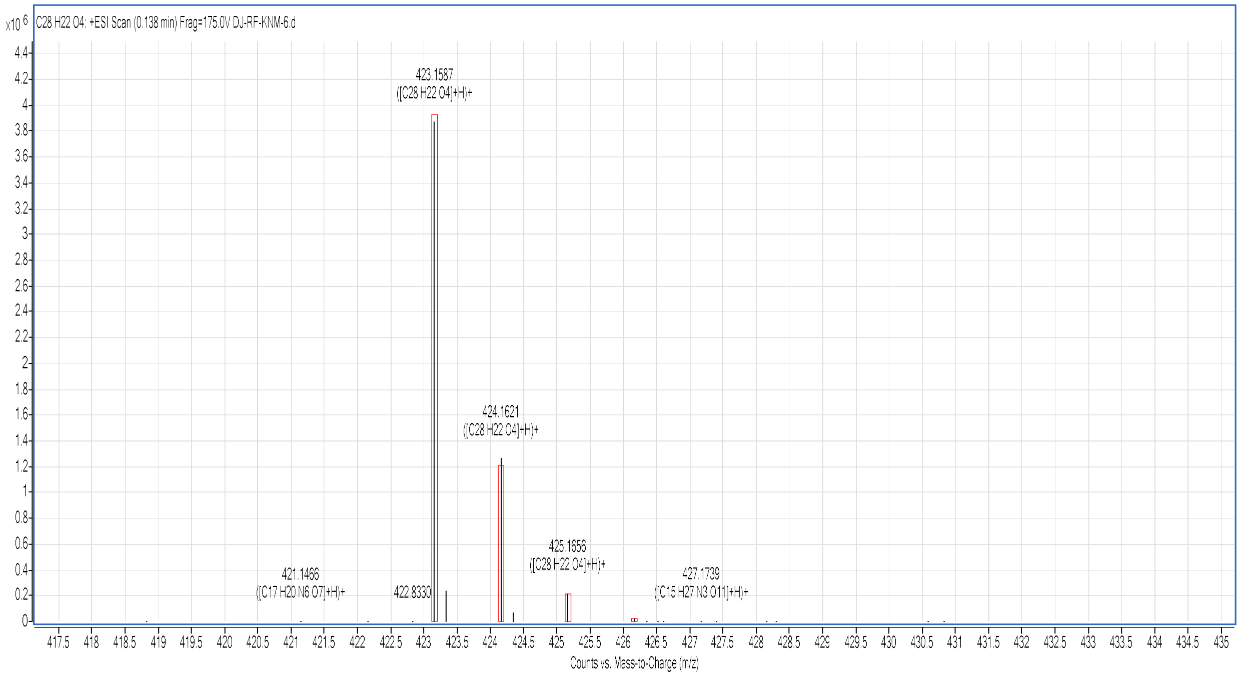

Figure S19: Structure, ^{1}H NMR, ^{13}C NMR and HRMS of Compound 20.

^{1}H NMR

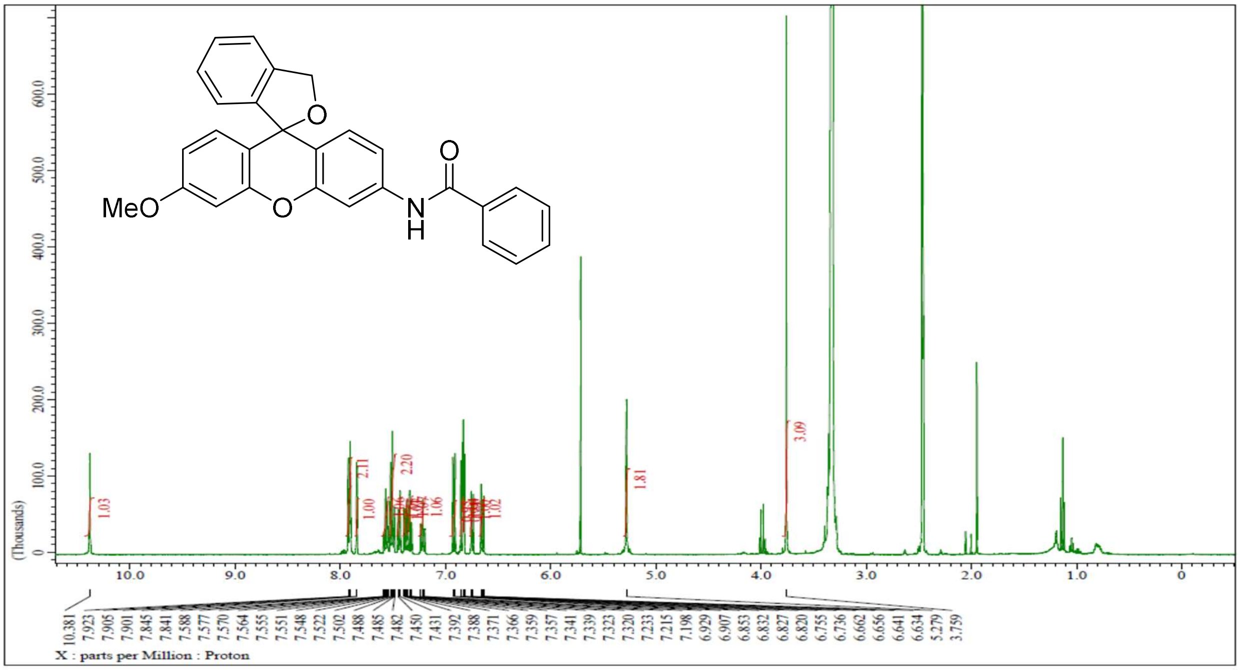

^{13}C NMR

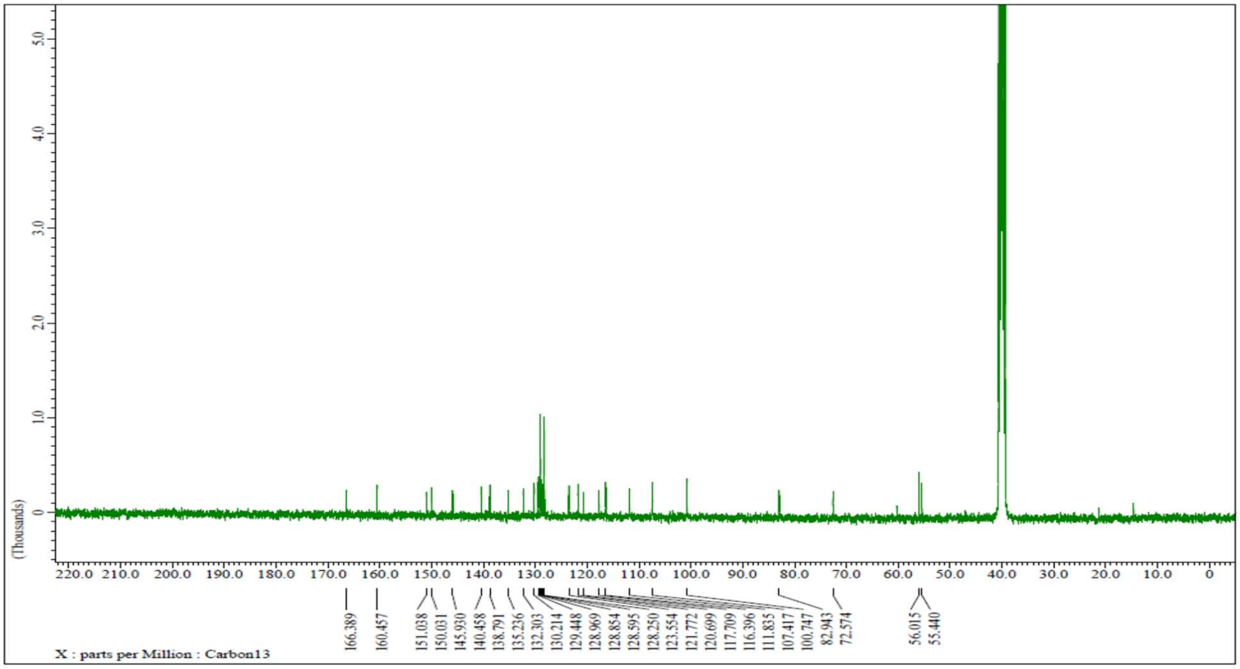

Mass Spectra

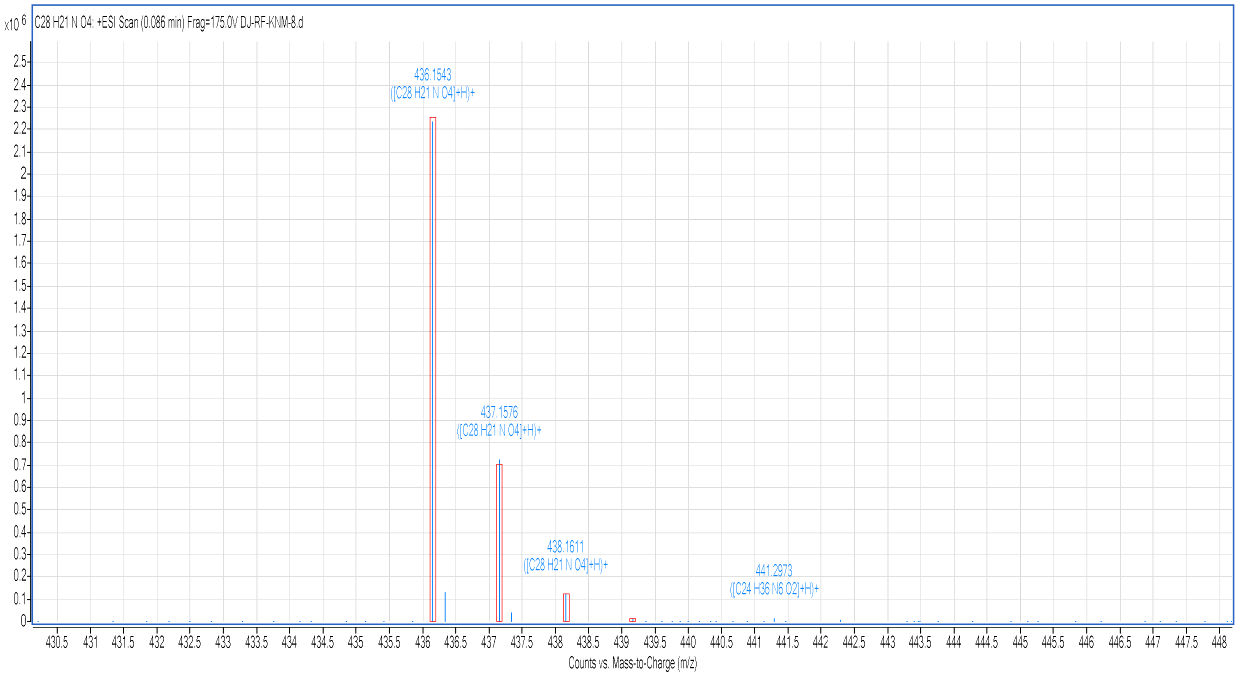

Figure S20: Structure, ^{1}H NMR, ^{13}C NMR and HRMS of Compound 21.

^1H NMR

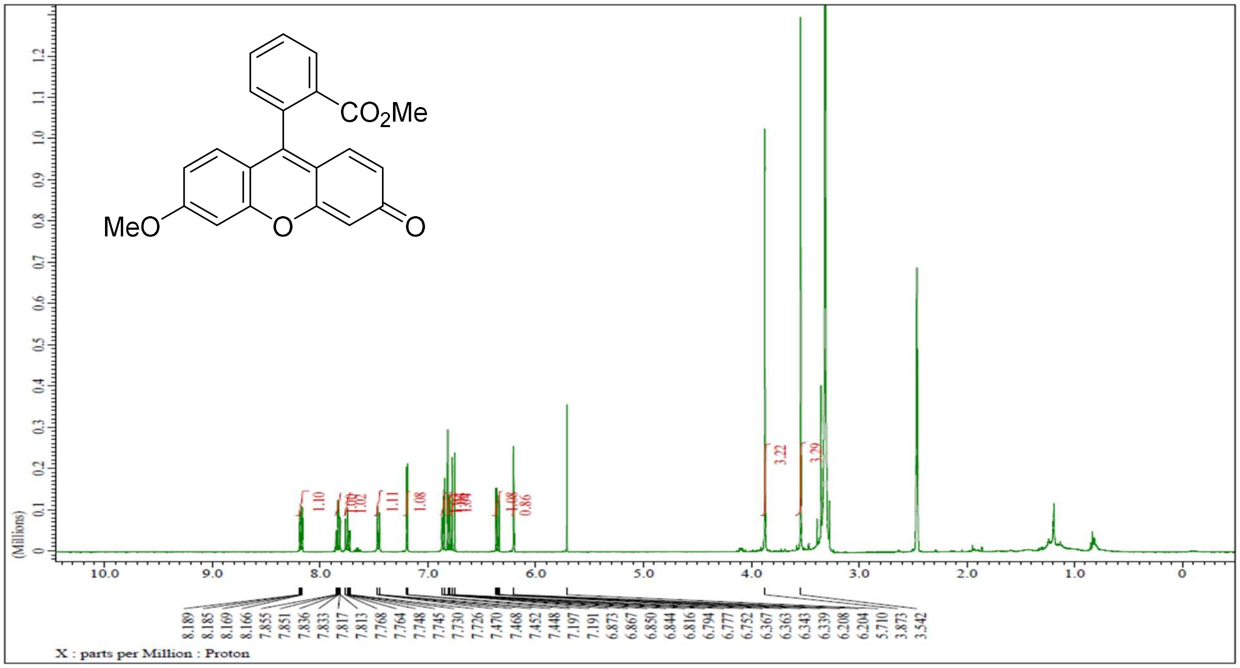

^{13}C NMR

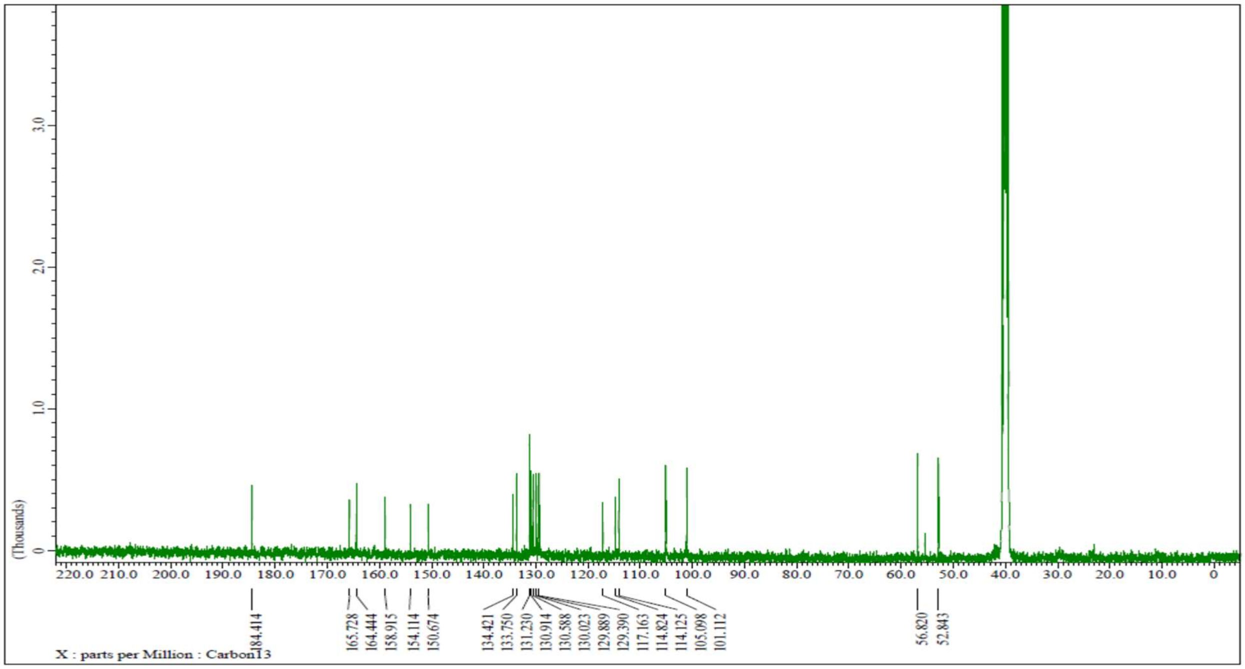

Mass Spectra

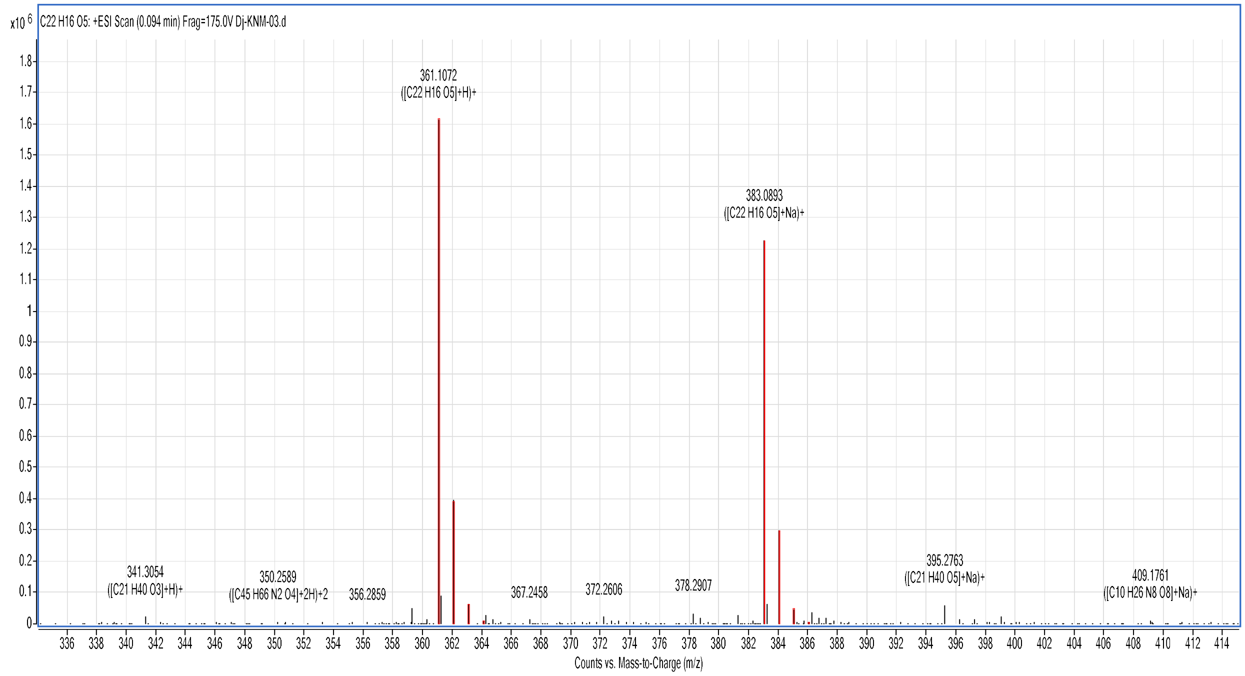

Figure S21: Structure, ^{1}H NMR, ^{13}C NMR and HRMS of Compound 24.

^{1}H NMR

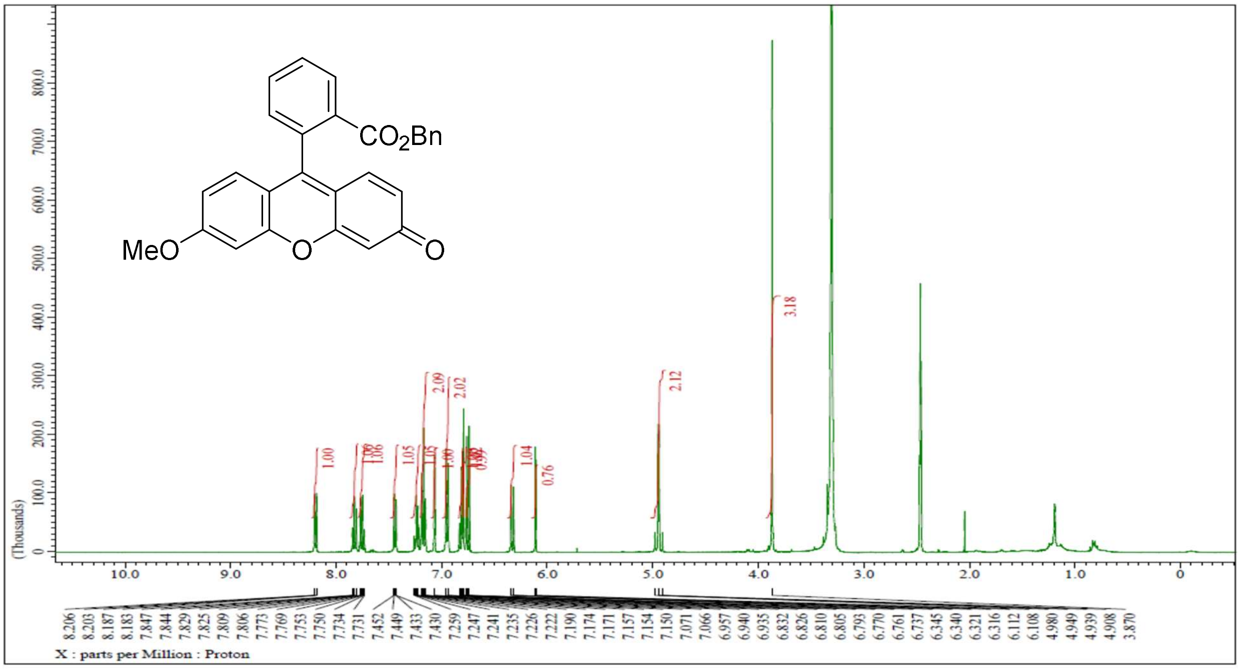

^{13}C NMR

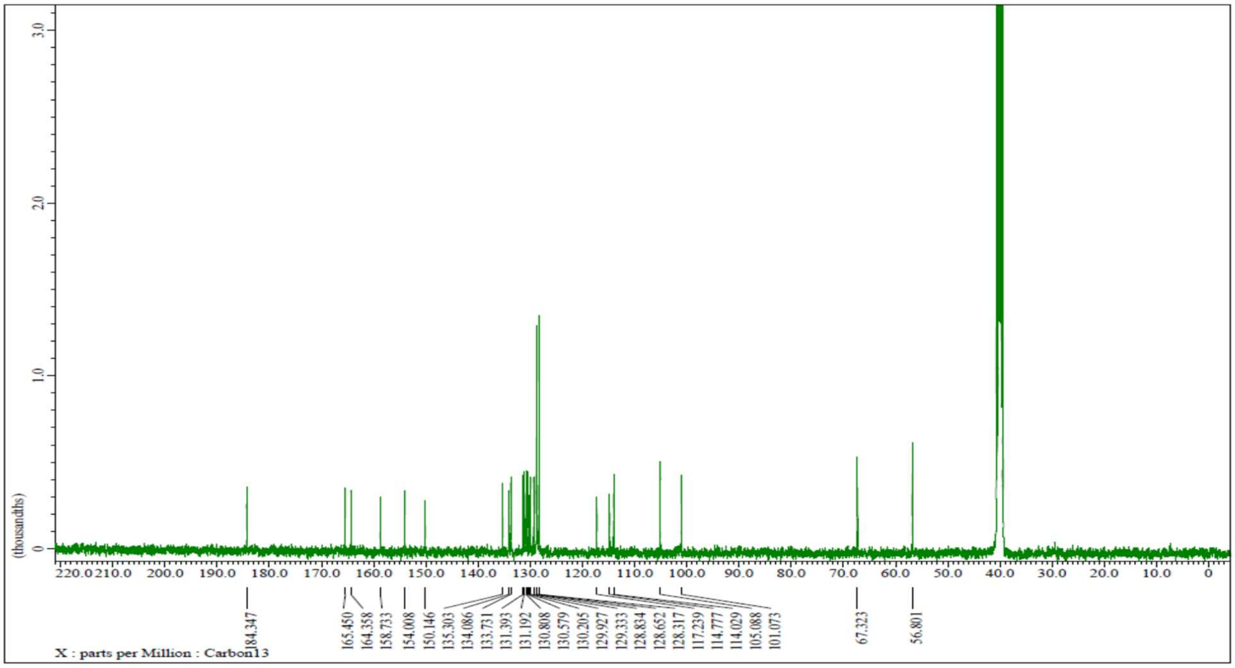

Mass Spectra

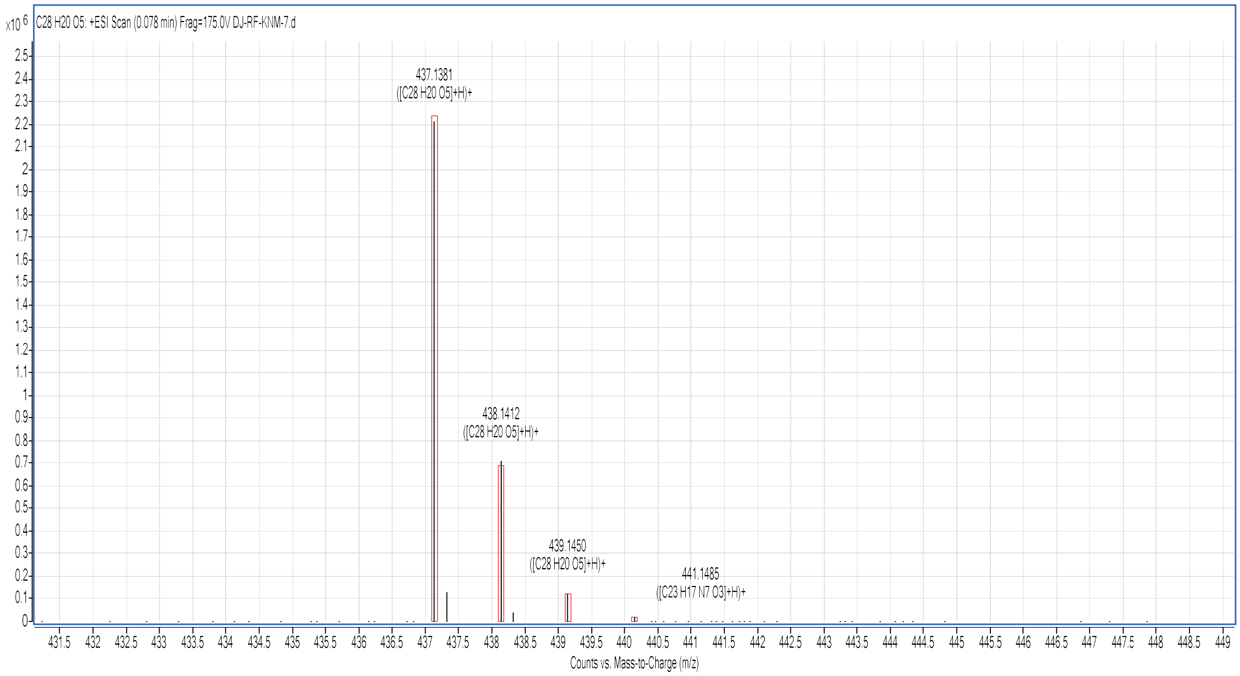

Figure S22: Structure, ^{1}H NMR, ^{13}C NMR and HRMS of Compound 25.

^{1}H NMR

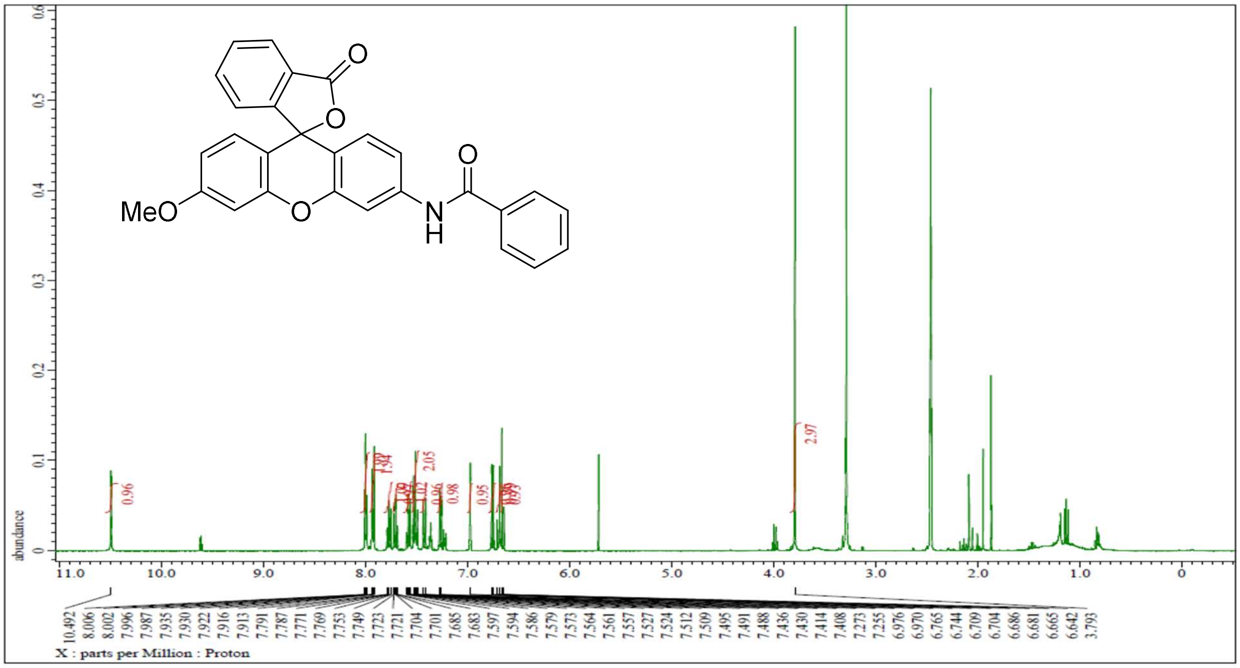

^{13}C NMR

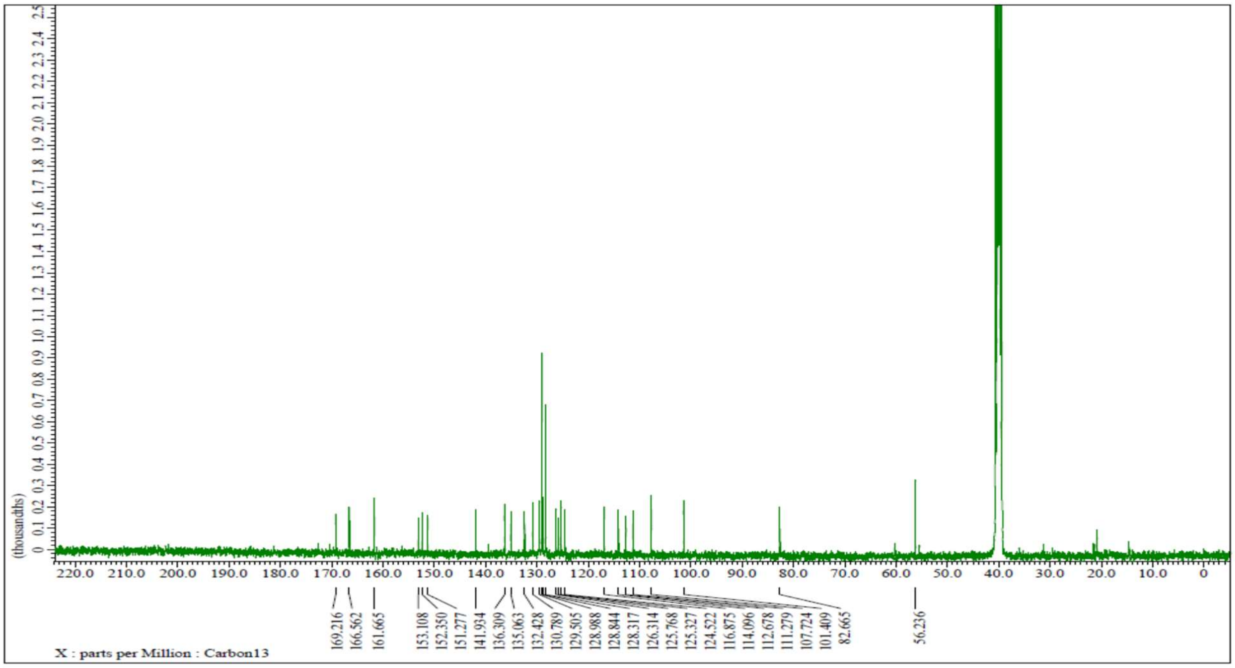

Mass Spectra

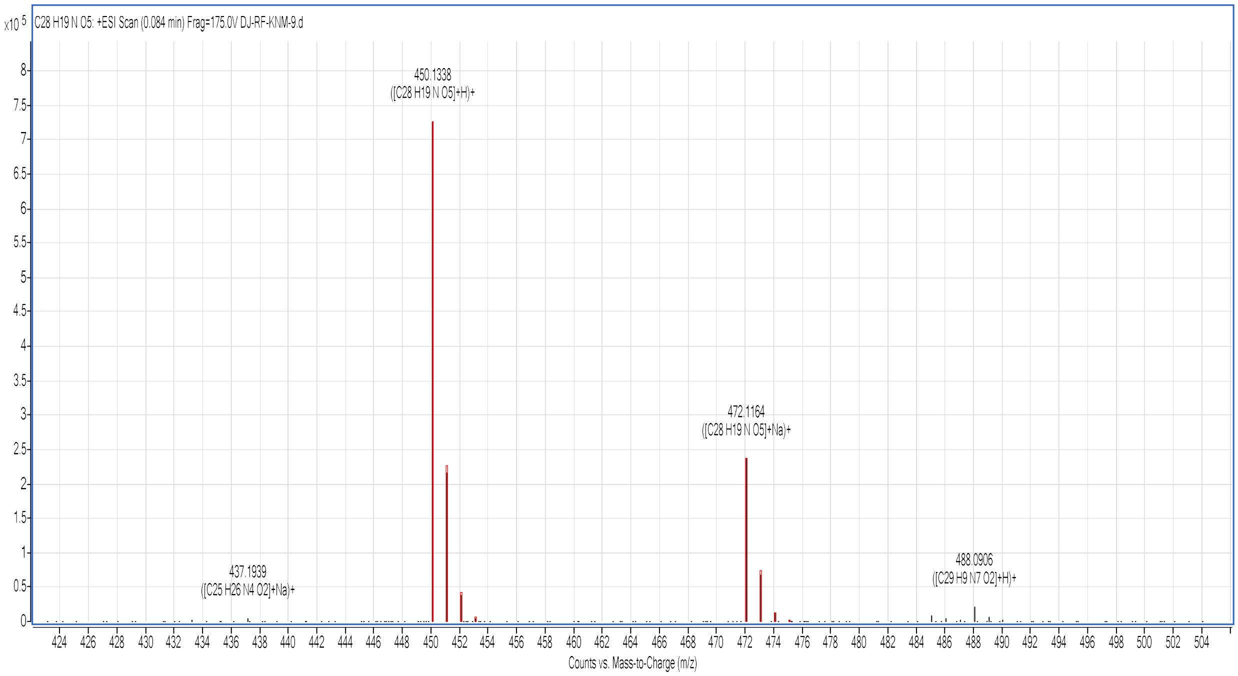

Figure S23: Structure, ^{1}H NMR, ^{13}C NMR and HRMS of Compound 26.

^{1}H NMR

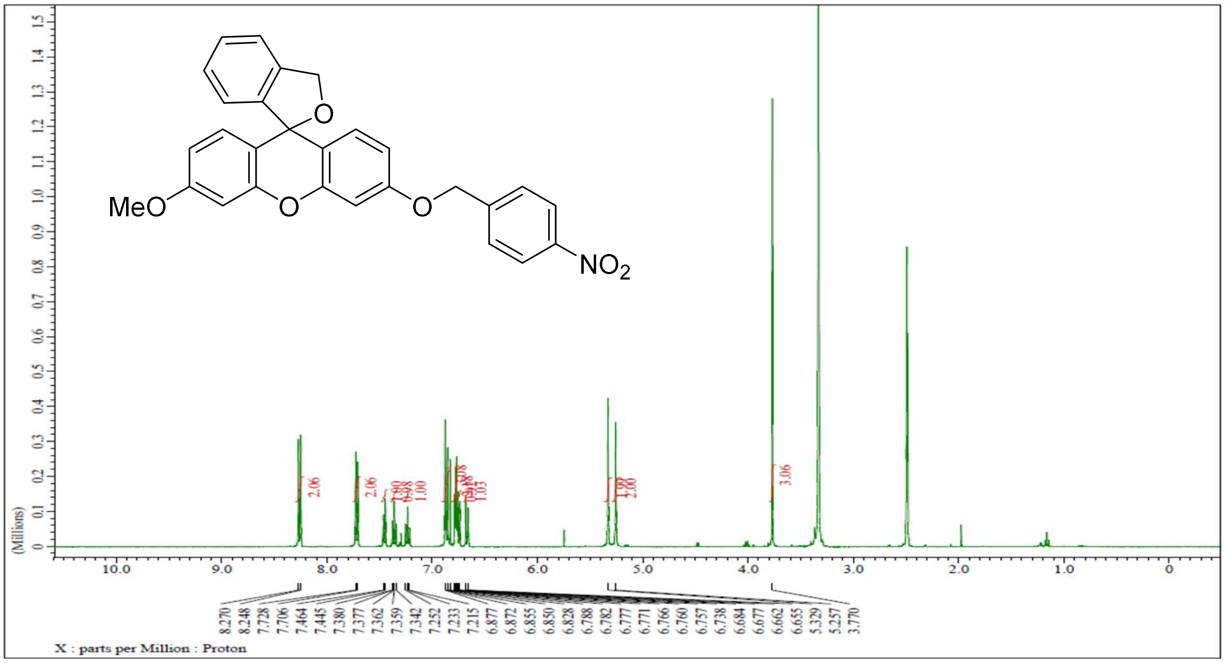

^{13}C NMR

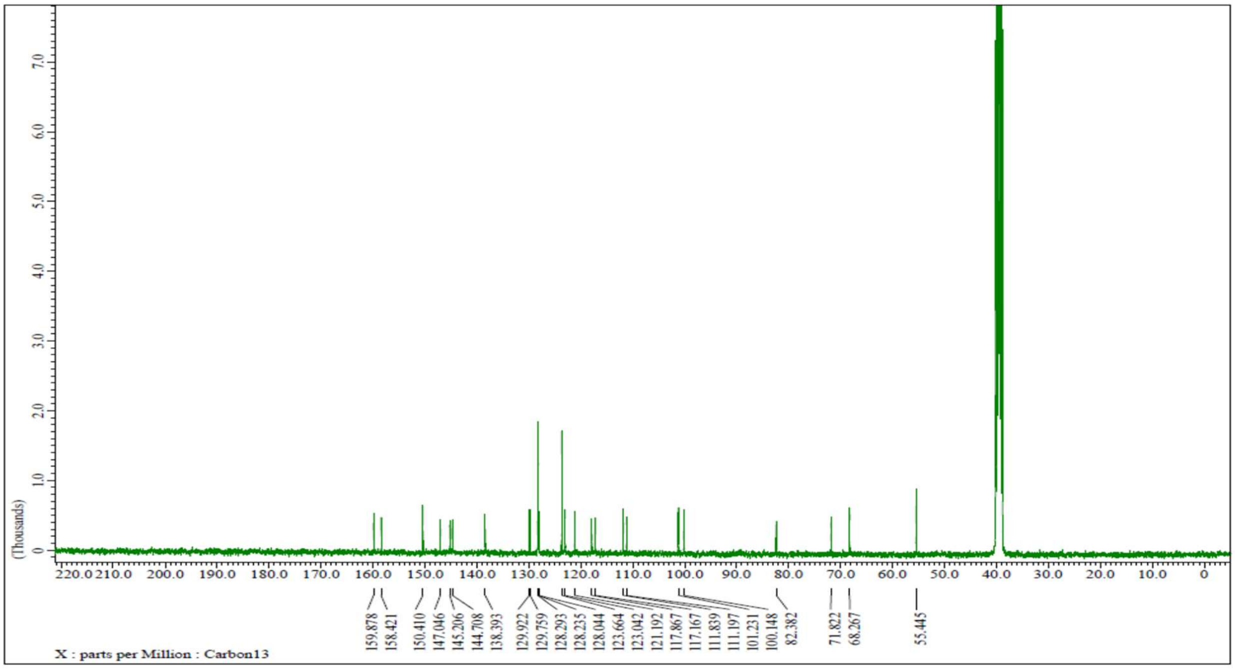

Mass Spectra

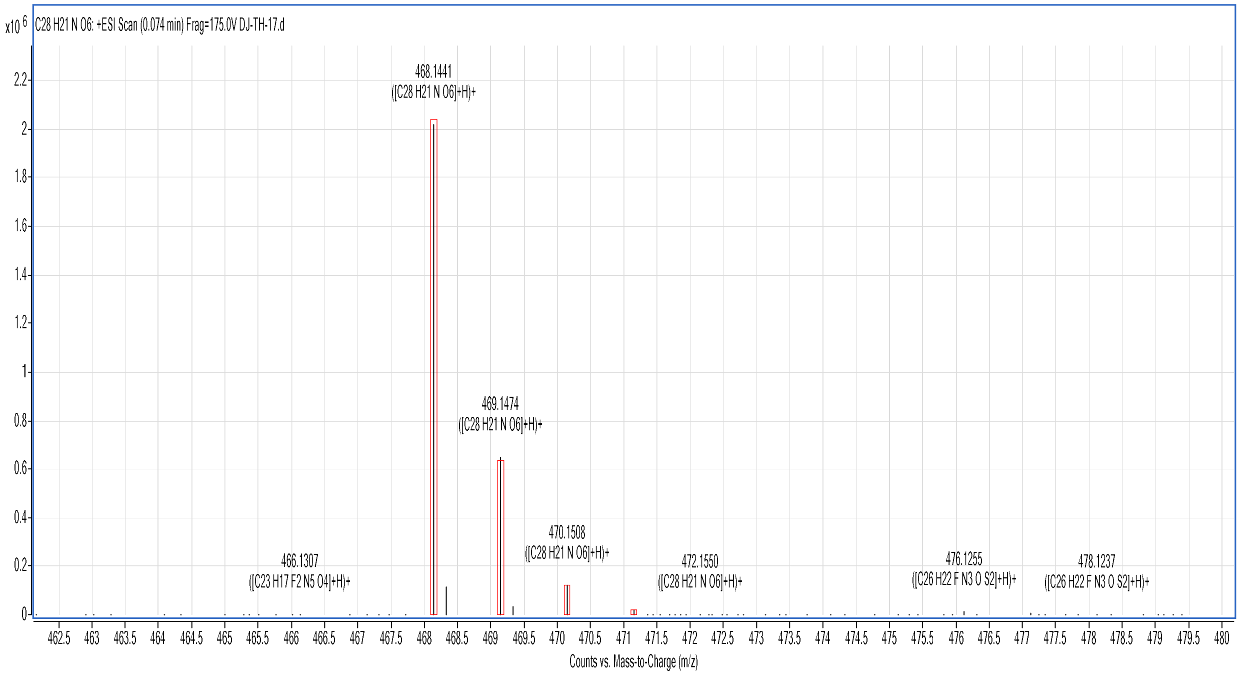

Figure S24: Structure, ^{1}H NMR, ^{13}C NMR and HRMS of Compound 27.

^{1}H NMR

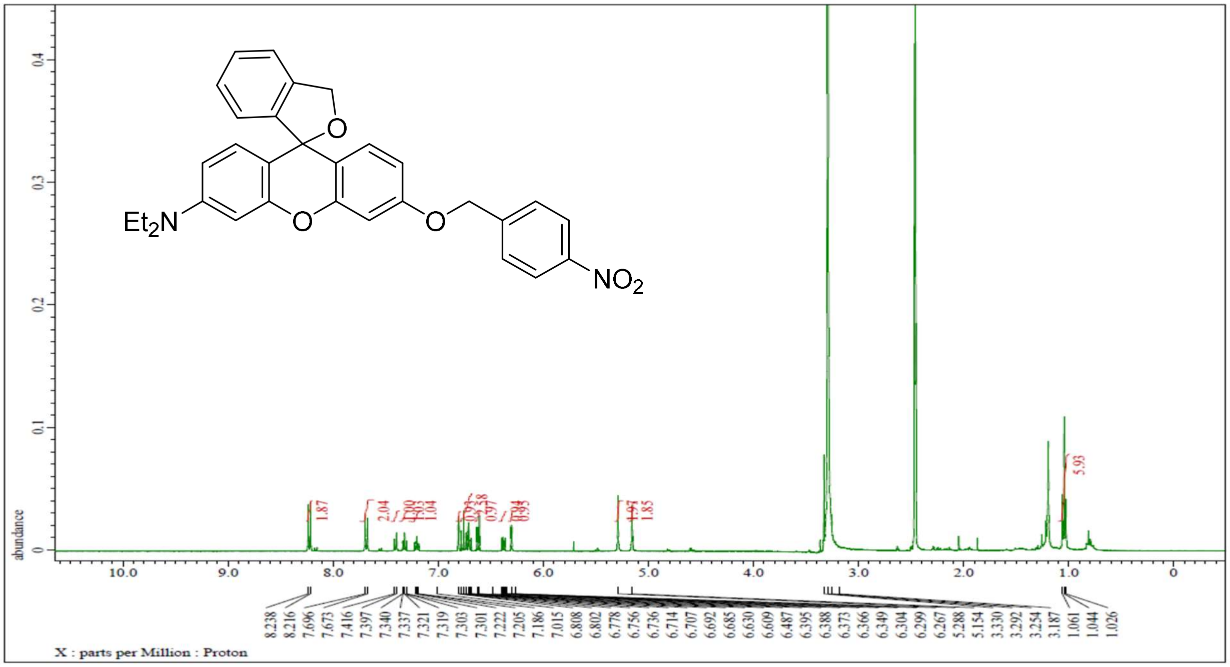

^{13}C NMR

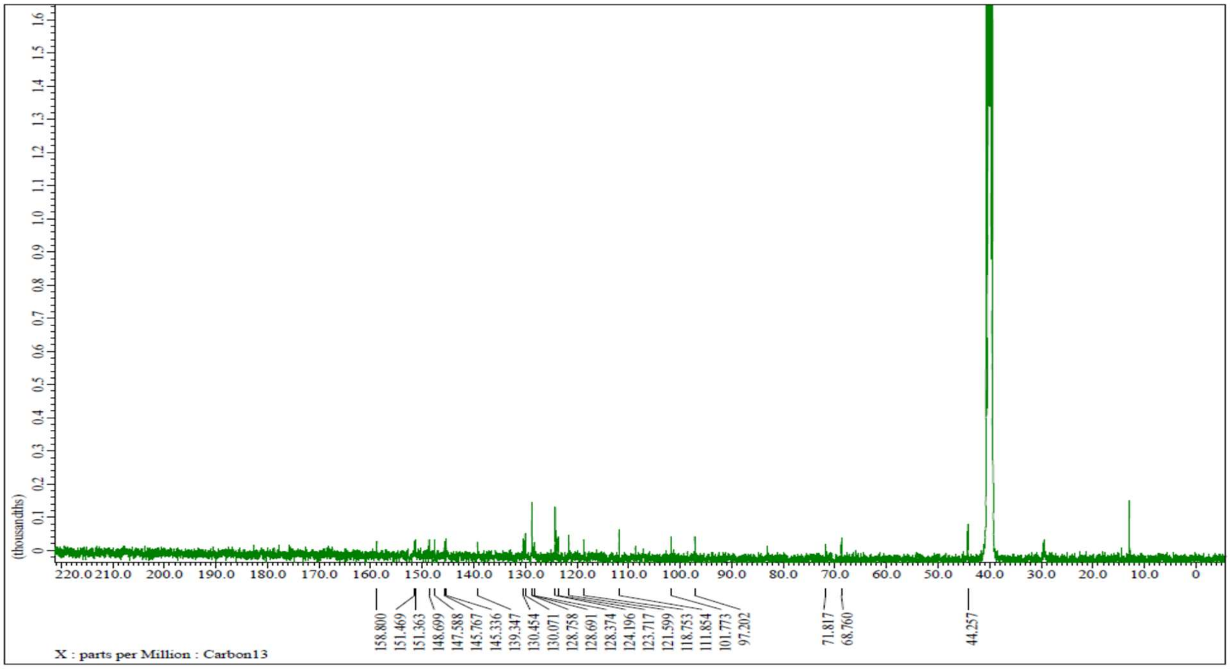

Mass Spectra

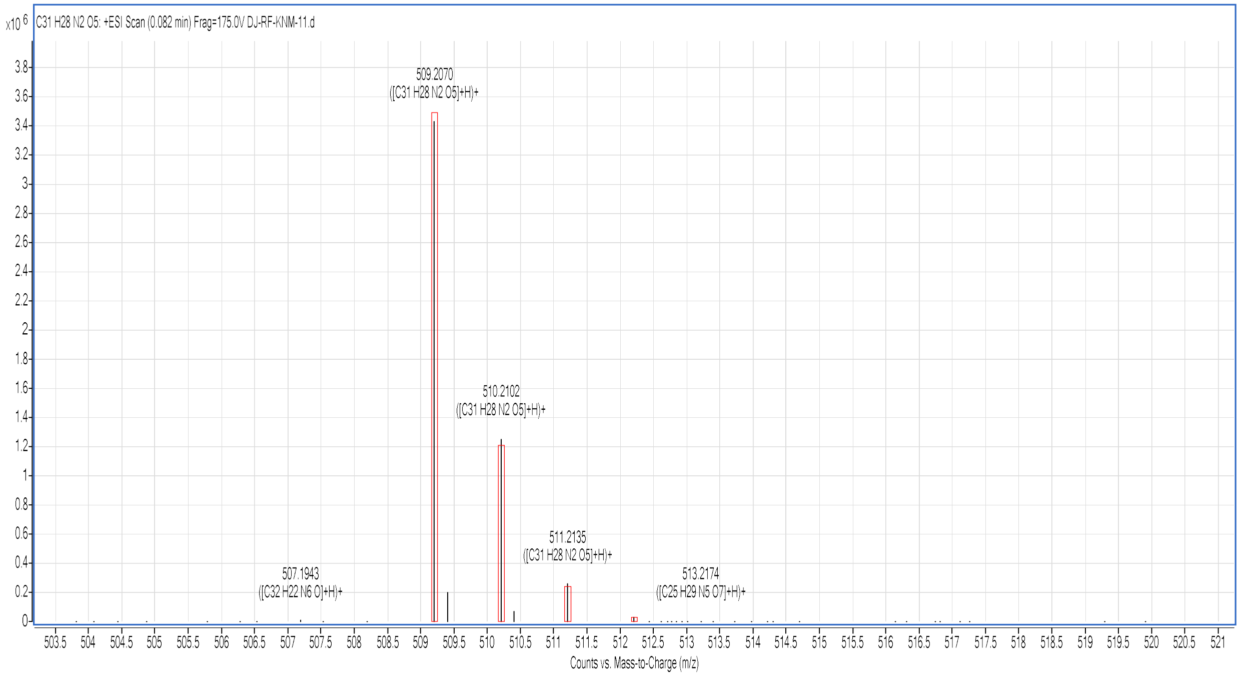

Figure S25: Structure, ^{1}H NMR, ^{13}C NMR and HRMS of Compound 28.

^{1}H NMR

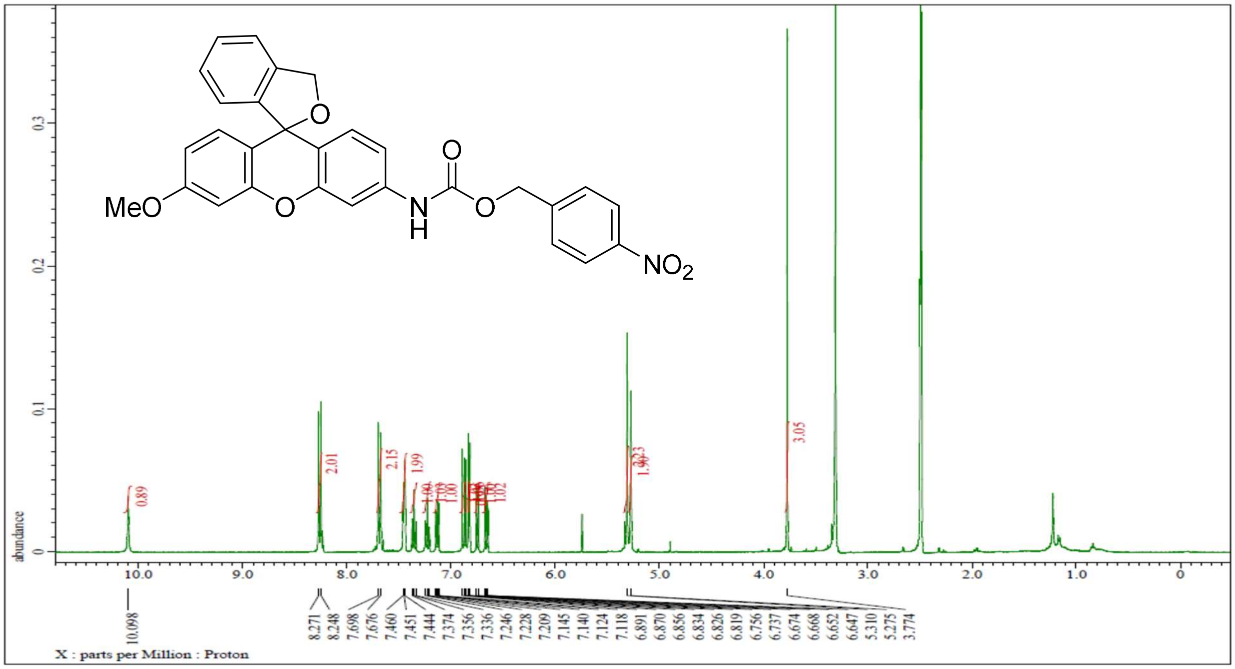

^{13}C NMR

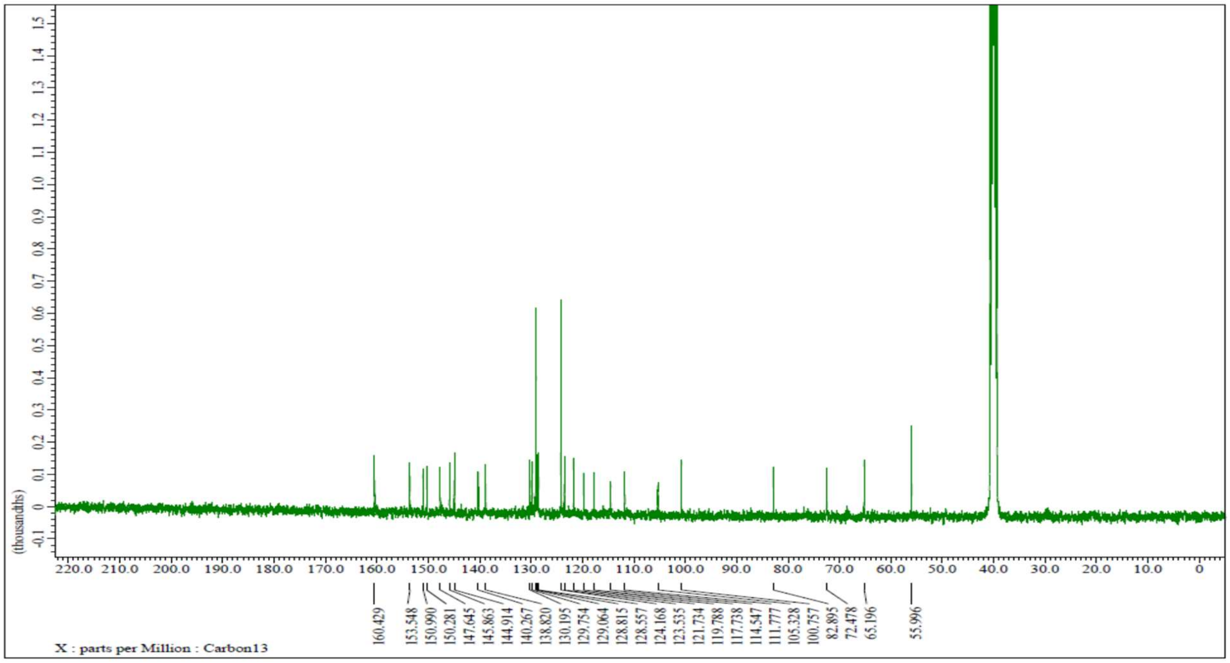

Mass Spectra

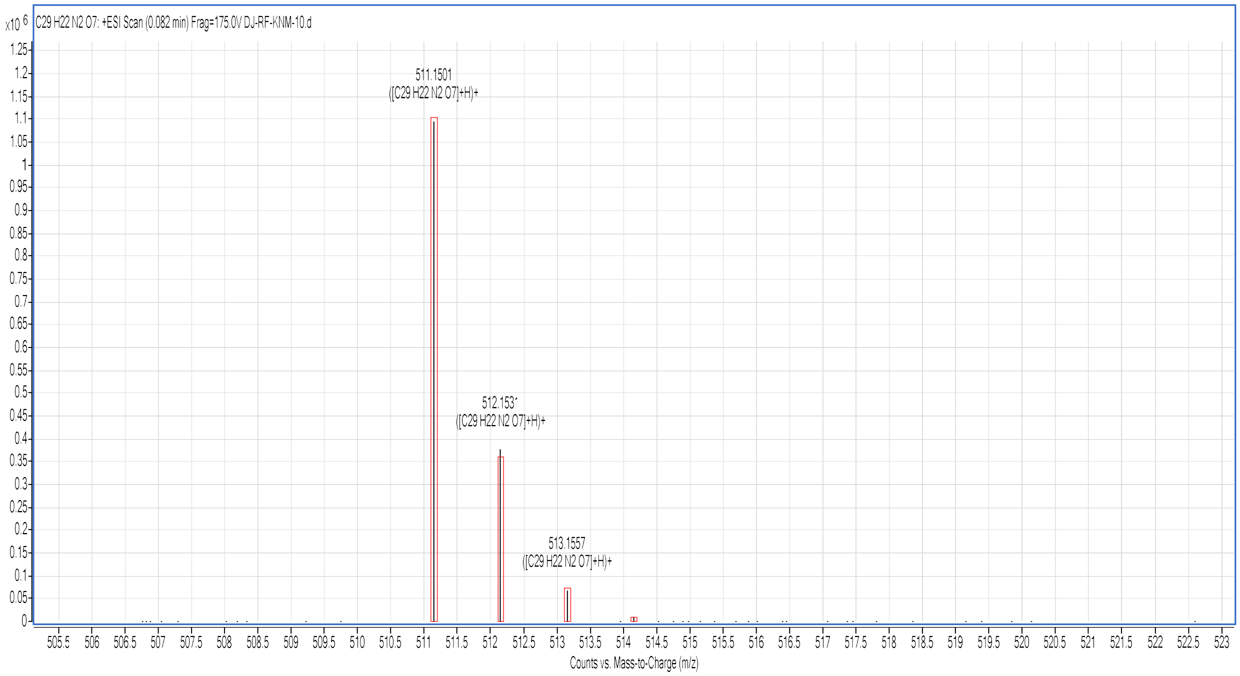

Figure S26: Structure, ^{1}H NMR, ^{13}C NMR and HRMS of Compound 29.

^{1}H NMR

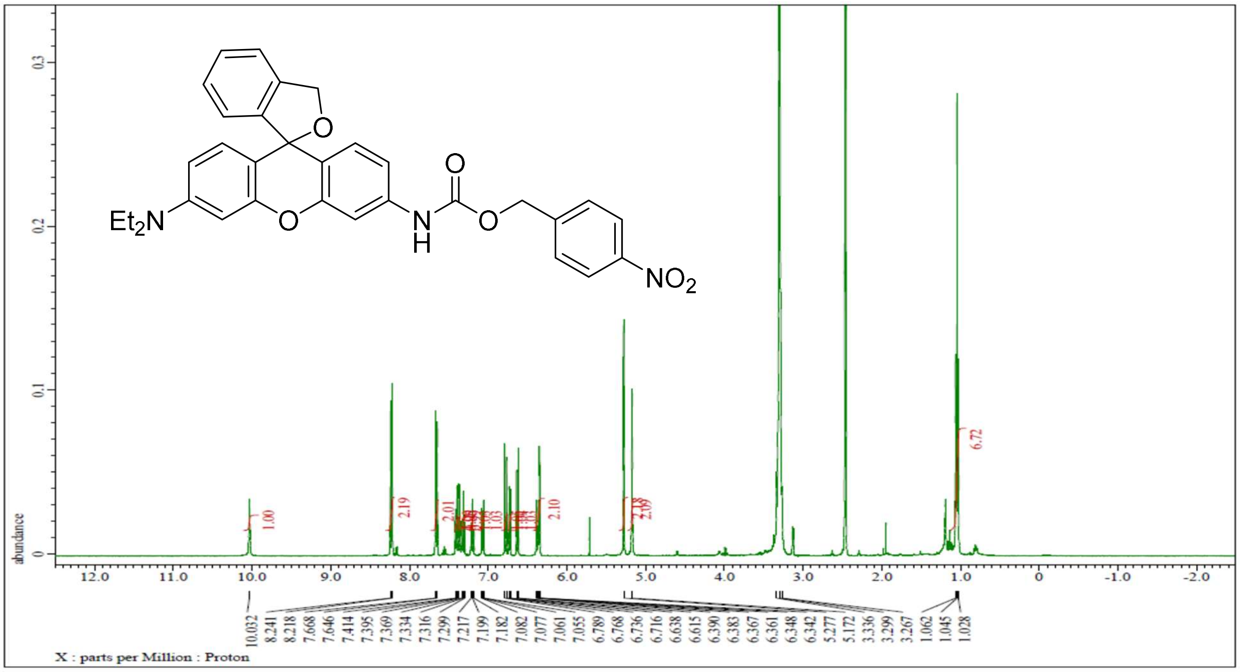

^{13}C NMR

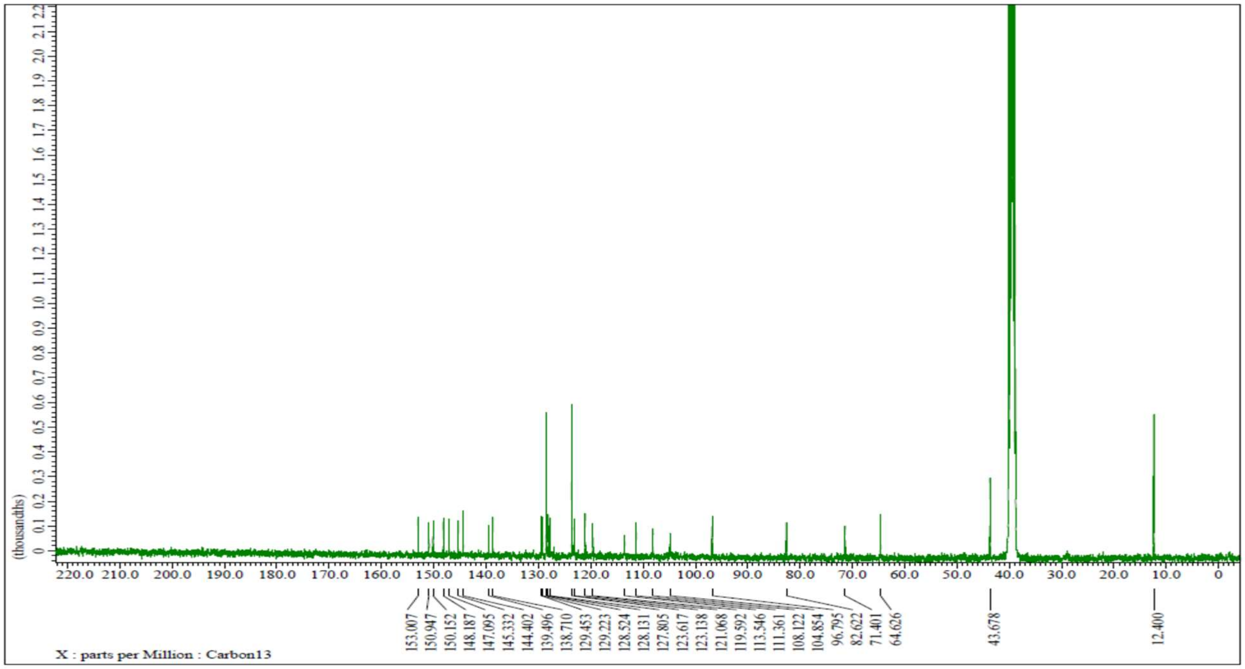

Mass Spectra

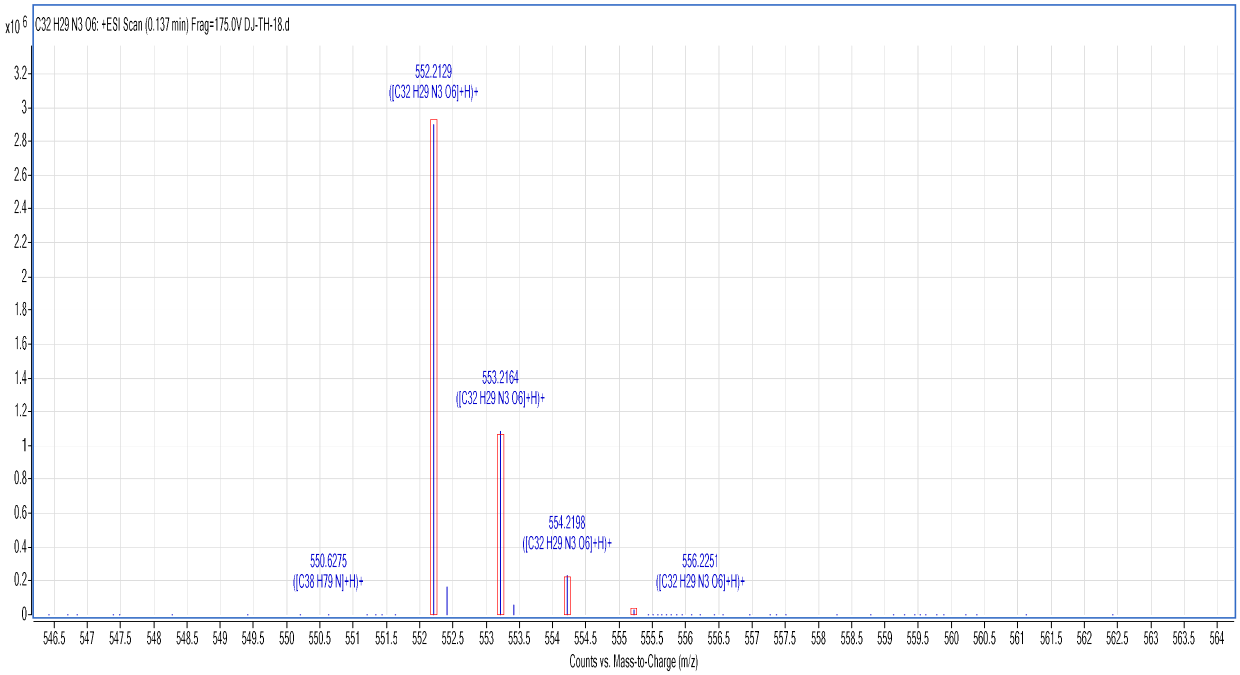

Figure S27: Structure, ¹H NMR, ¹³C NMR and HRMS of Compound 30.

References

1. Li, X.; Zhang, H.; Xie, Y.; Hu, Y.; Sun, H.; Zhu, Q. Fluorescent probes for detecting monoamine oxidase activity and cell imaging. *Org. Biomol. Chem.* **2014**, *12*, 2033-2036, doi:10.1039/C3OB42326C.

2. Yang, S.-H.; Sun, Q.; Xiong, H.; Liu, S.-Y.; Moosavi, B.; Yang, W.-C.; Yang, G.-F. Discovery of a butyrylcholinesterase-specific probe via a structure-based design strategy. *Chem. Commun.* **2017**, *53*, 3952-3955, doi:3[10.1039/C7CC00577F.
